# Recommendations on fit-for-purpose criteria to establish quality management for microphysiological systems and for monitoring their reproducibility

**DOI:** 10.1016/j.stemcr.2024.03.009

**Published:** 2024-04-25

**Authors:** David Pamies, Jason Ekert, Marie-Gabrielle Zurich, Olivier Frey, Sophie Werner, Monica Piergiovanni, Benjamin S. Freedman, Adrian Kee Keong Teo, Hendrik Erfurth, Darwin R. Reyes, Peter Loskill, Pelin Candarlioglu, Laura Suter-Dick, Shan Wang, Thomas Hartung, Sandra Coecke, Glyn N. Stacey, Beren Atac Wagegg, Eva-Maria Dehne, Francesca Pistollato, Marcel Leist

**Affiliations:** 1 Department of Biomedical Science, University of Lausanne, Lausanne, Switzerland; 2 Swiss Centre for Applied Human Toxicology (SCAHT), Basel, Switzerland; 3 Jason E Ekert: UCB Pharma, Cambridge, MA, USA; 4 InSphero AG, Schlieren, Switzerland; 5 University of Applied Sciences and Arts Northwestern Switzerland, School of Life Sciences, Muttenz, Switzerland; 6 Department of Pharmaceutical Sciences, University of Basel, Basel, Switzerland; 7 European Commission, Joint Research Centre (JRC), Ispra, Italy; 8 Division of Nephrology, Kidney Research Institute, and Institute for Stem Cell and Regenerative Medicine, Department of Medicine, University of Washington, Seattle, WA 98109, USA; 9 Plurexa LLC, Seattle, WA 98109, USA; 10 Stem Cells and Diabetes Laboratory, Institute of Molecular and Cell Biology (IMCB), Agency for Science, Technology and Research (A^∗^STAR), Proteos, Singapore, Singapore; 11 Department of Biochemistry, Yong Loo Lin School of Medicine, National University of Singapore, Singapore, Singapore; 12 Department of Medicine, Yong Loo Lin School of Medicine, National University of Singapore, Singapore, Singapore; 13 Precision Medicine Translational Research Programme (TRP), Yong Loo Lin School of Medicine, National University of Singapore, Singapore, Singapore; 14 TissUse GmbH, Berlin, Germany; 15 National Institute of Standards and Technology, Gaithersburg, MD, USA; 16 NMI Natural and Medical Sciences Institute at the University of Tübingen, Reutlingen, Germany; 17 Department for Microphysiological Systems, Institute of Biomedical Engineering, Faculty of Medicine, Eberhard Karls University Tübingen, Tübingen, Germany; 18 3R Center for In Vitro Models and Alternatives to Animal Testing, Eberhard Karls University Tübingen, Tübingen, Germany; 19 3D and 3Rs Ltd., 115c Milton Road, Cambridge CB4 1XE, UK; 20 Doerenkamp-Zbinden Professor and Chair for Evidence-based Toxicology, Johns Hopkins Bloomberg School of Public Health and Whiting School of Engineering, Baltimore, MD, USA; 21 CAAT Europe, University of Konstanz, Konstanz, Germany; 22 International Stem Cell Banking Initiative, 2 High Street, Barley, Herts SG88HZ, UK; 23 National Stem Cell Resource Centre, Institute of Zoology, Chinese Academy of Sciences, Beijing 100190, China; 24 Institute for Stem Cell and Regenerative Merdicine, Chinese Academy of Sciences, Beijing 100101, China; 25 Humane Society International, Rue Belliard 40, 1040 Bruxelles, Belgium; 26 In vitro Toxicology and Biomedicine, Department inaugurated by the Doerenkamp-Zbinden foundation, University of Konstanz, Konstanz, Germany

**Keywords:** microphysiological systems, organoids, organ on chip, spheroids, reproducibility, quality control, standards, criteria, recommendations, *in vitro*

## Abstract

Cell culture technology has evolved, moving from single-cell and monolayer methods to 3D models like reaggregates, spheroids, and organoids, improved with bioengineering like microfabrication and bioprinting. These advancements, termed microphysiological systems (MPSs), closely replicate tissue environments and human physiology, enhancing research and biomedical uses. However, MPS complexity introduces standardization challenges, impacting reproducibility and trust. We offer guidelines for quality management and control criteria specific to MPSs, facilitating reliable outcomes without stifling innovation. Our fit-for-purpose recommendations provide actionable advice for achieving consistent MPS performance.

## Teaser

Recent advances in cell culture technologies, particularly in microphysiological systems (MPSs), have transformed our approach to modeling human physiology. The challenge now lies in standardizing these innovations to guarantee reproducibility and maintain stakeholder trust. Our article offers a detailed roadmap for crafting quality management plans and setting precise quality control standards, specifically designed for the unique needs of MPS applications.

## Need for *in vitro* models

Over the past decade, there has been a noticeable increase in regulatory recognition of alternatives to animal testing methods in various countries, such as the US, EU, Canada, Brazil, Japan, and India (Parvatam et al., 2024). For instance, in Europe, non-animal testing methods, also known as new approach methodologies (NAMs), have been promoted for over 20 years. These methods have been incorporated into legislation, as seen in Directive 2001/83/EC (EUR-Lex 2001a) and Regulation (EU) 2019/6 (EUR-Lex 2001b). Furthermore, the “Resolution on plans and actions to accelerate the transition to innovation without the use of animals in research, regulatory testing, and education” and the “JOINT MOTION FOR A RESOLUTION (https://www.europarl.europa.eu/doceo/document/RC-9-2021-0425_EN.html) on plans and actions to accelerate the transition to innovation without the use of animals in research, regulatory testing, and education” by the European Union have emphasized the importance of these advancements. Additionally, there has been acceptance and adoption of several guidelines on *in vitro* methods from the Organization for Economic Cooperation and Development (OECD), such as OECD TG 442E for in vitro skin sensitization (OECD 2023). Furthermore, countries like the USA and India have recognized microphysiological systems (MPSs) as a crucial part of these new methods. In December 2022, President Biden signed into law the bill for the US Food and Drug Administration Modernization Act 2.0 that “… allows an applicant for market approval for a new drug to use methods other than animal testing to establish the drug’s safety and effectiveness. Under this bill, these alternative methods may include cell-based assays, organ chips and microphysiological systems, computer modeling, and other human biology-based test methods” (FDA 2021).

*In vitro* methods possess unique characteristics that are crucial in advancing biomedical research. These methods facilitate the in-depth study of human diseases, toxicity, and human pathogen effects, offering insights beyond the reach of other approaches. In many cases, NAMs deliver data faster and with lower costs. A particularly important point is their reliance on human cells, which are considered to best reflect human physiology. MPSs, which encompass organoids, spheroids, microfluidics, and other complex systems, particularly emphasize this aspect by providing cells with a tissue-like environment, circulation (similar to blood flow), and enabling the connection of multiple tissues. The downside of the increased complexity is that these systems are more challenging to standardize, and that measures to guarantee the reproducibility of data from MPSs are particularly critical. Here, we present the concept of a quality management (QM) plan specifically tailored to avoid reproducibility issues with MPSs and to increase confidence in the use of MPS-derived data by the broader community.

It is important to note that, in the context of this paper, standardization is mainly discussed in terms of quality control for the cellular components of an MPS. However, this component of MPSs is very complex due to the variability of phenotype and function for each cellular model. It is also important to consider that, since MPSs encompass microfabrication and microfluidic systems, the engineering component of MPSs—which includes physical features (e.g., geometry, surface topology) and materials (e.g., porosity, chemistry)—is also an important aspect to consider.

## Existing guidance relevant to MPS

New complex cell cultures have been developed *in vitro* with the goal of overcoming the limitations associated with traditional cell cultures (Pamies and Hartung 2017). These new models, known as MPSs or complex *in vitro* models (CIVMs), aim to better replicate specific tissue architecture and organ functionality (Roth and MPS-WS Berlin 2019, 2021). For example, cerebral organoids are able to recapitulate the hallmarks of human neurodevelopment, including ventricular zone structures that contain apical radial glia, subventricular zone areas that contain intermediate progenitors and outer radial glia, and an emerging cortical plate that contains neurons, impossible otherwise with classical 2D methods.

**Figure 1:**
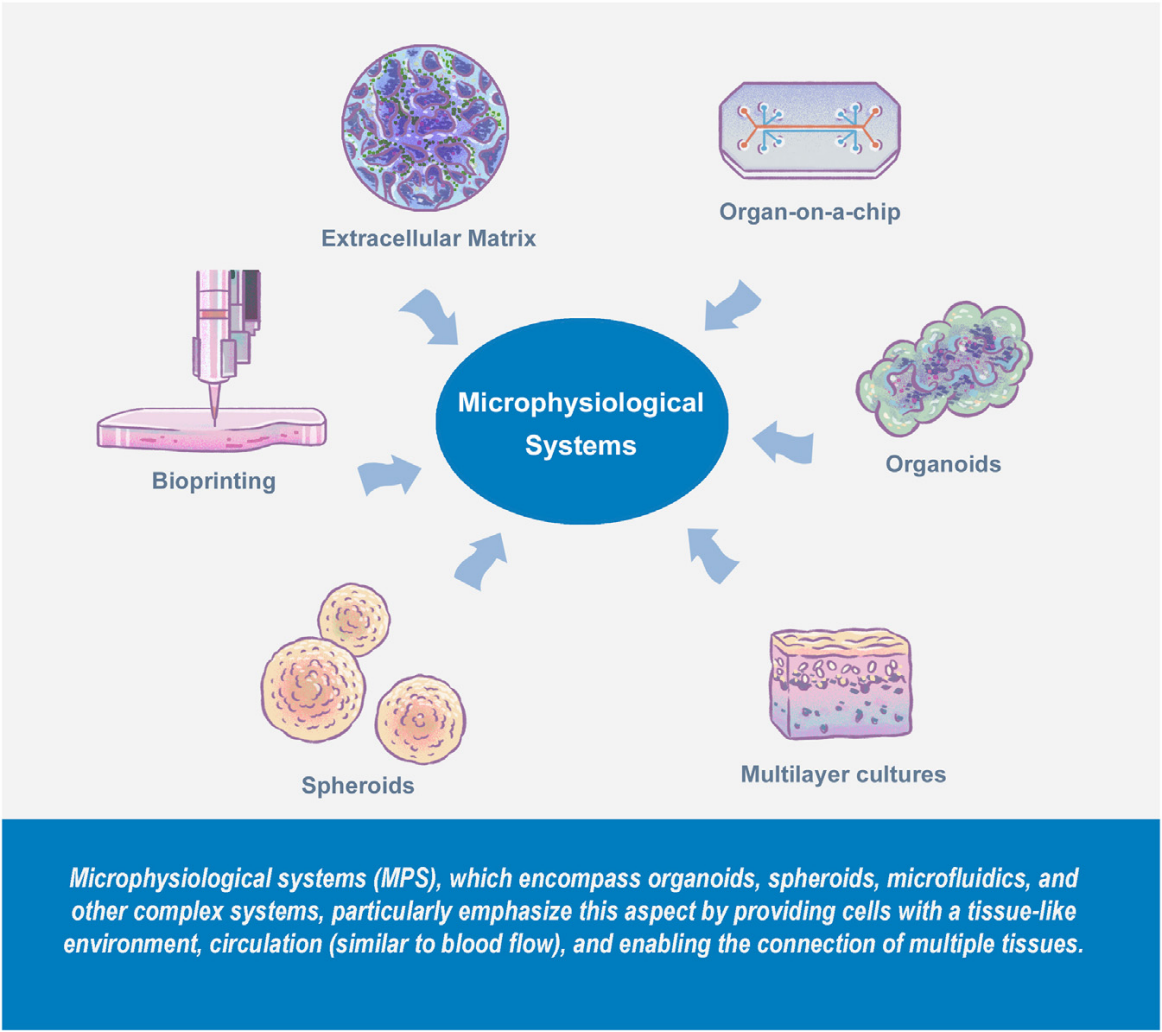
Examples and definition of MPS

The term MPS has been defined as “complex, multi-cellular *in vitro* systems that commonly include three-dimensional (3D) aspects, fluid flow, changing pressure or stretch, and multi-organ interactions” (NAS 2021). Key elements of these systems include co-cultures of different cell types, the use of scaffolds and extracellular matrices, or the incorporation of perfusion platforms. When MPSs involve microfabrication or microfluidics, they are often referred to as organ-on-chip (OoC) technologies (Figure 1). However, it is important to remember that models which do not include microfabrication, such as organoids, also fall under the definition of MPS. Their complexity brings advantages but also some limitations, which have been described elsewhere (Ekert et al., 2020; Pamies and Hartung 2017). To develop MPSs, an integrated interdisciplinary approach merging technologies and concepts from different disciplines is required, ranging from microfabrication, microfluidics, biomaterials, stem cell science, pharma-/toxicology, and medicine (Rogal et al., 2022). Therefore, new quality control (QC) and reporting standards are needed to ensure the performance and quality of cultures and the proper reporting of data and conclusions.

Numerous guidance documents are available for cell models. For instance, the Good Cell Culture Practice (GCCP) task force of EU research institutions issued an initial guideline in 2005 (Coecke et al., 2005). This document has recently been updated to consider new aspects, including MPSs (Eskes et al., 2017; Pamies et al. 2017a, 2018), toward GCCP 2.0 (Pamies et al. 2020, 2022). The revised document is aligned with the internationally recognized OECD guidance document on Good *in Vitro* Method Practice that is intended to support method developers and end users working to establish new *in vitro* assay methods in academic, industry, or government laboratories (GIVIMP 2018). Moreover, the “Recommended Guidelines for Developing, Qualifying, and Implementing Complex *In Vitro* Models (CIVMs) for Drug Discovery” has also been recently published (Ekert et al., 2020). This perspective article covers the various stages of early drug discovery and outlines key aspects that should be considered when developing, qualifying, and implementing MPSs (Ekert et al., 2020). In addition, quality criteria for *in vitro* human pluripotent stem cell (PSC)-derived models have been compiled (Pistollato et al., 2022). Other activities, like the one promoted by the International Society for Stem Cell Research, have focused on reporting standards for stem cells and their progeny (ISSCR 2023). For example, NC3Rs has also developed a "Reporting *In Vitro* Experiments Responsibly" guidance (NC3Rs 2010). Several other documents, such as “Toward Good *In Vitro* Reporting Standards” (Hartung et al., 2019) or those by the PRO-MaP initiative (PRO-MaP 2024), (sharing of detailed methods and reusable, step-by-step protocols in the life sciences) are of relevance to MPSs.

The increasing role of complex technology in the field of MPSs in particular and the need for standardization require more effort. For instance, the Joint Research Centre of the European Commission (EC) and the European Standardisation Organisations CEN-CENELEC organized a workshop titled “Putting Science into Standards” (Piergiovanni et al. 2021a, 2021b). These activities are now continued by a Focus Group, with the goal to develop a roadmap to steer future formal standardization activities by the CEN-CENELEC Technical Committees. Also, the American Society for Testing Materials (ASTM) issued the first technical standard on MPS, to clearly define and harmonize terminology in the field (ASTM). Other standardization initiatives have also focused on OoC, such as the "Workshop on Standards for Microphysiological Systems/Organ-Tissue on a Chip 2023" organized by the National Institute of Standards and Technology (NIST 2023), or the ISO draft ISO/DIS 226916 (ISO 2022a) for standards of OoC geometry, size, footprint, and interfaces. Furthermore, the geometry parameters for plastic and glass have been standardized by the ANSI/SLAS Microplate Standards to ensure compatibility with robotic systems, as these materials continue to be widely used by various companies.

## Specific challenges of QM for MPS

The present document considers two key issues. First, it provides some background on QM considerations for academic researchers, and second, it addresses the technical challenges of MPSs in comparison to conventional cell cultures.

Concerning issue 1, it is essential to note that MPS is an emerging field with a very dynamic development. In this situation, strict guidance, as applied in regulatory settings, may either not be appropriate or become obsolete within short time periods. Instead, it is important to define basic permanent principles underlying QM, so that each laboratory can set up its own fit-for-purpose rules and QC measures.

Concerning issue 2, it is often underappreciated that principles firmly established for conventional cell cultures and proved to be of high value also apply to MPSs. Some of these are therefore being recalled here. In addition, it has become clear that additional QM measures have to be considered for MPS aspects that are not found in conventional cultures. Typical examples are related to e.g., tissue architecture and systems mimicking the blood circulation.

Different MPS types, such as OoC and spheroids, present unique challenges. For instance, OoC models face issues like bubble formation in microfluidic channels, while spheroids, being simpler 3D cultures, have better reproducibility but may not provide the full complexity of tissues which can be obtained with organoids. Although organoids can replicate tissue anatomy, they face scalability and reproducibility challenges. QCs, like measuring glucose consumption or gene expression markers, can enhance reproducibility by indicating when culture parameters need to be regulated, or showing which MPS preparations need to be discarded. Simply using standards like ISO9001 and GMP will not control variability, which requires careful monitoring of critical culture attributes in derived cell cultures. A robust QC regime is essential, focusing on clear acceptance criteria (AC) so that only those cultures that meet quality criteria are used in experimental work, or that adjustments can be made to culture conditions to bring them within the tolerances for quality criteria. A key question is, “which quality standards should MPS meet”? The utilitarian approach suggests using the simplest effective test system. They should be fit for their intended purpose, whether studying a specific mechanism or assessing potential biological disruptions.

## QM for complex culture systems

### Why is “quality” important and how should it be applied in research programs

The “quality” of laboratory procedures is clearly important for the veracity of research outputs, particularly for complex cell culture systems, but how should we identify key actions to ensure that the quality of research data is acceptable? QM encompasses all aspects of the experimental work (e.g., equipment, materials, procedures, staff competencies) and is dependent on the research context of each project and laboratory (Pamies et al., 2022). QC, which involves test procedures implemented at various checkpoints, is essential to guarantee the reproducibility of each step in the production process and the consistent performance of individual experiments. The usual procedure is to set AC that QCs have to meet (Holzer et al., 2023). The QM plan also defines which QC needs to be performed at which time, in which frequency, and under which conditions. They also define the respective production or application stages. The more robust, reproducible and well-detailed the production steps are, the less QC will be necessary. The same applies to the robustness of experimental protocols (SOPs; standard operating procedures) and the need for control test runs. Thus, the key to delivering high-quality research data is a quantitative and comprehensive application of “principle 1” of GCCP (Pamies et al., 2022), i.e., “to understand the cell system you are working with, and what affects it”.

Key scientific quality parameters and provisional QC methods will emerge from the initial development of the model and system. Thorough characterization during the early development and optimization phase will drive the identification of suitable quality parameters, selection of test methodologies, and the acceptable variations in readouts. Definitions of important “base-line situations”, such as the running of negative and positive controls, are important for the setup of AC. It is important to have these approaches implemented formally within laboratory operation. The panel of considerations is broad (Table 1), and each system, or even each application of a given MPS, has its own particular requirements. It is clearly the direct responsibility of the laboratory head scientists (PIs) to establish appropriate QC procedures, as this impacts the quality of data produced and communicated to stakeholders, funding authorities, and other parties. The nomination of a quality coordinator to support the maintenance of the quality of laboratory outputs is also crucial, particularly to ensure that new staff are suitably inducted into the laboratory’s quality ethos and core quality and safety procedures.

**Table 1:** Exemplification of QM aspects

QC considerations^a^			Examples, comments
A	**MPS “systems setup”**		
	1	Cells as such	Identity, quality, differentiation
	2	Matrix and medium “as such”	Sterility; composition; stiffness, etc.
	3	Tissue (formed from 1&2)	Composition, 3D structure
	4	Technical device	Air bubbles, physical/construction parameters
	5	Basic environmental parameters	Temperature, Flow, CO_2_, pH
**B**	**MPS “experiments”**		
	1	Experimental design	Randomization; blinding
	2	Statistical design	Statistical unit; normalization reference; evaluation plan
	3	Acceptance criteria for valid “runs”	Positive and negative controls within specified limits
	4	System dynamics in line with historic controls	Baseline, noise, maximal signal
	5	Handling of test/reference items	Stocks, solvents, storage, etc.
**C**	**MPS “data recording”**		
	1	Sampling procedure	Invasive/non-invasive steps
	2	Sample processing	Storage; labeling; processing pipeline
	3	Analytical device(s)	Online/offline process; calibration process
	4	Baseline stability	Drift, handling effects
	5	Acceptance criteria for quantification procedure	Recovery; mass balance; internal standards
**D**	**MPS data processing and storage**		
	1	Data “cleaning”	Definition and handling of outliers
	2	Data processing framework	Normalization; curve fitting; endpoint combinations
	3	FAIR data	Repository, versioning, access conditions
	4	Meta data documentation	Association with data and with A–D
	5	Data reporting	Inclusion of information from A–E; transparent display
	6	Data – protocol alignment	Version management, subprotocols, deviations
**E**		**MPS applications**	**Physiology vs. pathophysiology vs. pharmacology**^b^
	1	Scaling issues	relative organ sizes, blood flow, oxygen supply, tissue-to-fluid ratio
	2	Functional endpoint(s)	Define AC; specify combination assessed; relevance
	3	Pathological endpoint(s)	Define AC for disease aspect or symptom(s) modeled
	4	Considerations of effect size	Statistically based, or biological rationale
	5	Reference treatment (pos. treatment control)	AC for range; relevance to test item
	6	Definition of treatment success	Information on endpoint(s) used; argument for relevance

a The list is exemplary in the sense that some points may not apply to all MPSs, while additional issues may be considered where applicable. Each issue in areas A–D is likely to be relevant for most MPSs. However, actions to be taken depend for each item on the purpose of the study and on other factors. Especially in academic settings, only some points will lead to formalized QC procedures, while others will be part of routine (non-formalized, non-quantified) checking of experiments. Some items apply to each MPS device and each test run, while others refer to the general setup and would thus be (re)-considered in larger time intervals.

b The items listed under E are meant to highlight that MPSs might be used for various types of biomedical or basic biological studies. Each field brings along its own specific requirements. For instance, studies on physiological regulation may need to particularly focus on scaling issues or the genetic background of cells, but there is no need to define particular damage states. Studies on pathophysiology need to consider the changes of the MPS due to the pathological processes modeled (e.g., loss of cells), in the field of toxicological pathology, also the distribution behavior of test chemicals would need considerations and possibly QC. For pharmacological studies, both, the diseases state to be reversed and the “cured” state that is desired may need definitions and possibly QC measures to allow calibration of the system.

For more detailed information, please see supplementary material 1*, section 4 extended version.*

### Establishing AC for the use of MPS

#### Different perspectives on AC

The reproducibility of scientific data is fundamentally dependent on the reliability of all included materials, cells, and reagents, as well as experimental procedures, analytical methods, and instruments. In order to define measures to manage the quality of test system, test method, and of a test method run, it is necessary to define them.

The test system describes the biological key properties of a culture type, e.g., a liver organoid, a cerebral organoid, or a muscle-nerve assembloid. It should include details of all physical (e.g., size, spatial cellular arrangement), chemical (e.g., matrix components, scaffold), and biological elements (e.g., percentages of different cells, cell source) (Holzer et al., 2023; Krebs et al., 2020). An MPS is, in most cases, a test system. One can set an AC for certain MPS specifications (e.g., organ size, cell differentiation state or certain functions). Notably, in a few cases, MPS may also be considered a test method, as detailed in the following section.

A test method is based on the use of the test system for a specific purpose, i.e., to test a hypothesis, such as determining under which conditions a certain treatment affects a certain organ, or a given pathological process. Example questions may include the following: how does the electrical activity of a brain MPS change in the presence of certain mutations, how does a virus replicate in a specific tissue type, or what is a compound’s effect on a certain organ. The test method includes several critical components: first, an exposure scheme, how and for how long the test system is exposed to the substance, including access to the test cells e.g., solubility, and whether repeat dose treatment is required; second, an endpoint definition, which specifies the parameters to be measured, such as electrical activity, apoptosis, hormone levels, or viral RNA content; third, a prediction model or data interpretation procedure, which uses endpoint data to accept or reject the hypothesis. If all these elements are defined for a certain MPS, it can be considered a test method. More detailed definitions can be found elsewhere (Collen et al., 2024; Leist et al., 2010).

QC may be applied to all these elements. Moreover, the overall test method can be evaluated for its readiness with respect to various uses. The most formalized readiness evaluation is called validation in several regulations and in the OECD guidance document 34 (GD34) on “validation and international acceptance of new test methods for hazard assessment”. Broader and more flexible approaches of readiness assessment are often described by the term “qualification.” In all cases, the goal is to provide information on the performance, robustness, and relevance of a test method. One particularly powerful but also highly resource-consuming element of QC of test methods is multi-center studies (so-called ring trials).

For example, in toxicological methods, it is important to assess the prediction model’s performance against some form of ground truth (i.e., absolute knowledge on what would be the correct outcome, i.e., effects of the toxicant on the human population). Often such absolute knowledge is not available, then the test method outcomes can be compared to a reference dataset considered to be largely correct (traditionally, such datasets have been the outcomes of animal studies). The definition of suitable positive and negative controls, and in addition, reference materials to monitor variation in assay performance over time or between laboratories, is an important element of the validation/qualification.

Alternative validation approaches (using processes and principles different from GD34) include mechanistic validation (Hartung et al., 2013). Such an approach may provide a measure of test method performance, even when a good reference dataset is not available (Leist et al., 2012; Patlewicz et al., 2013; Patterson et al., 2021; Tigges et al., 2021). As a complete formal validation of a test method is extremely resource intensive, it is important to define which information is really required for the specific application in question. This has resulted in the “fit-for-purpose” concept of test method qualification, which is particularly relevant for MPSs. In this field, each lab will typically perform a qualification of the respective MPS within its own quality system (Hulsemann et al., 2022). Also, periodic requalification may be needed if local test conditions change (e.g., reagents, equipment, cell source alterations).

For the continued use of a test method, it is important to note that any form of readiness evaluation is a one-time process (at least until a re-qualification takes place). It determines the validity/fit-for-purpose of a test method as such, but it cannot guarantee that each test run (i.e., each experiment using an MPS) yields reliable results. To assure the quality of test runs, so-called AC can be defined (Holzer et al., 2023).

**Figure:**
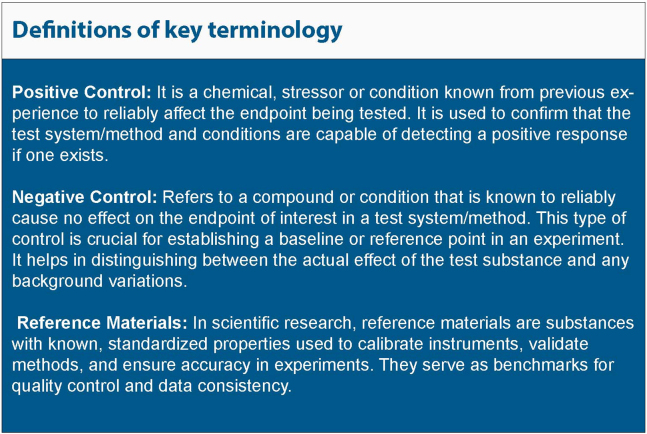

The test run is the process of using a test method in experiments (e.g., testing various drugs). Each run must meet “in-process AC” to ensure its validity. The most common AC are based on the data generated from positive/negative controls that run in parallel to unknown compounds/conditions (in every test run). AC predefine the outcome of control testing and they include an action plan if that outcome is not met (e.g., discarding data if a negative control fails). It is also useful to check the control data against “historical controls.” These are records of past test runs that help determine if a current test run’s results are typical and if there has been a drift in test results.

#### The fit-for-purpose concept

For the regulatory use of test methods, especially in the area of toxicology, it is mandatory to establish some form of “validity” for the method. A classical approach to validation establishes reliability, relevance, and predictability of the method, which is often very time-consuming (up to 10 years) and expensive. Moreover, predictivity is a difficult concept, when a test system addresses only a small aspect of a given pathology, tissue, or regulation circuit. Very extensive validation processes have been established for toxicity testing where questions are relatively simple (e.g., does a compound cause eye irritation?) and do not change over time. However, these pre-conditions do not apply to many (or most of) current applications of MPSs since the question or hypothesis tested will evolve with the research driven. Thus, there is a need for alternative validation concepts that focus on “fit-for-purpose.” This approach evaluates the method’s robustness and relevance for a narrowly defined objective. This is necessary and useful, as one given MPS may be used as the test system for various test methods; and test methods may be adapted to highly specific questions (e.g., a given liver MPS may be used to assess drug metabolism or the pharmacological effect of antiviral drugs). The concept of “fit-for-purpose” is particularly suited to validate complex methods as it focuses on the system’s functionality in a narrow context rather than defining each and every system feature in general.

Fit-for-purpose validations relieve the validation process of some heavy, resource-consuming loads, by assessing the system’s performance for a defined purpose. This relaxes not only the constraints of having to define predictivity, but also reliability and relevance, which are seen in a different light: they need to be sufficient only to answer the specific question evaluated. An alternative, or rather a complement to the fit-for-purpose concept, is to move away from a binary categorization of a test method as being validated or not validated. Instead, one has introduced the concept of “readiness.” As many readiness levels can be defined, a system can have a sufficient readiness for certain applications (e.g., screening and prioritization), but not for other applications (regulatory toxicology), and its “readiness level” can gradually change, as more information and more test data become available (Bal-Price et al., 2019).

#### Transition from formal assay validation to in-process validation

Formal validation is not only difficult to apply to MPSs (see previous section), but it also has the disadvantage of “freezing” the developmental state of a technology. A method that is formally validated cannot be changed and developed further without a need for a new validation. This is particularly problematic for an emerging, highly dynamic field, such as the MPS. It also applies to many areas of drug development, where questions may be specific to a given drug, or a specific program (within only one institution/company). In such cases, it has become common to shift the focus from a formal and broad validation of a test method to showing that all individual test runs are consistent, reliable, and anchored to the performance of meaningful control conditions. In short, the focus is shifted from validation to the use of comprehensive AC for each test run.

There are other areas where such approaches have become quite common, as in the testing of epidemiological hypotheses, or in social and political science studies. In many fields, the test method used to study a hypothesis cannot be extensively validated prior to the study. In such cases, there is a high demand to run many layers of consistency controls within the study. For example, in drug testing on humans, not only positive (competitor drug) and negative (placebo) controls are run, but also historical records on control vs*.* disease populations are considered. Inclusion and exclusion criteria are strictly defined and closely monitored, and many additional data on secondary and tertiary endpoints are acquired to control whether the trial is consistent within itself and with all general medical knowledge.

If one would apply this approach to MPS-based test systems, they may be validated only to a minimal extent (e.g., for the reliability/quality of the cells/MPS used, and for producing robust baseline data). Instead, each test run would be accompanied by a well-chosen and comprehensive set of AC. If all control compounds and control conditions are within the limits set for AC, this may be considered as an “in-process validation”.

## Cell source QC

The selection of a cell source and the establishment of QC for the cells is the basis of setting up an MPS. Misidentification or other early technical errors need to be avoided. A detailed description of the cells (e.g., how they were created, species, origin, special features, karyotype, gender, health state, and age) is important and needs to be well documented and controlled. Documentation of QC on contaminants such as mycoplasma, human viruses, fungi, and other pathogens is very important (GIVIMP 2018; Pamies et al., 2022; Pistollato et al., 2022). Some studies have shown that misidentification and mycoplasma contamination are still common issues (Horbach and Halffman, 2017; Huang et al., 2017; Olarerin-George and Hogenesch, 2015; Timenetsky et al., 2006). Some of these QCs have to be performed at regular intervals, aligned with the local use, storage, and banking/maintenance of the cells (Pamies et al., 2022). An often underestimated issue relates to ethical concerns pertaining to donors, specifically in terms of obtaining consent and ensuring the privacy of donor information. In this context, the utilization of human PSC lines for MPSs presents a significant advantage over using primary samples, as it requires donor consent and cell banking only once, allowing for unlimited use of the same cell source. Additionally, induced PSCs (iPSCs) facilitate increased standardization by providing cells with a consistent genetic background for experimental work (Kuse and Taniguchi 2019). Moreover, for iPSCs intended for use in EC-funded research, QC information must be submitted for publication on the hPSCreg database (www.hpscreg.eu). For ongoing expert discussions on human PSCs, please visit www.iscbi.org. The European Bank for induced Pluripotent Stem Cells has recently published an outline for the establishment and implementation of a QC regime suitable for a large-scale operational setting (O'Shea et al., 2020). These recommendations are intended for academic applications. In addition, detailed quality criteria for *in vitro* human iPSC-derived models have been recently published (Pistollato et al., 2022) and an ISO standard (ISO 24603) for both human and mouse PSCs for research use has also been published (ISO 2022b). This document contains a list of generic minimum QC tests and examples of helpful methodologies and AC for human stem cell selection. Furthermore, some of the most common and relevant morphological, biochemical, and functional endpoints that can be used as AC for iPSC-derived models have been discussed (Pistollato et al., 2022).

Alternative to iPSC, primary cells can also be utilized in developing MPSs and offer certain benefits (e.g., the intellectual property of iPSC technology comes with some commercial risk). Detailed quality specifications for cell types can be found in other sources (Geraghty et al., 2014; Pamies et al., 2022).

## Quality parameters and control for research data from OoC systems

### Materials and microfluidics

In this section, we will outline the quality parameters and control considerations specific to OoC technologies. Although OoC technologies form a subset of MPSs, they possess unique aspects that require special attention. The choice of material used for chip production is usually driven not only by careful considerations, balancing ease of manufacturing and functionality for the biological model, but also biocompatibility, possibility to physically access the cell culture and obtain accurate readouts. Within the chip structure, the cell model may be cultured within a scaffold (using commercial or *ad hoc* inserts and/or materials that mimic the extracellular matrix), to maintain the proper 3D structure, promote cell growth, and contribute to the tissue-specific characteristics. Hydrogels, either natural or synthetic, are a very common choice, due to their soft mechanical properties and biocompatibility. Commercial products and protocols for hydrogel creation are widely available, but there is a lack of tissue specificity, making it difficult to conclude on their relevance for the application. Animal-derived matrices (e.g., Matrigel, Geltrex) have been a popular choice for different kinds of cell models, but their biological variability is a source of unreliability in the experimental outputs. Some synthetic, animal-free alternatives are now available (Aisenbrey and Murphy 2020), and the field of generating them is highly dynamic.

Regarding the chip’s main structure, the traditional choice is poly(dimethylsiloxane) (PDMS) (Huh et al., 2010), a silicone rubber that is easily fabricated using lithographic design templates. This choice provides flexibility in the design, oxygen permeability, optical transparency, and biocompatibility. Since many endpoints are evaluated through microscopy (Rusyn et al., 2022), PDMS is usually bonded via plasma activation on a glass slide, an inert material with optimal optical characteristics. A well-known issue of PDMS application for OoCs is the absorption of small hydrophobic molecules in the bulk material (Auner et al., 2019; van Meer et al., 2017), and the impact of PDMS monomer on the cell culture is under discussion (Regehr et al., 2009). More recently, developers are increasingly starting to produce their chips from thermoplastic materials, already used in other biotechnological applications: polystyrene (Lee et al., 2018), poly(methyl methacrylate) (Busek et al., 2021), polycarbonate (Wagner et al., 2013), and cyclic olefin copolymer. While being amenable for cheaper and more scalable fabrication processes as well as providing significantly lower absorption issues, thermoplastic materials are limited in terms of oxygen permeability and elastic properties. Some recent reviews explain in detail the main considerations on how to choose the best materials for an OoC (Leung; Low et al., 2021).

Although the complexity of OoC devices (and their reliability and reproducibility) heavily resides with the biological component (Rusyn et al., 2022), there is also guidance for scale-up and control of the manufacturing processes (Hinman et al., 2020; Leung et al., 2022; Piergiovanni et al., 2021b). It is crucial that SOPs are carefully drafted, revised, and followed during both the design phase and the fabrication process. To ensure the reproducibility of the fabrication process, some QC checkpoints should be established and routinely used, to ensure that both the product components and the final assembly comply with the pre-defined specifications. These can include checklists to verify dimensions, tolerances, device geometrical and functional characteristics, quantification of small molecule binding to the materials used, etc. Additionally, QC on surface functionalization, sensor calibration and sensitivity, and the functionality of mechanical actuators have to be considered. Instructions on how to document the QC performed should be part of the SOPs. Examples of common QC are listed in Table 2. Particular care should be given to the leak tightness and the absence of bubbles since these are the main failure causes of microfluidic systems (Leung et al., 2022; Wang et al., 2012). A good summary of possible test methods for leak testing is available (Silverio et al., 2022). At a higher level of production, for commercialized devices, it is recommended that the manufacturer follows GMP to ensure a reproducible product. GMP guidelines (EU 2015; FDA 2011; WHO 2021) also ensure that materials used have a desired batch-to-batch reproducibility. They ensure that all equipment used in production is correctly verified (calibrated and maintained), that the process is validated, and the personnel are adequately trained. Preventive and corrective changes in the corresponding SOPs based on the analysis of the nonconformities lead to a continuous improvement in quality. A documentation of the relevant process parameters, batch numbers, operator, etc., makes it much easier and more objective to identify the cause of nonconformities. After installation and delivery to the customer or research partner, installation qualification, operational qualification, and performance qualification, validation/qualification protocols are helpful and should be implemented, together with a complete and thorough user manual. For the devices that include assembly by the user, QC criteria that the user should perform routinely should also be included.

**Table 2:** Common quality controls

Quality Control(s)	Measurement(s)
Bubbles – Absence of bubbles inside microfluidics and chip material	Optical inspection Integrated bubble sensor
Sterility – QC for sterilization process	General: ISO 14937:2009 Steam sterilization: ISO 17665-1:2006 Ethylene oxide: ISO 11135:2014 Formaldehyde: ISO 25424:2018
Cleanliness – absence of particles/dust	Optical inspection
Dimensions and Alignment – geometric dimensions of microfluidics and interfaces between parts inside tolerance	Light microscopy Electron microscopy Atomic force microscopy Optical profilometry
Sensors – calibration of integrated sensors	According to manufacturer recommendations
Flow – volume flow inside tolerances and channels not clogged	Optical inspection Integrated flow sensor for liquids with particles: micro-particle image velocimetry (Lima et al., 2008) or Doppler optical coherence tomography (Carrion et al., 2009)
Leak tightness – all channels, tubes, etc. Are gas/liquid tight	(Silverio et al., 2022): Pressure change method Bubble emission method Tracer gas method
Electrical insulation – no short circuits between electrodes or electrical connections	Electrical resistance between electrodes

### Associated devices and automation

The devices that are part of the assay or experiment are a key part of the cell culture system and should be part of the developed QM plan. They include e.g., pumps, pipettes, sensors for online measurements, and others. This wide field is beyond the scope of this short review, but we feel obliged to highlight its importance. In addition, it is necessary to assess if calibration routines and QC measures need to be implemented for the devices utilized in the intended application of the test system. The necessary extent and frequency of calibrations and QC are derived from the reliability of the instrument used and the relevance to the assay, as described previously. A good starting point for both is provided by the manufacturer’s specifications and recommendations. For home-developed devices, there are two good points of reference: firstly, it can be derived from the process on how precise the parameter under consideration must be; secondly, specific standards, like ISO 8655:2022 for pipettes, provide a good indication of the required and technically feasible accuracy.

Notably, each additional calibration steps and QC make the QM process more complex. Therefore, the additional effort should be evaluated against the effort of process redesign to reduce the calibration steps and amount of QC early on. One common approach is inclusion of additional sensors and closed loop controllers to the process. This allows the process to be monitored more closely and enables a defined and stable cell culture environment. Easy to implement examples are the control of temperature, gas concentrations, pH, and volume flow (Kilic et al., 2018). The next step, to further increase process reliability and scalability, is to automate or mechanize the process partially or completely. It is important to note that the processes can only be efficiently transferred to a robotic system if the process itself is optimized from a “for human-made” process to an automated machine-based process. This also means that the QM plan and the associated calibration routines and QC must be adapted.

## QC for different organ systems

**Figure 2:**
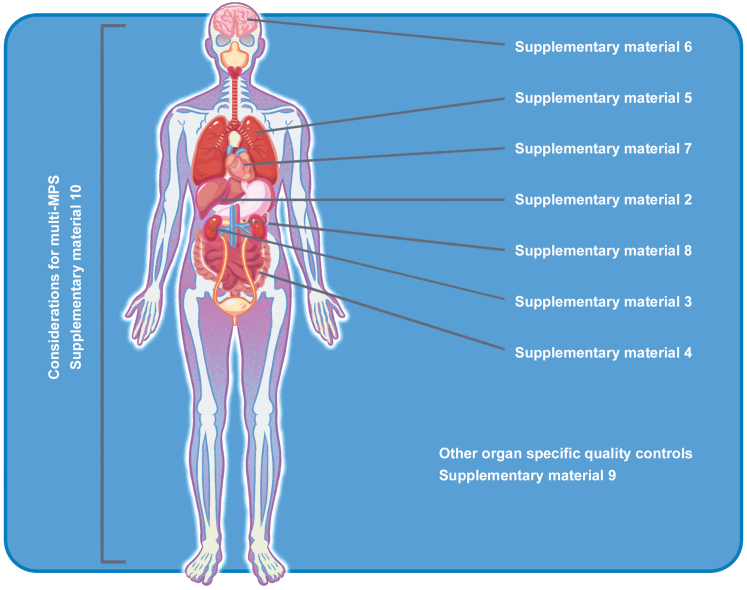
Schematic overview of organ-specific QC aspects for MPSs Guidance is given on the respective supplementary material document to be consulted for more specific information.

Each MPS has specific QM issues related to the organ or tissue being modeled. For example, the heart requires specific types of cells and functional readouts that differ from all other organs. As the number of tissue-specific MPSs (and their combinations on multi-organ chips) is overwhelming, we selected some representative examples (i.e., liver, kidney, gut, lung, brain, heart, pancreas) to provide an overview of the possible QC to be considered (Figure 2). The assays indicated by the authors in the referenced supplementary information are an assessment of current methodologies. It is anticipated that new methods and technologies will emerge quite rapidly and the assays indicated in the supplementary information will need to be adapted and replaced as scientific knowledge develops.

## QM during adaptation of MPS for different readouts

The 3D cultures used in most MPSs promote the formation of cell-cell connections and may generate even organotypic structures. Microfluidics and other on-chip technologies further improve some physiologically relevant conditions. The increased model complexity is associated with challenges to obtain readouts from the MPSs. For instance, immunostaining is more complicated than in conventional 2D cultures; and imaging of stained 3D cultures poses additional problems. It could be difficult to use these readouts for QC. Specialized microscopy techniques and tissue processing (e.g., clearing) can overcome these challenges, but they require additional specifications and QC (Nurnberg et al., 2020; Renner et al., 2021; Cheng et al., 2023; Yoshida et al., 2020; Kahn-Krell et al., 2022).

Another example is the measurement of electrical activity. Technologies to adapt established 2D technologies to 3D are emerging (Pamies et al., 2017b; Sandstrom et al., 2017; van Vliet et al., 2007; Huang et al., 2022; Huang et al. 2017, 2022; Kalmykov et al., 2019). These two examples indicate the wide range of readout adaptations to be considered. In many cases, difficulties in scale with the size and dimensionality of the system to be assessed and technologies known to be reliable and with high signal-noise ratios in 2D may provide large challenges in 3D. Sometimes, new types of control parameters may need to be introduced to establish relevant AC.

## Conclusions

CIVMs, jointly referred to as MPSs, aim to represent higher-level anatomical and physiological models of human biology. However, the adoption of MPSs by the pharmaceutical, chemical, cosmetic, and food industries is still moderate at best. This is not only due to the novelty of these methodological approaches and the limited experience and expertise in many institutions. This latter issue can be overcome by the design, use and documentation of reliable QM plans, and the publication of such information along with the data.

The growing interest in MPS technologies is evident through their exponential uptake across diverse fields, exemplified by the formation of organizations like the International MPS Society and the European Organ-on-Chip Society (EUROoCS), along with events like the Microphysiological Systems World Summits. These platforms reflect the collective commitment to harnessing the capabilities of MPS to not only offer ethical alternatives to animal testing, but also to advance scientific discoveries and testing methodologies.

To fulfill this transformative potential, it is imperative for all stakeholders, including academic communities, to ensure that user communities increase their confidence in these emerging life science tools. This manuscript offers practical insights into QM strategies for MPSs, making them applicable in academic settings as well. In doing so, we bridge the gap between innovation and trust, thereby ensuring that MPS continue to shape the future of *in vitro* research and applications.

## Supplemental information

Document S1. Supplementary material S1–S10 and Tables S1–S9

Document S2. Article plus supplemental information

## References

[bib1] Aisenbrey E.A., Murphy W.L. (2020). Synthetic alternatives to Matrigel. Nat. Rev. Mater..

[bib2] ASTM Standard Terminology Relating to Microphysiological Systems. In.https://www.astm.org/f3570-22.html Accessed 21/02/2024 2024.

[bib3] Auner A.W., Tasneem K.M., Markov D.A., McCawley L.J., Hutson M.S. (2019). Chemical-PDMS binding kinetics and implications for bioavailability in microfluidic devices. Lab Chip.

[bib4] Bal-Price A., Hogberg H.T., Crofton K.M., Daneshian M., FitzGerald R.E., Fritsche E., Heinonen T., Hougaard Bennekou S., Klima S., Piersma A.H. (2019). Recommendation on Test Readiness Criteria for New Approach Methods in Toxicology: Exemplified for Developmental Neurotoxicity (vol 35, pg 306, 2018). ALTEX.

[bib5] Busek M., Novik S., Aizenshtadt A., Amirola-Martinez M., Combriat T., Grünzner S., Krauss S. (2021). Thermoplastic Elastomer (TPE)-Poly(Methyl Methacrylate) (PMMA) Hybrid Devices for Active Pumping PDMS-Free Organ-on-a-Chip Systems. Biosensors-Basel.

[bib6] Carrion L., Hamel E., Leblanc-Hotte A., Boudoux C., Guenat O., Maciejko R. (2009). Characterization of microfluidic systems with Doppler optical coherence tomography. Proc. SPIE.

[bib7] Cheng W., Zhou Y., Xie Y., Li Y., Zhou R., Wang H., Feng Y., Wang Y. (2023). Combined effect of polystyrene microplastics and bisphenol A on the human embryonic stem cells-derived liver organoids: The hepatotoxicity and lipid accumulation. Sci. Total Environ..

[bib8] Coecke S., Balls M., Bowe G., Davis J., Gstraunthaler G., Hartung T., Hay R., Merten O.W., Price A., Schechtman L. (2005). Guidance on Good Cell Culture Practice - A report of the second ECVAM task force on Good Cell Culture Practice. Altern. Lab. Anim..

[bib9] Cöllen E., Tanaskov Y., Holzer A.K., Dipalo M., Schäfer J., Kraushaar U., Leist M. (2024). Elements and development processes for test methods in toxicology and human health-relevant life science research. ALTEX.

[bib10] Ekert J.E., Deakyne J., Pribul-Allen P., Terry R., Schofield C., Jeong C.G., Storey J., Mohamet L., Francis J., Naidoo A. (2020). Recommended Guidelines for Developing, Qualifying, and Implementing Complex In Vitro Models (CIVMs) for Drug Discovery. SLAS Discov..

[bib11] Eskes C., Boström A.C., Bowe G., Coecke S., Hartung T., Hendriks G., Pamies D., Piton A., Rovida C. (2017). Good cell culture practices & *in vitro* toxicology. Toxicol. Vitro.

[bib12] EU (2015).

[bib13] EUR-Lex (2001). Directive 2001/83/EC of the European Parliament and of the Council of 6 November 2001 on the Community code relating to medicinal products for human use. http://data.europa.eu/eli/dir/2001/83/oj.

[bib14] EUR-Lex (2001). Regulation (EU) 2019/6 of the European Parliament and of the Council of 11 December 2018 on veterinary medicinal products and repealing Directive 2001/82/EC (Text with EEA relevance). http://data.europa.eu/eli/reg/2019/6/oj.

[bib15] EUROoCS In. https://eurooc.eu.

[bib16] FDA (2011).

[bib17] FDA (2021). FDA Modernization Act of 2021. https://trackbill.com/bill/us-congress-senate-bill-2952-fda-modernization-act-of-2021/2145312/.

[bib18] Geraghty R.J., Capes-Davis A., Davis J.M., Downward J., Freshney R.I., Knezevic I., Lovell-Badge R., Masters J.R.W., Meredith J., Stacey G.N. (2014). Guidelines for the use of cell lines in biomedical research. Br. J. Cancer.

[bib19] GIVIMP O. (2018).

[bib20] Hartung T., de Vries R., Hoffmann S., Hogberg H.T., Smirnova L., Tsaioun K., Whaley P., Leist M. (2019). Toward Good In Vitro Reporting Standards. ALTEX.

[bib21] Hartung T., Hoffmann S., Stephens M. (2013). Mechanistic validation. ALTEX.

[bib22] Hinman S.S., Kim R., Wang Y., Phillips K.S., Attayek P.J., Allbritton N.L. (2020). Microphysiological system design: simplicity is elegance. Curr. Opin. Biomed. Eng..

[bib23] Holzer A.K., Dreser N., Pallocca G., Mangerich A., Stacey G., Dipalo M., Van de Water B., Rovida C., Wirtz P.H., Van Vugt B. (2023). Acceptance criteria for new approach methods in toxicology and human health-relevant life science research - part I. ALTEX.

[bib24] Horbach S.P.J.M., Halffman W. (2017). The ghosts of HeLa: How cell line misidentification contaminates the scientific literature. PLoS One.

[bib25] Huang Q., Tang B., Romero J.C., Yang Y., Elsayed S.K., Pahapale G., Lee T.J., Morales Pantoja I.E., Han F., Berlinicke C. (2022). Shell microelectrode arrays (MEAs) for brain organoids. Sci. Adv..

[bib26] Huang Y., Liu Y., Zheng C., Shen C. (2017). Investigation of Cross-Contamination and Misidentification of 278 Widely Used Tumor Cell Lines. PLoS One.

[bib27] Huh D., Matthews B.D., Mammoto A., Montoya-Zavala M., Hsin H.Y., Ingber D.E. (2010). Reconstituting Organ-Level Lung Functions on a Chip. Science.

[bib28] Hülsemann M., Wiebach J., Drude N.I., Kniffert S., Behm L., Hönzke K., Baumgardt M., Hippenstiel S., Hocke A.C., Dirnagl U., Tölch U. (2022). Introducing quality measures in an academic research consortium: Lessons and recommendation from implementing an ad hoc quality management system for organ model research: Lessons and recommendation from implementing an ad hoc quality management system for organ model research. EMBO Rep..

[bib29] ISO (2022). ISO 22916:2022 Microfluidic devices. Interoperability requirements for dimensions, connections and initial device classification. https://www.iso.org/standard/74157.html.

[bib30] ISO (2022). ISO 24603:2022(en) Biotechnology — Biobanking — Requirements for human and mouse pluripotent stem cells. https://www.iso.org/obp/ui/#iso:std:iso:24603:ed-1:v1:en.

[bib31] ISSCR (2023). Basic and preclinical standards. https://www.isscr.org/standards-document.

[bib32] Kahn-Krell A., Pretorius D., Guragain B., Lou X., Wei Y., Zhang J., Qiao A., Nakada Y., Kamp T.J., Ye L., Zhang J. (2022). A three-dimensional culture system for generating cardiac spheroids composed of cardiomyocytes, endothelial cells, smooth-muscle cells, and cardiac fibroblasts derived from human induced-pluripotent stem cells. Front. Bioeng. Biotechnol..

[bib33] Kalmykov A., Huang C., Bliley J., Shiwarski D., Tashman J., Abdullah A., Rastogi S.K., Shukla S., Mataev E., Feinberg A.W. (2019). Organ-on-e-chip: Three-dimensional self-rolled biosensor array for electrical interrogations of human electrogenic spheroids. Sci. Adv..

[bib34] Kilic T., Navaee F., Stradolini F., Renaud P., Carrara S. (2018). Organs-on-chip monitoring: sensors and other strategies. Microphysiological Syst..

[bib35] Krebs A., Waldmann T., Wilks M.F., Van Vugt-Lussenburg B.M.A., Van der Burg B., Terron A., Steger-Hartmann T., Ruegg J., Rovida C., Pedersen E. (2020). Erratum to Template for the description of cell-based toxicological test methods to allow evaluation and regulatory use of the data. ALTEX.

[bib36] Kuse Y., Taniguchi H. (2019). Present and Future Perspectives of Using Human-Induced Pluripotent Stem Cells and Organoid Against Liver Failure. Cell Transplant..

[bib37] Lee Y., Choi J.W., Yu J., Park D., Ha J., Son K., Lee S., Chung M., Kim H.Y., Jeon N.L. (2018). Microfluidics within a well: an injection-molded plastic array 3D culture platform. Lab Chip.

[bib38] Leist M., Efremova L., Karreman C. (2010). Food for thought . considerations and guidelines for basic test method descriptions in toxicology. ALTEX.

[bib39] Leist M., Hasiwa N., Daneshian M., Hartung T. (2012). Validation and quality control of replacement alternatives - current status and future challenges. Toxicol. Res..

[bib40] Leung C.M., de Haan P., Ronaldson-Bouchard K., Kim G.A., Ko J., Rho H.S., Chen Z., Habibovic P., Jeon N.L., Takayama S. (2022). A guide to the organ-on-a-chip. Nat. Rev. Methods Primers.

[bib41] Lima R., Wada S., Tanaka S., Takeda M., Ishikawa T., Tsubota K.i., Imai Y., Yamaguchi T. (2008). In vitro blood flow in a rectangular PDMS microchannel: experimental observations using a confocal micro-PIV system. Biomed. Microdevices.

[bib42] Low L.A., Mummery C., Berridge B.R., Austin C.P., Tagle D.A. (2021). Organs-on-chips: into the next decade. Nat. Rev. Drug Discov..

[bib43] NAS (2021). Proceedings of a Workshop-in Brief.

[bib44] NC3Rs (2010). The ARRIVE guidelines (Animal Research: Reporting of In Vivo Experiments). https://arriveguidelines.org/.

[bib45] NIST (2023). Workshop on Standards for Microphysiological Systems/Organ-Tissue on a Chip. https://www.nist.gov/pml/microsystems-and-nanotechnology-division/biophysical-and-biomedical-measurement-group/organ.

[bib46] Nürnberg E., Vitacolonna M., Klicks J., von Molitor E., Cesetti T., Keller F., Bruch R., Ertongur-Fauth T., Riedel K., Scholz P. (2020). Routine Optical Clearing of 3D-Cell Cultures: Simplicity Forward. Front. Mol. Biosci..

[bib47] O'Shea O., Steeg R., Chapman C., Mackintosh P., Stacey G.N. (2020). Development and implementation of large-scale quality control for the European bank for induced Pluripotent Stem Cells. Stem Cell Res..

[bib48] OECD (2003).

[bib77] Olarerin-George A.O., Hogenesch J.B. (2015). Assessing the prevalence of mycoplasma contamination in cell culture via a survey of NCBI's RNA-seq archive. Nucleic Acids Res..

[bib49] Pamies D., Bal-Price A., Chesné C., Coecke S., Dinnyes A., Eskes C., Grillari R., Gstraunthaler G., Hartung T., Jennings P. (2018). Advanced Good Cell Culture Practice for Human Primary, Stem Cell-Derived and Organoid Models as well as Microphysiological Systems. ALTEX.

[bib50] Pamies D., Bal-Price A., Simeonov A., Tagle D., Allen D., Gerhold D., Yin D., Pistollato F., Inutsuka T., Sullivan K. (2017). Good Cell Culture Practice for Stem Cells and Stem-Cell-Derived Models. ALTEX.

[bib51] Pamies D., Barreras P., Block K., Makri G., Kumar A., Wiersma D., Smirnova L., Zang C., Bressler J., Christian K.M. (2017). A human brain microphysiological system derived from induced pluripotent stem cells to study neurological diseases and toxicity. ALTEX.

[bib52] Pamies D., Hartung T. (2017). 21st Century Cell Culture for 21st Century Toxicology. Chem. Res. Toxicol..

[bib53] Pamies D., Leist M., Coecke S., Bowe G., Allen D., Gstraunthaler G., Bal-Price A., Pistollato F., DeVries R., Hartung T., Stacey G. (2020). Good Cell and Tissue Culture Practice 2.0 (GCCP 2.0) - Draft for Stakeholder Discussion and Call for Action. ALTEX.

[bib54] Pamies D., Leist M., Coecke S., Bowe G., Allen D.G., Gstraunthaler G., Bal-Price A., Pistollato F., de Vries R.B.M., Hogberg H.T. (2022). Guidance document on Good Cell and Tissue Culture Practice 2.0 (GCCP 2.0). ALTEX.

[bib55] Parvatam S., Pamies D., Pistollato F., Beken S., Mariappan I., Roth A., Piergiovanni M., G C Maisonneuve B., Ewart L., Majumder A. (2024). Taking the leap toward human-specific nonanimal methodologies: The need for harmonizing global policies for microphysiological systems. Stem Cell Rep..

[bib56] Patlewicz G., Simon T., Goyak K., Phillips R.D., Rowlands J.C., Seidel S.D., Becker R.A. (2013). Use and validation of HT/HC assays to support 21st century toxicity evaluations. Regul. Toxicol. Pharmacol..

[bib57] Patterson E.A., Whelan M.P., Worth A.P. (2021). The role of validation in establishing the scientific credibility of predictive toxicology approaches intended for regulatory application. Comput. Toxicol..

[bib58] Piergiovanni M., Cangar O., Leite S.B., Mian L., Jenet A., Corvi R., Whelan M., Taucer F., Ganesh A. (2021). Putting Science into Standards workshop on standards for organ-on-chip. Stem Cell Rep..

[bib59] Piergiovanni M., Leite S.B., Corvi R., Whelan M. (2021). Standardisation needs for organ on chip devices. Lab Chip.

[bib60] Pistollato F., Bal-Price A., Coecke S., Parvatam S., Pamies D., Czysz K., Hao J., Kee K., Teo A.K.K., Niu S. (2022). Quality criteria for *in vitro* human pluripotent stem cell-derived models of tissue-based cells. Reprod. Toxicol..

[bib61] PRO-MaP (2024). Promoting Reusable and Open Methods and Protocols (PRO-MaP): Recommendations to improve methodological clarity in life sciences publications. https://www.bihealth.org/en/translation/innovation-enabler/quest-center/projects/project/pro-map.

[bib62] Regehr K.J., Domenech M., Koepsel J.T., Carver K.C., Ellison-Zelski S.J., Murphy W.L., Schuler L.A., Alarid E.T., Beebe D.J. (2009). Biological implications of polydimethylsiloxane-based microfluidic cell culture. Lab Chip.

[bib63] Renner H., Otto M., Grabos M., Schöler H.R., Bruder J.M. (2021). Fluorescence-based Single-cell Analysis of Whole-mount-stained and Cleared Microtissues and Organoids for High Throughput Screening. Bio. Protoc..

[bib64] Rogal J., Schlünder K., Loskill P. (2022). Developer's Guide to an Organ-on-Chip Model. ACS Biomater. Sci. Eng..

[bib65] Roth A., MPS-WS Berlin 2019 (2021). Human microphysiological systems for drug development. Science.

[bib66] Rusyn I., Sakolish C., Kato Y., Stephan C., Vergara L., Hewitt P., Bhaskaran V., Davis M., Hardwick R.N., Ferguson S.S. (2022). Microphysiological Systems Evaluation: Experience of TEX-VAL Tissue Chip Testing Consortium. Toxicol. Sci..

[bib67] Sandström J., Eggermann E., Charvet I., Roux A., Toni N., Greggio C., Broyer A., Monnet-Tschudi F., Stoppini L. (2017). Development and characterization of a human embryonic stem cell-derived 3D neural tissue model for neurotoxicity testing. Toxicol. Vitro.

[bib68] Silverio V., Guha S., Keiser A., Natu R., Reyes D.R., van Heeren H., Verplanck N., Herbertson L.H. (2022). Overcoming technological barriers in microfluidics: Leakage testing. Front. Bioeng. Biotechnol..

[bib69] Tigges J., Bielec K., Brockerhoff G., Hildebrandt B., Hübenthal U., Kapr J., Koch K., Teichweyde N., Wieczorek D., Rossi A., Fritsche E. (2021). Academic Application of Good Cell Culture Practice for Induced Pluripotent Stem Cells. ALTEX.

[bib70] Timenetsky J., Santos L.M., Buzinhani M., Mettifogo E. (2006). Detection of multiple mycoplasma infection in cell cultures by PCR. Braz. J. Med. Biol. Res..

[bib71] van Meer B.J., de Vries H., Firth K.S.A., van Weerd J., Tertoolen L.G.J., Karperien H.B.J., Jonkheijm P., Denning C., IJzerman A.P., Mummery C.L. (2017). Small molecule absorption by PDMS in the context of drug response bioassays. Biochem. Biophys. Res. Commun..

[bib72] van Vliet E., Stoppini L., Balestrino M., Eskes C., Griesinger C., Sobanski T., Whelan M., Hartung T., Coecke S. (2007). Electrophysiological recording of re-aggregating brain cell cultures on multi-electrode arrays to detect acute neurotoxic effects. Neurotoxicology.

[bib73] Wagner I., Materne E.M., Brincker S., Süssbier U., Frädrich C., Busek M., Sonntag F., Sakharov D.A., Trushkin E.V., Tonevitsky A.G. (2013). A dynamic multi-organ-chip for long-term cultivation and substance testing proven by 3D human liver and skin tissue co-culture. Lab Chip.

[bib74] Wang Y., Lee D., Zhang L., Jeon H., Mendoza-Elias J.E., Harvat T.A., Hassan S.Z., Zhou A., Eddington D.T., Oberholzer J. (2012). Systematic prevention of bubble formation and accumulation for long-term culture of pancreatic islet cells in microfluidic device. Biomed. Microdevices.

[bib75] WHO (2021).

[bib76] Yoshida S., Miwa H., Kawachi T., Kume S., Takahashi K. (2020). Generation of intestinal organoids derived from human pluripotent stem cells for drug testing. Sci. Rep..

