# Supplementary material for: Recommendations on fit-for-purpose criteria to establish quality management for microphysiological systems and for monitoring their reproducibility
Source: Stem Cell Reports. 2024 Apr 25;19(5):604–17. doi: 10.1016/j.stemcr.2024.03.009 (PMC11103889; doi:10.1016/j.stemcr.2024.03.009)
Supplement: Document S1. Supplementary material S1–S10 and Tables S1–S9 [file mmc1.pdf]

Stem Cell Reports, Volume *19*

Supplemental Information

Recommendations on fit-for-purpose criteria to establish quality management for microphysiological systems and for monitoring their reproducibility

David Pamies, Jason Ekert, Marie-Gabrielle Zurich, Olivier Frey, Sophie Werner, Monica Piergiovanni, Benjamin S. Freedman, Adrian Kee Keong Teo, Hendrik Erfurth, Darwin R. Reyes, Peter Loskill, Pelin Candarlioglu, Laura Suter-Dick, Shan Wang, Thomas Hartung, Sandra Coecke, Glyn N. Stacey, Beren Atac Wagegg, Eva-Maria Dehne, Francesca Pistollato, and Marcel Leist

Supplementary Material 1

Quality management (QM) for complex culture systems

Introduction to the QM and QC concepts, including acceptance controls (AC)

Quality management (QM) has been recently defined in the new GCCP guidelines as *"…a generic term that comprises measures and procedures implemented to provide consistent quality of cell cultures and to detect potential mistakes and errors*"(Pamies et al. 2022). QM encompasses all aspects of the cell cultures (e.g., equipment, materials, procedures, staff competencies) and it is dependent on the research context of each project and laboratory (Pamies et al. 2022).

Quality controls (QC) or check points ensure reproducibility of steps in the production process or in the experimental protocols. The QM plan defines which QC need to be performed at which time, in which frequency, and under which conditions, production or application stages. The more robust and well-detailed the production steps are, the less QC steps will be necessary. Also, the more standard experimental protocols (i.e., robust and validated procedures) are implemented, the less need will there be to quality control many steps.

QC measures need to be defined and performed routinely at key production and implementation stages to ensure the quality of the work. Changes in these parameters can be an indicator of underlying problems and help to identify their causes. Once a good QM plan has been established and QC have been defined, it is important to define the acceptable range of data for QC. Such thresholds and ranges are called acceptance criteria (AC). For instance, a QC stem cells may be to measure the viability of cells in an organoid. The AC for the outcome may be that viability must be > 95 %. Another QC measure would be to measure the number of astrocytes in a brain organoid. The AC may be that there must be > 5 % and < 20 % astrocytes, or other pre-defined percentages depending on what specific brain region the test system is aimed at modelling. As a next step, some actions to be taken when an AC criterion is or not fulfilled need to be defined, for instance, discarding an experiment when it is not fulfilled, or proceeding to the next production or experimental step if it is fulfilled.

This process of defining QC, setting AC for them, and then also defining actions upon fulfillment or non-fulfillment, will help to assure that only reliable MPS are produced and used. In GCCP 2.0 guidelines, acceptance criteria define the minimally acceptable state/parameters of a cell culture that will allow to discriminate between good and bad quality (Pamies et al. 2022). This will strongly depend on the respective application / context of use of the model.

Key scientific quality parameters and provisional QC methods will emerge from the initial development of the model and system. Thorough characterization during the early development and optimization phase will drive the identification of suitable quality parameters, selection of test methodologies and the acceptable variations in readouts. This activity should focus on those parameters, which have most influence on organ-specific model behavior, morphology, and overall reproducibility. Understanding the acceptable test measurement range enables the investigator to specify full quality criteria requirement for the model. ATP (adenosine triphosphate) content of a spheroid, for example, is dependent on viability, cell number and oxygen availability. Controlling and specifying the size of the spheroid after aggregation (QC) in a range where no necrosis has been observed during model development, ensures robustness of the ATP assay and a clear correlation with viability.

QCs in routine processes allow for establishment of quality trending over multiple model production runs over time and assessment of reproducibility (batch-to-batch variability). A high reproducibility allows for eventual reduction of the number of checkpoints or of the frequency of measurements. On the contrary, high variation is an indicator that some parameters are not under control and additional checkpoints might need to be included to reduce variability between experiments. Additional QC steps might need to be included if critical parts of the systems are substantially modified or exchanged (e.g,. change of the cell source or donor lot, replacement of a production instrument).

Ideally, a final QC at the end of the process would be sufficient. Intermediate checkpoints are, however, highly valuable from the production-cost perspective and should be identified to enable abortive steps before any expensive final stage. For example, valuable and expensive cells should only be seeded in culturing plates or chips, if they meet high-quality criteria. The earlier potential failures can be sorted out, the less impact they will have on the overall cost of production.

QC need to be established considering several biological, functional, manufacturing, and technological aspects, encompassing different disciplines and expertise input. An MPS consists of a multitude of parts from disciplines that could have different approaches to QM, including cells, media with special additives, hydrogels, chips, and plates, as well as accessory instrumentations and operating system. Each single parts need to adhere to specific QC criteria so that the assembled system has a chance of meeting higher-level criteria as well. Once an organ model is established, functional organ-specific parameters need to be measured and compared to pre-defined acceptance ranges or thresholds, which are strongly dependent on the future applications of the MPS.

QC steps should not interfere with the future performance of the system itself, i.e., their measurement should not influence test system performance (e.g., extensive microscopy can lead to phototoxicity). If reproducibility between MPS batches can be ensured (e.g., chip-to-chip variability) and their production is at larger scales, QC can be limited to a few replicates and destructive end point measurements can be applied.

Although it is not the focus of this manuscript, it is important to mention that there are guidelines and standards that could apply to MPS manufacturing in an industrial setting. Some quality standards such as ISO 9001 “Quality management systems — Requirements”, guide the establishment of the quality management system in any company, that can be inspected by certification bodies that confirm that the company meets the requirements of the standard. In research laboratories, it is feasible to implement a quality system and there are numerous examples operating under ISO 9001. Testing labs can also opt for accreditation under ISO/IEC 17025 “General requirements for the competence of testing and calibration laboratories”, used worldwide as a proof of technical capability of a laboratory for testing and monitoring. Other standards may also apply, for instance companies producing MPS that are intended to be used in clinical settings and can be classified as medical devices have to follow the ISO 13485 (ISO 2016) “Medical devices -- Quality management systems -- Requirements for regulatory purposes”, that describes the quality management system for the design and manufacture of medical devices. Moreover, MPS that are intended to be used as therapeutic applications should follow GMP(EMA 2023; FDA 2023) guidelines during the manufacturing process. Finally, some components or instrumentation that are or can be part of an MPS could fall under specific regulations and thus may require other authorizations for commercialization (e.g. electromagnetic compatibility directive). Of note, the CE Mark is an indication that the manufacturer states the conformity of the product with European health, safety, and environmental protection standards and it should not be taken as an indication of quality or a certification mark.

Whatever level the MPS developer is working at, incorporation of suitable quality controls is crucial to ensure acceptable reproducibility and accuracy of the models, thus, assuring the quality of the results. The following chapters summarize the key quality considerations applicable to the novel MPS cell culture systems, and evaluate the different metrics suitable for different models to assure the reproducibility of scientific data.

Establishing acceptance criteria for the use of MPS

Different perspectives on acceptance criteria

This chapter deals, simply speaking, with the questions: "*when is a test system good enough for use?*" and "*can I trust the data derived from it?*", which are clearly inter-related. It is important to note that here we describe good practices concerning the planning and the performance of experiments, whilst considerations about toxicology or basic research data reporting are not covered in the present study.

First of all, it is important to clearly define what a test system is, and what distinguishes a test method from a test method run(Bal-Price et al. 2018; Krebs et al. 2020; Krebs et al. 2019), considering that each of these elements may be quality-managed.

The test system describes the biological key properties, e.g., a liver organoid, or a muscle-nerve assembloid with all physical (e.g., size), chemical (e.g., matrix components, outer scaffold) and biological (types/percentages of cells contained) details. The above section describes QC and AC for MPS as test systems.

The test method is based on the use of the test system for a specific purpose, i.e., to test a hypothesis. For instance, in toxicology, the hypothesis could be establishing under which conditions a certain compound is toxic; in pharmacology, assessing whether a compound affects the metabolic or signalling status of the test system; in disease biology, understanding whether cells can be infected, or whether they show an unhealthy level of lipid accumulation under certain conditions. All these hypotheses and relative questions can only be answered if the test method contains additional elements (besides the test system itself). Needed details encompass (i) an exposure scheme (e.g., for 2 min or for 24 h; with or without solvent control; at 20 °C or at 37 °C, etc.), (ii) an endpoint definition (i.e., the parameter(s) to be measured, such as hormone secretion, or ATP production or lipid content or levels of certain viral RNAs), and (iii) a prediction model (also called data interpretation procedure), which determines whether the endpoint data (e.g., per organoid) means that the hypothesis can be accepted or rejected, i.e., being able to answer to the question whether or not a compound is toxic or whether or not a molecule is pharmacologically active. For all these elements, QC may be implemented. What is most commonly done is to determine performance parameters of the prediction model, considering e.g., its sensitivity, specificity, balanced accuracy, etc. The performance needs to be determined against some form of ground truth (i.e., absolute knowledge on what would be the correct outcome). In the absence of a direct source of ground truth, some other data set may be defined as "gold standard" and be considered as ground truth. In the past, animal experimental data have often been considered as gold standard when evaluating the performance of cell-based test methods. Altogether this process of

performance evaluation is part of the validation of a test method, as described for toxicological methods e.g., in the OECD guidance document 34 (OECD 2005) . Recently, alternative validation approaches have been discussed. For instance, mechanistic validation (Hartung et al. 2013) may provide an alternative in the absence of sufficient gold standard or ground truth data (Leist et al. 2012; Patlewicz et al. 2013; Patterson et al. 2021; Tigges et al. 2021). Besides the performance (i.e., predictivity), a validation process usually examines the robustness of a test method and its relevance (Hartung et al. 2004; Krebs et al. 2019; Wilcox and Goldberg 2011). Often, this is a multi-step process, beginning with the analysis of in-house reproducibility. In the end, multicenter studies sharing the same materials (including cells and key reagents), devices and protocols can be performed to assess the between-lab reproducibility. This is extremely resource- and time-consuming, and not always possible for commercial MPS solutions. Therefore, discussion is ongoing how information on performance and robustness may be provided by alternative approaches, e.g., for high-throughput testing systems (Judson et al. 2013). An important element of these considerations is to define which information is really required for which type of application. This has resulted in the "fit-for-purpose" concept, described below. An important element of any validation (classical or fit-for-purpose) is the definition of reference materials (i.e., reference chemicals to elicit particular responses in the MPS) (Hoffmann et al. 2008). Often these compounds are classified as positive and negative controls, but many more steps and dimensions are possible. For example, there may be positive controls for a certain strength of effect (weak, medium, strong) or for certain types of effect or measured endpoints (e.g., steatosis, hypertrophy, necrosis etc. in a liver model).

The test run requires a test method that is valid. Moreover, it is important that the individual run, in which the data are produced, is acceptable, and sometimes, this distinction is not always immediately obvious to understand. The validation process makes sure that, in principle, the test method is acceptable. This means that it produces valid data if everything goes well and is done well. A validation is performed only once in the lifetime of a test method, but test runs may be done 1000 times. If they perfectly run 999 times, one cannot be sure that this is the case for run 1000. In order to provide evidence for test runs, the concept of "in-process Acceptance Criteria (AC)" has been introduced. The main application for AC is indeed the control of specific test runs/experiments. If the term AC is used without any further specification, then usually the control of a test run (not of a test method) is meant. In many cases, such AC can be positive and negative controls run along, in the same setup, and for which certain criteria are specified (e.g., negative controls should kill < 5 % of cells, positive controls should kill > 80 % of cells). Importantly, and often neglected, there is some difference between a positive/negative control and an AC. A control compound is a chemical (or condition) for which a certain test method outcome has been defined. An AC is such a control compound with a defined

outcome AND a follow-up rule on what action is taken if the condition is fulfilled/non-fulfilled. For example, if the negative control kills > 5 % of cells then all data from the same plate, or from the same day are discarded. Assays without AC can be run, and they can give data, but it is not easy to have confidence in these data. It is not impossible for two reasons: first, one can retrospectively check whether the positive and negative controls were correct. Thus, one can apply AC retrospectively (this is not perfect, as it allows for some arbitrariness in the definition and the process is not fully transparent). Second, one can make use of historical controls. "Historical controls" are a record of past test runs and the performance of positive and negative controls in them. Historical controls should always be consulted to obtain information on whether a test run is typical or whether it shows unexpected and unplanned changes. This can be done independent of AC. Moreover, if historical controls indicate that in the past 99 % of all runs did not show a problem, then one may assume with high confidence that the present run also was without problems. This is a probabilistic performance assessment, it does not substitute AC, but it indicates at least a certain level of confidence.

To conclude this part: for development and use of MPS, it is important to consider QC for the setup of the MPS, for the test method in which the MPS is used and for the runs of the test method. One can define AC for the MPS as such (these are test system AC, and they need to be done every time a system is set up). One can also provide a QC for the test method: this is generally done in a validation process (done only once). Finally, one can set AC for the validity of test runs: this needs to be applied to each test run and in addition all such data should be collected as "historical controls".

The fit-for purpose (ffp) concept

For the regulatory use of test methods, especially in the area of toxicology, it is mandatory to establish some form of "validity" of the method. This is the basis for recipients and stakeholders having confidence into the method output (e.g. data on the behavior of chemicals). A classical approach to validation establishes reliability, relevance and predictivity of the method. Very expensive and resource-consuming ring trials are often required for this, and the process can take many years. For technical reasons, ring trials are difficult or impossible for complex MPS, as the equipment and expertise may only be available in one or few laboratories. The establishment of predictivity is also a difficult concept when there is a dearth of calibration compounds (positive controls). The concept may be flawed altogether, if predictivity is defined too widely. For this reason, alternative validation concepts have been suggested that focus on "fit-for purpose". More specifically, these concepts suggest that robustness is still to be evaluated; there should also be an evaluation of the relevance of the model; instead of general predictivity, the concept of fitness-for-purpose, i.e. of being able to serve a defined objective (e.g., measuring an AOP key event; providing screen hits for further follow-up;

quantifying a certain endpoint within the model) has been evoked (Judson et al. 2013; Leist et al. 2014; Patlewicz et al. 2013). The concepts of “fit-for-purpose” and test “readiness” for a defined (narrow) purpose are now being considered in several strategies to make validation of methods more flexible (Judson et al. 2013; Patlewicz et al. 2013).

For the development of MPS, this has several consequences and implications: Most important is that individual aspects of validation may be considered one after another. Moreover, they may be given different weight and priority. Often the MPS as such is developed in order to have some model features (e.g., to mimic a certain tissue). This process generates a test system (e.g., a device or a cell culture) and not a test method. This test system may then be used in different contexts. Only one of them is to use the test system as element of a test method in toxicology or pharmacology. If the latter is intended, other elements need to be added (e.g., a prediction model and an exposure scheme). A complete test method is also defined by its purpose (testing of a certain hypothesis). Only when this is defined, a fit-for purpose evaluation of the overall method can be initiated.

The difference between a test system (a certain MPS setup) and a test method (using this MPS) may become more evident from following example(s). A liver spheroid, within a recirculating device is a test system. The MPS as such has no particular purpose (i.e., it may be used for many different purposes), no exposure scheme and no data interpretation procedure. A test method may be based on the above MPS. But the method would also define a 7-day exposure period to chemicals, use certain measurements to generate output data, and have a certain performance with respect to a set of known toxicants and non-toxicants. The purpose would be to predict e.g. necrotic liver injury. If the model is validated, it is validated for exactly this purpose. Possibly, the same MPS may be used to study steatosis. This would change the purpose of the test method, and for this new purpose, another set of control compounds would be required. The prediction model, the endpoints assessed, and the exposure scheme would most likely change as well. Thus, a new validation would be required. The example shows that the same MPS (test system), may be an element of two entirely different test methods, with two different purposes.

The concept of purpose can limit the applicability domain of a test method, and it adds heavy workload on the implementation of an MPS into a test method. But it also has an enormous conceptual and practical strength. This principle makes the validation of complex methods at all possible. Only because of this concept can methods based on MPS be validated. The point is that the function of a system, according to a specific purpose, is validated, instead of the descriptive state of system components. This represents an advantage, considering that describing complex biological (test) systems in a

comprehensive manner may not be possible. There is no descriptive feature that would even allow to claim absolute identity of two cell cultures (Gutbier et al. 2018; Kleensang et al. 2016). If one uses Short tandem repeat (STR) typing (Almeida and Korch 2004), this only shows that they are not massively different and that they originate from the same cell. If one completely sequences their genome, it is impossible to exclude small subpopulations and to exclude epigenetic differences; and if the latter was possible the proteome might still show hundreds of differences concerning post-translational modifications and protein localizations, if the cells underwent a different culture history. If larger and larger efforts cannot define even relatively simple systems, how can one then describe MPS and establish QC? The solution lies in not describing the state as such, but the performance, according to a certain purpose. A system is fit for a certain purpose, if one can establish that questions concerning this purpose can be answered sufficiently well. If the purpose is to identify hepatotoxic compounds with 80 % accuracy, one may test a panel of known compounds, and check whether this would work. If this test is designed sufficiently broad and robust, and if it turns out positive, then the system is fit to answer questions about hepatotoxicants. This is in simple terms what is called a ffp validation. Stepwise, one defines the purpose, defines the robustness (reproducibility) required, establishes criteria for relevance and checks their appropriateness in a given context, and finally checks performance with defined control conditions within the defined purpose. Finally, after the ffp validation of the test method, one would define test run AC, which are again suited for the particular purpose. An important implication of this concept is that the system may have many shortcomings, or black spots (unknown features). But all conditions required for a given purpose are fulfilled. The second, complementary implication is that the system is only “validated” for this purpose, not for any other use.

An important consequence of ffp validation is a different level of requirement for ring trials. If a system’s purpose is to be used within a given company or lab environment, and not “in general”, it is not essential for a ffp validation to perform a ring trial. This concept shows that ffp validations relieve the validation process of some heavy, resource-consuming loads. Vice versa, there is a danger that criteria are defined with a very heterogeneous stringency and are therefore hard to accept in general. To avoid such concerns, there is the need to more clearly define elements of ffp validation (van der Zalm et al. 2022). One idea is not to define a system as validated or not validated, but rather to see this as a gradual process, defining “readiness levels” (Bal-Price et al. 2019). Assays may be used for a small number of applications when they have a lower readiness level; and they may become more broadly applicable when they have a higher readiness level.

Transition from formal assay validation to in-process validation

By relying less on formal validation a test as such, the demands for in-process AC have increased. This means that with a more flexible system, concerning resource investment into the test system, more efforts should be made to define AC of each run. There are other areas where such approaches have become quite common, as in the testing of epidemiological hypothesis, social and political science studies, or in drug testing. In many fields, the test method used to study a hypothesis cannot be extensively validated prior to the study. In such cases, there is a high demand to run many layers of consistency controls within the study. For example, in drug testing, not only positive (competitor drug) and negative (placebo) controls are run, but also historical records on control *vs* disease populations are considered. Inclusion and exclusion criteria are strictly defined and closely monitored, and many additional data on other endpoints are acquired to control whether the trial is consistent within itself and with all general medical knowledge. Such approaches use complex crossover designs and an extensive baseline and post-study monitoring to increase confidence into the data. If one would apply this approach to MPS-based test systems, they may be validated only to a minimal extent (e.g., for producing robust baseline data). Instead, each test run would be accompanied by a well-chosen and comprehensive set of AC. If all control compounds and control conditions are within the limits set for acceptance of AC, this may be considered as an "in-run validation". Although this example points out a relatively radical approach, it is meant to illustrate the general principle that extensive validation can relief the need for AC (i.e., considering a reduced number of AC acceptable, but never fully substitute them). Instead, a less stringent validation increases the requirement for very extensive in-run AC to allow sufficient confidence into the data.

Cell source quality controls

The source of cells used to generate MPS often comes from stem cells, such as human induced pluripotent stem cells (iPSCs), as well as primary cultures isolated from biopsies, or cell lines. The selection of a cell source can be based on the availability, variability and quantity of the cells required to seed them into the MPS device. The acquisition of primary cells from human biopsies is a bottleneck in the research process because it requires significant resources for donor recruitment and biobanking. The ability to generate substantial quantities of iPSCs offers a highly attractive cell source for MPS research, and it serves as the main focus of this paper. However, we acknowledge that primary cells can also be used to develop MPS and provide certain advantages; quality specifications can be found elsewhere (Geraghty et al. 2014; Pamies et al. 2022).

The selection of a cell source and the establishment of QC during these initial steps is essential to assure the development of good cell culture practice. Misidentification, contamination, or other early technical errors may lead to wasted resources (money and time). Obtaining a detailed description of the cells (e.g., how they were created, species, origin, special features, karyotype, gender, health state, and age) is important and needs to be well documented and controlled. Documentation of QC on contaminants such as mycoplasma, human viruses, fungi and other pathogens is very important (GIVIMP 2018; Pamies et al. 2022; Pistollato et al. 2022a). Some studies have shown that misidentification and mycoplasma contamination are still common issues (Horbach and Halffman 2017; Huang et al. 2017; Olarerin-George and Hogenesch 2015; Timenetsky et al. 2006). Therefore, cells sources from cell banks should already be quality controlled to avoid contamination and cell misidentification. It is also recommended to establish internal QC controls including mycoplasma testing, identity testing, viral screening, especially when receiving a new line in the laboratory (Pamies et al. 2022).

Human iPSCs are becoming a more popular cell source for scientists working on *in vitro* models. They provide access to diverse human tissues without many of the logistical and ethical challenges involved in sourcing for multiple primary tissues and permitting increased standardization by providing cells of consistent genetic background for experimental work (Kuse and Taniguchi 2019). However, we must keep in mind that several ethical concerns relating to patient-sensitive information and consent are still very important to take into consideration.

The European Bank for induced Pluripotent Stem Cells (EBiSC), have recently published an outline for the establishment and implementation of a quality control regime suitable for a large-scale operational setting (O'Shea et al. 2020). These recommended QC can be scaled for academic setting to a certain extent. In addition, detailed quality criteria for *in vitro* human iPSC-derived models have been recently published (Pistollato et al. 2022b) and an ISO standard (ISO 24603) for both human and mouse PSCs for research use has also been published(ISO 2022). This document contains a list of generic minimum QC tests and examples of helpful methodologies and acceptance criteria for human stem cell selection. Furthermore, some of the most common and relevant morphological, biochemical, and functional endpoints that can be used as acceptance criteria for iPSC-derived models have been discussed (Pistollato et al. 2022b).

References

Almeida JL, Korch CT (2004) Authentication of Human and Mouse Cell Lines by Short Tandem Repeat (STR) DNA Genotype Analysis. In: Markossian S, Grossman A, Brimacombe K, et al. (eds) Assay Guidance Manual. Bethesda (MD)

Bal-Price A, Hogberg HT, Crofton KM, et al. (2019) Recommendation on Test Readiness Criteria for New Approach Methods in Toxicology: Exemplified for Developmental Neurotoxicity (vol 35, pg 306, 2018). Altex-Altern Anim Ex 36(3):506-506 doi:10.14573/altex.1904112

Bal-Price A, Hogberg HT, Crofton KM, et al. (2018) Recommendation on test readiness criteria for new approach methods in toxicology: Exemplified for developmental neurotoxicity. ALTEX 35(3):306-352 doi:10.14573/altex.1712081

EMA (2023) Good manufacturing practice. In. https://www.ema.europa.eu/en/human-regulatory/research-development/compliance/good-manufacturing-practice#registration-of-manufacturers-of-active-substances-section

FDA (2023) Current Good Manufacturing Practice (CGMP) Regulations. In. https://www.fda.gov/drugs/pharmaceutical-quality-resources/current-good-manufacturing-practice-cgmp-regulations

Geraghty RJ, Capes-Davis A, Davis JM, et al. (2014) Guidelines for the use of cell lines in biomedical research. Brit J Cancer 111(6):1021-1046 doi:10.1038/bjc.2014.166

GIVIMP O- (2018) Guidance Document on Good In Vitro Method Practices (GIVIMP) for the Development and Implementation of In Vitro Methods for Regulatory Use in Human Safety Assessment. . OECD Publishing

Gutbier S, May P, Berthelot S, et al. (2018) Major changes of cell function and toxicant sensitivity in cultured cells undergoing mild, quasi-natural genetic drift. Arch Toxicol 92(12):3487-3503 doi:10.1007/s00204-018-2326-5

Hartung T, Bremer S, Casati S, et al. (2004) A modular approach to the ECVAM principles on test validity. Altern Lab Anim 32(5):467-72 doi:10.1177/026119290403200503

Hartung T, Hoffmann S, Stephens M (2013) Mechanistic validation. ALTEX 30(2):119-30 doi:10.14573/altex.2013.2.119

Hoffmann S, Edler L, Gardner I, et al. (2008) Points of reference in the validation process: the report and recommendations of ECVAM Workshop 66. Altern Lab Anim 36(3):343-52 doi:10.1177/026119290803600311

Horbach SPJM, Halffman W (2017) The ghosts of HeLa: How cell line misidentification contaminates the scientific literature. Plos One 12(10) doi:ARTN e0186281

10.1371/journal.pone.0186281

Huang YQ, Liu YH, Zheng CY, Shen C (2017) Investigation of Cross-Contamination and Misidentification of 278 Widely Used Tumor Cell Lines. Plos One 12(1) doi:ARTN e0170384

10.1371/journal.pone.0170384

ISO (2016) ISO 13485:2016 Medical devices Quality management systems Requirements for regulatory purposes. In. https://www.iso.org/standard/59752.html 2024

ISO (2022) ISO 24603:2022(en) Biotechnology — Biobanking — Requirements for human and mouse pluripotent stem cells. In. https://www.iso.org/obp/ui/#iso:std:iso:24603:ed-1:v1:en Accessed 21/02/2024 2024

Judson R, Kavlock R, Martin M, et al. (2013) Perspectives on validation of high-throughput assays supporting 21st century toxicity testing. ALTEX 30(1):51-6 doi:10.14573/altex.2013.1.051

Kleensang A, Vantangoli MM, Odwin-DaCosta S, et al. (2016) Genetic variability in a frozen batch of MCF-7 cells invisible in routine authentication affecting cell function (vol 6, 28994, 2016). Sci Rep-Uk 6 doi:ARTN 33011

10.1038/srep33011

Krebs A, van Vugt-Lussenburg BMA, Waldmann T, et al. (2020) The EU-ToxRisk method documentation, data processing and chemical testing pipeline for the regulatory use of new approach methods. Arch Toxicol 94(7):2435-2461 doi:10.1007/s00204-020-02802-6

Krebs A, Waldmann T, Wilks MF, et al. (2019) Template for the description of cell-based toxicological test methods to allow evaluation and regulatory use of the data. ALTEX 36(4):682-699 doi:10.14573/altex.1909271

Kuse Y, Taniguchi H (2019) Present and Future Perspectives of Using Human-Induced Pluripotent Stem Cells and Organoid Against Liver Failure. Cell Transplant 28(1_suppl):160S-165S doi:10.1177/0963689719888459

Leist M, Hasiwa N, Daneshian M, Hartung T (2012) Validation and quality control of replacement alternatives - current status and future challenges. Toxicol Res-Uk 1(1):8-22 doi:10.1039/c2tx20011b

Leist M, Hasiwa N, Rovida C, et al. (2014) Consensus report on the future of animal-free systemic toxicity testing. ALTEX 31(3):341-56 doi:10.14573/altex.1406091

O'Shea O, Steeg R, Chapman C, Mackintosh P, Stacey GN (2020) Development and implementation of large-scale quality control for the European bank for induced Pluripotent Stem Cells. Stem Cell Res 45:101773 doi:10.1016/j.scr.2020.101773

OECD (2005) GUIDANCE DOCUMENT ON THE VALIDATION AND INTERNATIONAL ACCEPTANCE OF NEW OR UPDATED TEST METHODS FOR HAZARD ASSESSMENT. In: NTP. https://ntp.niehs.nih.gov/sites/default/files/iccvam/suppdocs/feddocs/oecd/oecd-gd34.pdf Accessed 28/02/2024 2024

Olarerin-George AO, Hogenesch JB (2015) Assessing the prevalence of mycoplasma contamination in cell culture via a survey of NCBI's RNA-seq archive. Nucleic Acids Res 43(5):2535-2542 doi:10.1093/nar/gkv136

Pamies D, Leist M, Coecke S, et al. (2022) Guidance document on Good Cell and Tissue Culture Practice 2.0 (GCCP 2.0). ALTEX 39:30-70 doi:10.14573/altex.2111011

Patlewicz G, Simon T, Goyak K, et al. (2013) Use and validation of HT/HC assays to support 21st century toxicity evaluations. Regul Toxicol Pharm 65(2):259-268 doi:10.1016/j.yrtph.2012.12.008

Patterson EA, Whelan MP, Worth AP (2021) The role of validation in establishing the scientific credibility of predictive toxicology approaches intended for regulatory application. Comput Toxicol 17:100144 doi:10.1016/j.comtox.2020.100144

Pistollato F, Bal-Price A, Coecke S, et al. (2022a) Quality criteria for in vitro human pluripotent stem cell-derived models of tissue-based cells. Reprod Toxicol 112:36-50 doi:10.1016/j.reprotox.2022.06.003

Pistollato F, Bal-Price A, Coecke S, et al. (2022b) Quality criteria for in vitro human pluripotent stem cell-derived models of tissue-based cells. Reprod Toxicol 112:36-50 doi:10.1016/j.reprotox.2022.06.003

Tigges J, Bielec K, Brockerhoff G, et al. (2021) Academic Application of Good Cell Culture Practice for Induced Pluripotent Stem Cells. Altex-Altern Anim Ex 38(4):595-614 doi:10.14573/altex.2101221

Timenetsky J, Santos LM, Buzinhani M, Mettifogo E (2006) Detection of multiple mycoplasma infection in cell cultures by PCR. Braz J Med Biol Res 39(7):907-914 doi:Doi 10.1590/S0100-879x2006000700009

van der Zalm AJ, Barroso J, Browne P, et al. (2022) A framework for establishing scientific confidence in new approach methodologies. Arch Toxicol 96(11):2865-2879 doi:10.1007/s00204-022-03365-4

Wilcox N, Goldberg A (2011) Food for Thought ... on Validation. A Puzzle or a Mystery: an Approach Founded on New Science. Altex-Altern Anim Ex 28(1):3-8 doi:DOI 10.14573/altex.2011.1.003

Supplementary material 2

Liver MPS

Aspects making liver MPS unique

Since the liver is the main organ involved in metabolism of chemicals and drugs, modeling its function is important in drug development, particularly for drug-drug interaction (DDI) and toxicity studies. Besides, as the chronic exposure of the liver to chemicals and drugs can lead to hepatotoxicity, several liver disease models have been generated in the past (Kulsharova and Kurmangaliyeva 2021; Moradi et al. 2020). In recent years, 3D (liver spheroids, organoids) and OoC liver cell models have been presented as an alternative to less physiological 2D cell culture models and their application have progressed significantly (Duval et al. 2017).

The liver is composed of numerous cell types. Hepatocytes which are parenchymal cells, constitute up to 80 % of the liver mass and 60 % of its cellular composition and are primarily involved in various processes, such as the synthesis of plasma proteins, enzyme production, and metabolism. Cholangiocytes (biliary epithelial cells), sinusoidal endothelial cells (LSECs), hepatic stellate cells (HSCs), and Kupffer cells (KCs) belong to the non-parenchymal cells. They have supporting functions in immune surveillance (e.g., KCs) (Dixon et al. 2013) and play an important role in the progression of certain liver diseases, such as the non-alcoholic steatohepatitis (NASH) after being activated (e.g., HSCs) (Thompson and Takebe 2020; Zisser et al. 2021).

The described hepatic cell types can be represented in liver spheroids using well-established cell lines, such as the immortalized human hepatoma cell lines HepaRG, HepG2, and Huh-7, among others (Xu 2021). Although the first liver spheroids were reported in 1985 (Landry et al. 1985), their use has become more and more popular only during the last decade. Spheroids offer a more physiological approach compared to the conventional 2D hepatocyte cultures. Given their extended maintenance of hepatic functions, they are particularly useful for repeated-dose studies, chronic toxicity studies or to assess long-term drug effects. Their application reaches from hepatotoxicity studies, long-term clearance predictions and CYP induction studies, over their usage for disease models such as NASH, liver fibrosis and infectious diseases (Arez et al. 2021; Ingelman-Sundberg and Lauschke 2022; Kanebratt et al. 2021). Various spheroid culture methods have been described, enabling the formation of spheroids within a few days (Bialkowska et al. 2020; Mannaerts et al. 2020; Ryu et al. 2019). For instance, they can be prepared with the use of ultra-low attachment (ULA) plates, whereby the cell suspension is directly seeded into pretreated U-shaped wells preventing cell attachment, or by using the hanging-drop technique. In the latter, the cell suspension aggregates into spheroids in a drop of

medium placed on the inside of the cell culture lid (de Souza et al. 2021), which is then reversed to result in hanging drops. Recently, bioprinting has emerged as a useful application for fast spheroid formation within 24 hours (Hong and Song 2021).

Status of liver MPS development and examples of purposes of use

As an example, Messner et al. developed a liver fibrosis model using co-cultured scaffold-free liver spheroids, comprised of HepaRG, immortalized HSCs (hTERT-HSC), and a human leukemia monocytic cell line (THP-1), to study miRNAs specifically released after the exposure to the liver fibrosis stimulating drug methotrexate, for identifying fibrosis-specific miRNA as extracellular biomarkers (Messner et al. 2021). As it is particularly difficult to determine the metabolic stability for low-clearance compounds in short-term incubation models (like liver microsomes), advanced 3D models are upcoming tools to overcome this challenge. They do not only contain a variety of hepatic drug-metabolizing enzymes and transporters, such as CYP450 and the p-glycoprotein, but also maintain their enzymatic activity over several weeks allowing incubations over longer time periods compared to the common 2D cell cultures (Bonn et al. 2016; Kanebratt et al. 2021; Riede et al. 2021). The liver spheroids were either cultured alone or pooled, and were proved and compared for their cell morphology, hepatic enzyme and transporter expression, and hepatocellular functions.

Moreover, liver organoids (also called hepatic organoids) originated from different stem cell sources have been developed over the last decade (Harrison et al. 2021; Liu et al. 2023; Thompson and Takebe 2020). They represent an alternative approach to common cell lines like hepatocytes, LSECs, and KCs, which demonstrate low proliferation *in vitro* and difficulties in maintaining their phenotype [67]. Liver organoids can be generated from iPSCs and embryonic stem cells (ESCs), and co-cultured in combination with adult cells such as mesenchymal or epithelial cells, helping to obtain the desired phenotype and functional maturity (Baxter et al. 2015). iPSCs offer the potential to differentiate into the specific types of liver cells, such as hepatocytes, cholangiocytes, LSECs, and KCs. Differentiation is achieved by adding a combination of cytokines and growth factors which mimic the developmental processes to the specific cultures. Several methods and transcription factor combinations have been evaluated for generating *bona fide* and clinical grade organoids with the result, that 3D cultures better mimic the *in vivo* environment than 2D cell cultures of iPSCs or primary cells [67]. In general, hepatic organoids, similar to organoids of other organs, present the advantage of retaining important physiological characteristics of the liver. This culture method allows recreating an *in vitro* environment that closely imitates the *in vivo* setting. 3D culture has shown to improve longevity and function of the models (Bell et al. 2016) and other organ specific features, such as retinol storage in lipid droplets, activation in response to cytokines, and wound healing capability (Coll et al. 2018). Thus, their

application can target the study of related disease mechanisms, the development of new drugs and toxicity testing, and they also offer promising models for clinical applications (Ding et al. 2016; Olgasi et al. 2020).

Several examples of organoids in the literature have shown their ability to recreate the intricate multicellular complexity and 3D architecture that underlie normal organ function, including cell-to-cell and cell-to-ECM interactions (Guan et al. 2021; Ouchi et al. 2019; Takebe et al. 2014; Tanimizu et al. 2021; Wu et al. 2019). Tanimizu et al. reported the generation of a hepatobiliary tubular organoid (HBTO) using mouse hepatocyte progenitors and cholangiocytes (Tanimizu et al. 2021). In this model, the hepatocytes can form the bile canalicular network and secrete metabolites into the canaliculi, which are further transported into the biliary tubular structure. In addition, they showed long-term maintenance of metabolic functions such as albumin secretion and CYP450 activity, which are present in this model.

OoC liver cell models can provide dynamic "blood" flow conditions by medium perfusion, a physiological oxygen gradient, and mechanical stimulations within their micro-channels (e.g., shear stress). By applying chemical concentration gradients, biological processes such as chemotaxis, metastasis, cell migration and differentiation, wound healing, and immune responses can be regulated (Beckwitt et al. 2018; Moradi et al. 2020). Moreover, liver metabolism is a key mediator of drug induced toxicity in other organs. Thus, co-culturing of cells in a multi-organ-on-a-chip platform allows the modeling of organ-to-organ interactions (Ribeiro et al. 2019).

To date, various OoC liver systems have been described in the literature. For example, Jang et al. used a liver-chip cultured with rat, dog, or human hepatocytes, LSECs, KCs, and HSCs to study the mechanism of inflammatory responses in immune-mediated drug-induced liver injury (DILI) (Jang et al. 2019). Deng et al. developed an alternative approach by incorporating several immortalized cell lines (LX-2, HepG2, EAhy926, U937) to a 3D liver sinusoid-on-a-chip device and by simulating the physiological environment like blood flow and the biliary efflux in the opposite direction. This model showed comparable results to a gold standard primary hepatocyte model, hence proposed the model as an alternative for hepatotoxicity evaluation and DDIs (Deng et al. 2019). Lohasz et al. made use of a gravity-driven microfluidic system where they cultivated primary human liver microtissues (hLiMTs) combined with a carcinoma cell line (HCT116) and tumor microtissues (TuMTs), to study DDIs of two anticancer drugs and an antiviral drug (Lohasz et al. 2020). For mimicking a liver tumor microenvironment, Lu et al. introduced a biomimetic 3D liver tumor-on-a-chip, based on HepG2 cells and the integration of essential components derived from decellularized liver matrix integrated with gelatin methacryloyl

(gelMA) (Lu et al. 2018). This system can be used for pharmacological and toxicity studies of anticancer model drugs. As already described, effects of organ-to-organ interactions can be assessed by using multi-organ systems. Bovard et al. observed in their study, that the liver metabolism can also have a protective effect on other organs (Bovard et al. 2018). Through the co-culture of HepaRG spheroids and normal human bronchial epithelial (NHBE) cells at the air-liquid interface, they developed a lung-liver-on-a-chip that was able to demonstrate detoxification of aflatoxin B1 by liver metabolism.

The OoC technology has also been used for toxicology approaches. For example, there is a commercially available platform for high-throughput hepatotoxicity screening that has been used to test a large set of (159) compounds. Within this platform, iPSC-derived hepatocytes were co-cultured with endothelial cells and THP-1 cells (Bircsak et al. 2021). Yin et al. were able to show liver metabolism-dependent cardiotoxicity of an antidepressant drugs and thus predicting cardiac safety by using a iPSC-derived multi-organoid-on-a-chip system (Yin et al. 2021). After the formation of self-organized organoids from iPSCs, the 3D liver and heart MPS were co-cultured within compartmentalized chambers. Skardal et al. in a 3D liver / heart model showed that the liver organoids were able to metabolize a sufficient amount of propranolol to allow epinephrine to induce a beat rate increase (Skardal et al. 2017).

Aspects of quality control of liver MPS

Successful cell growth and cell functionality are the two major points considered while developing a MPS (Kulsharova et al. 2021). To characterize liver cell models, different markers can be used, some of which can be continuously monitored in the medium and others that require imaging or cell lysis. A general key aspect to assess ideally before, during, and after the development of MPS is the cell viability that can be tested by several test methods like the ATP assay, trypan blue, LDH assay, and more (Vinken and Hengstler 2018). At tissue level, hepatocytes can be identified by their polarized polygonal shape and the formation of bile canaliculi structures already after one day of cell culturing using brightfield imaging. Key functional characteristics are the expression of MRP2 as a marker for hepatic bile acid transport and the secretion of albumin (Vinken and Hengstler 2018) (regulating blood osmolarity) (Palakkan et al. 2015) and urea (ammonia detoxification) (Vilstrup 1980) as well described functional hepatic markers, whereby their secretion strongly depends on the culture time (Moradi et al. 2020; Vinken and Hengstler 2018). Moreover, the storage of glycogen to examine functionality can be tested performing the periodic acid-Schiff stain of the cells. The ability to perform hepatic xenobiotic metabolism can be evaluated by assessing the expression of phase I & II metabolism enzymes (e.g., CYP3A4). Still, expression proven at protein level and gene level should be completed by measuring the proper activity of the enzymes by the use of known reference enzyme substrates (e.g., phenacetin (CYP1A), or midazolame (CYP3A) (Kulsharova and Kurmangaliyeva 2021), (Pelletier et al. 2013; Vinken

and Hengstler 2018). Generally, the determination of hepatocytes at cell level can be done by determining expression levels of several transcription factors, such as HNF4-α, HNF6, CEBP-α, PROX1, or GATA4, crucial for liver organogenesis (Maepa and Ndlovu 2020). Since HSCs are involved in the storage of Vitamin A, its presence after incubation with retinol can be visualized using microscopy and fluorescent imaging (Koui et al. 2017). The enzyme Lecithin retinol acyltransferase (LRAT), involved in retinol metabolism, plays an important role in the formation of the HSC lipid droplets and its expression can be determined as a functional key aspect (Koui et al. 2021). In general, LSECs can be characterized by their expression of endothelial cells markers such as FCGR2B, LYVE1, STAB1 or STAB2 (De Smedt et al. 2021). Because KCs play a role in immunosurveillance, these cells should express macrophage markers like CD11, CD14, CD68, CD163, or CD32. Functional KCs should show a response to the stimulation with pro-inflammatory mediators (e.g., LPS) with the secretion of IL-6 or TNF-α that can be detected in a biochemical assay (Tasnim et al. 2019). Cholangiocytes can be morphologically identified as biliary epithelium and the formation of primary cilia assessed by the visualization of α-tubulin with fluorescent imaging. Further, biliary expression markers for cholangiocytes such as CK19, CK18, CK7, CFTR, PKD2, AE2, AQP1, ASBT or SSTR-2 can be determined. The positive activation of calcium signaling following the response to ATP, as a feature of a purinergic receptor activity on primary cilia, illustrates a functional characteristic for the cells and can be determined in a fluorometric calcium assay (De Assuncao et al. 2015; Maepa and Ndlovu 2020).

In terms of application, non-parenchymal cells like the KCs and HSCs are commonly used to generate MPS for disease modeling (e.g., liver fibrosis). For instance, KCs act in the pathogenesis of different liver diseases, and inflammatory responses were shown to be non-recapitulating when only culturing monocultures of hepatocytes (Tasnim et al. 2019). Hepatocytes are regularly utilized to evaluate acute and long-term toxic effects on the liver, for drug screening, ADME studies, and disease models (Guguen-Guillouzo and Guillouzo 2010; Williams 2018). In this case, the long-term stability and the evaluation of functional characteristics as described previously (enzyme activity, albumin secretion etc.) are important points to consider. To correctly predict toxic effects on the cells, the use of known liver-toxic reference compounds (e.g., acetaminophen, valproic acid) can help to characterize the system (Vinken and Hengstler 2018).

The following table S1 summarizes parameters to characterize different iPSC-derived mature liver cell types in a MPS. Characterization for HSCs is considered for the maturation to the quiescent cell type and not to the activated one.

Table S1. Recommendation of quality control for liver Microphysiological Systems (MPS).				
**The general features of the MPS**	**Key morphological, biochemical, and functional parameters (or endpoints) to characterize the model**	**Exemplary analytical methods**	**Acceptance criteria**	**References**
**All liver cell types**	Viability	Ideally an early (ATP assay, trypan blue) and late (lactate dehydrogenase assay) key event of cytotoxicity	> 90 % viability	(Vinken and Hengstler 2018)
**Hepatocyte cell (HC) identity and function**	Morphology	Microscopy	Polarized polygonal cells	(Vinken and Hengstler 2018)
	Morphology	Microscopy,<br>Immunofluorescence, qPCR or western blot (MRP2)	Formation of bile canaliculi after one day (> 20 %),<br>Detection of MRP2 at protein and gene level	(Vinken and Hengstler 2018)
	Albumin	Albumin ELISA assay,<br>Immunofluorescence	> (37 µg/d) per 10^6 cells[a],<br>Detection of albumin at protein level	(Baudy et al. 2020; Moradi et al. 2020)
	Secretion of urea	Urea ELISA assay	> (56 µg/d) per 10^6 cells[b]	(Moradi et al. 2020) (Baudy et al. 2020; Bircsak et al. 2021; Lee-Montiel et al. 2021)
	Storage of glycogen	Periodic acid-Schiff stain (glycogen)	Presence of glycogen in staining	(Agarwal et al. 2008)

	Expression of phase I & II enzymes[c] (CYP3A4)	Immunofluorescence, qPCR or western blot	Detection of CYP3A4 at protein and gene level	(Kulsharova and Kurmangaliyeva 2021)
	Activity of phase I & II enzymes	CYP activity assay	Formation of metabolites	(Pelletier et al. 2013)
	Expression of transcription factors: HNF4-α, HNF6, CEBP-α, PROX1, GATA4	Immunofluorescence, qPCR or western blot	Detection of marker at protein and gene level	(Maepa and Ndlovu 2020)
	Positive response to toxic reference compounds	Exposure to acetaminophen (1-2 mM), valproic acid	Decrease of cell viability	(Vinken and Hengstler 2018)
**Stellate cell (HSC) identity and function**	Expression of (LRAT)	Immunofluorescence, qPCR or western blot	Detection of LRAT at protein and gene level	(Koui et al. 2021)
	Storage of Vitamin A	Microscopy,<br>Fluorescent imaging after incubation with retinol (vitamin A)	Visualization of Vitamin A lipid droplets,<br>Detection of vitamin A at protein level	(Koui et al. 2017)
**Endothelial cell (LSEC) identity and function**	Expression of endothelial cell markers: FCGR2B, LYVE1, STAB1, STAB2	Immunofluorescence, qPCR or western blot	Detection of marker at protein and gene level	(De Smedt et al. 2021)
**Kupffer cell (KC) identity and function**	Expression of macrophage markers: CD11, CD14, CD68, CD163, CD32	Immunofluorescence, qPCR or western blot	Detection of marker at protein and gene level	(Tasnim et al. 2019)
	Stimulation-dependent secretion of IL6, TNF-α	Exposure to LPS followed by ELISA assay	IL6: > 800 pg/mL (after 6 h, 100 ng/mL LPS)[d]<br>TNF-α: > 4000 pg/mL (after 24 h, 100 ng/mL LPS)[d]	(Knolle et al. 1995; Luckey et al. 2002; Tasnim et al. 2019)

**Cholangiocyte cell identity and function**	Morphology	Microscopy	Biliary epithelium	(De Assuncao et al. 2015)
	Expression of cholangiocyte markers: CK19, CK18, CK7, CFTR, PKD2, AE2, AQP1, ASBT, SSTR-2	Immunofluorescence, qPCR or western blot	Detection of marker at protein and gene level	(De Assuncao et al. 2015; Maepa and Ndlovu 2020)
	Expression of primary cilia marker: α-tubulin	Immunofluorescence	Detection of α-tubulin at protein level	(De Assuncao et al. 2015)
	Activation of calcium signaling in response to ATP	Fluorometric calcium assay	Positive RFU signal	(De Assuncao et al. 2015)

[a]acceptance criteria proposed on literature data (Moradi et al. 2020).
[b]acceptance criteria proposed on literature data (Lee-Montiel et al. 2021; Moradi et al. 2020).
[c]example for CYP3A4.
[d]acceptance criteria proposed on literature data (Knolle et al. 1995; Luckey et al. 2002; Tasnim et al. 2019).

References

Agarwal S, Holton KL, Lanza R (2008) Efficient differentiation of functional hepatocytes from human embryonic stem cells. Stem Cells 26(5):1117-1127 doi:10.1634/stemcells.2007-1102

Arez F, Rodrigues AF, Brito C, Alves PM (2021) Bioengineered Liver Cell Models of Hepatotropic Infections. Viruses 13(5) doi:10.3390/v13050773

Baudy AR, Otieno MA, Hewitt P, et al. (2020) Liver microphysiological systems development guidelines for safety risk assessment in the pharmaceutical industry. Lab Chip 20(2):215-225 doi:10.1039/c9lc00768g

Baxter M, Withey S, Harrison S, et al. (2015) Phenotypic and functional analyses show stem cell-derived hepatocyte-like cells better mimic fetal rather than adult hepatocytes. J Hepatol 62(3):581-589 doi:10.1016/j.jhep.2014.10.016

Beckwitt CH, Clark AM, Wheeler S, et al. (2018) Liver 'organ on a chip'. Exp Cell Res 363(1):15-25 doi:10.1016/j.yexcr.2017.12.023

Bell CC, Hendriks DFG, Moro SML, et al. (2016) Characterization of primary human hepatocyte spheroids as a model system for drug-induced liver injury, liver function and disease. Sci Rep-Uk 6 doi:ARTN 25187

10.1038/srep25187

Bialkowska K, Komorowski P, Bryszewska M, Milowska K (2020) Spheroids as a Type of Three-Dimensional Cell Cultures-Examples of Methods of Preparation and the Most Important Application. Int J Mol Sci 21(17) doi:10.3390/ijms21176225

Bircsak KM, DeBiasio R, Miedel M, et al. (2021) A 3D microfluidic liver model for high throughput compound toxicity screening in the OrganoPlate (R). Toxicology 450 doi:ARTN 152667

10.1016/j.tox.2020.152667

Bonn B, Svanberg P, Janefeldt A, Hultman I, Grime K (2016) Determination of Human Hepatocyte Intrinsic Clearance for Slowly Metabolized Compounds: Comparison of a Primary Hepatocyte/Stromal Cell Co-culture with Plated Primary Hepatocytes and HepaRG. Drug Metab Dispos 44(4):527-533 doi:10.1124/dmd.115.067769

Bovard D, Sandoz A, Luettich K, et al. (2018) A lung/liver-on-a-chip platform for acute and chronic toxicity studies. Lab Chip 18(24):3814-3829 doi:10.1039/c8lc01029c

Coll M, Perea L, Boon R, et al. (2018) Generation of Hepatic Stellate Cells from Human Pluripotent Stem Cells Enables In Vitro Modeling of Liver Fibrosis. Cell Stem Cell 23(1):101-+ doi:10.1016/j.stem.2018.05.027

De Assuncao TM, Sun Y, Jalan-Sakrikar N, et al. (2015) Development and characterization of human-induced pluripotent stem cell-derived cholangiocytes (vol 95, pg 684, 2015). Lab Invest 95(10):1218-1218 doi:10.1038/labinvest.2015.99

De Smedt J, van Os EA, Talon I, et al. (2021) PU.1 drives specification of pluripotent stem cell-derived endothelial cells to LSEC-like cells. Cell Death Dis 12(1) doi:ARTN 84

10.1038/s41419-020-03356-2

de Souza IR, Canavez A, Schuck DC, et al. (2021) Development of 3D cultures of zebrafish liver and embryo cell lines: a comparison of different spheroid formation methods. Ecotoxicology 30(9):1893-1909 doi:10.1007/s10646-021-02459-6

Deng J, Zhang XL, Chen ZZ, et al. (2019) A cell lines derived microfluidic liver model for investigation of hepatotoxicity induced by drug-drug interaction. Biomicrofluidics 13(2) doi:Artn 024101

10.1063/1.5070088

Ding C, Li YY, Guo FF, et al. (2016) A Cell-type-resolved Liver Proteome. Mol Cell Proteomics 15(10):3190-3202 doi:10.1074/mcp.M116.060145

Dixon LJ, Barnes M, Tang H, Pritchard MT, Nagy LE (2013) Kupffer cells in the liver. Compr Physiol 3(2):785-97 doi:10.1002/cphy.c120026

Duval K, Grover H, Han LH, et al. (2017) Modeling Physiological Events in 2D vs. 3D Cell Culture. Physiology 32(4):266-277 doi:10.1152/physiol.00036.2016

Guan Y, Enejder A, Wang MY, et al. (2021) A human multi-lineage hepatic organoid model for liver fibrosis. Nature Communications 12(1) doi:ARTN 6138

10.1038/s41467-021-26410-9

Guguen-Guillouzo C, Guillouzo A (2010) General Review on In Vitro Hepatocyte Models and Their Applications. Methods Mol Biol 640:1-40 doi:10.1007/978-1-60761-688-7_1

Harrison SP, Baumgarten SF, Verma R, Lunov O, Dejneka A, Sullivan GJ (2021) Liver Organoids: Recent Developments, Limitations and Potential. Front Med (Lausanne) 8:574047 doi:10.3389/fmed.2021.574047

Hong S, Song JM (2021) A 3D cell printing-fabricated HepG2 liver spheroid model for high-content in situ quantification of drug-induced liver toxicity. Biomater Sci 9(17):5939-5950 doi:10.1039/d1bm00749a

Ingelman-Sundberg M, Lauschke VM (2022) 3D human liver spheroids for translational pharmacology and toxicology. Basic Clin Pharmacol 130:5-15 doi:10.1111/bcpt.13587

Jang KJ, Otieno MA, Ronxhi J, et al. (2019) Reproducing human and cross-species drug toxicities using a Liver-Chip. Sci Transl Med 11(517) doi:ARTN eaax5516

10.1126/scitranslmed.aax5516

Kanebratt KP, Janefeldt A, Vilen L, et al. (2021) Primary Human Hepatocyte Spheroid Model as a 3D In Vitro Platform for Metabolism Studies. J Pharm Sci-Us 110(1):422-431 doi:10.1016/j.xphs.2020.10.043

Knolle P, Schlaak J, Uhrig A, Kempf P, Meyer zum Buschenfelde KH, Gerken G (1995) Human Kupffer cells secrete IL-10 in response to lipopolysaccharide (LPS) challenge. J Hepatol 22(2):226-9 doi:10.1016/0168-8278(95)80433-1

Koui Y, Himeno M, Mori Y, et al. (2021) Development of human iPSC-derived quiescent hepatic stellate cell-like cells for drug discovery and in vitro disease modeling. Stem Cell Rep 16(12):3050-3063 doi:10.1016/j.stemcr.2021.11.002

Koui Y, Kido T, Ito T, et al. (2017) An In Vitro Human Liver Model by iPSC-Derived Parenchymal and Non-parenchymal Cells. Stem Cell Rep 9(2):490-498 doi:10.1016/j.stemcr.2017.06.010

Kulsharova G, Kurmangaliyeva A (2021) Liver microphysiological platforms for drug metabolism applications. Cell Proliferat 54(9) doi:ARTN e13099

10.1111/cpr.13099

Kulsharova G, Kurmangaliyeva A, Darbayeva E, Rojas-Solorzano L, Toxeitova G (2021) Development of a Hybrid Polymer-Based Microfluidic Platform for Culturing Hepatocytes towards Liver-on-a-Chip Applications. Polymers-Basel 13(19) doi:ARTN 3215

10.3390/polym13193215

Landry J, Bernier D, Ouellet C, Goyette R, Marceau N (1985) Spheroidal Aggregate Culture of Rat-Liver Cells - Histotypic Reorganization, Biomatrix Deposition, and Maintenance of Functional Activities. J Cell Biol 101(3):914-923 doi:DOI 10.1083/jcb.101.3.914

Lee-Montiel FT, Laemmle A, Charwat V, et al. (2021) Integrated Isogenic Human Induced Pluripotent Stem Cell-Based Liver and Heart Microphysiological Systems Predict Unsafe Drug-Drug Interaction. Front Pharmacol 12:667010 doi:10.3389/fphar.2021.667010

Liu Y, Sheng JY, Ding J, Chan YS (2023) A decade of liver organoids: Advancements in disease modeling. Clin Mol Hepatol doi:10.3350/cmh.2022.0428

Lohasz C, Bonanini F, Hoelting L, Renggli K, Frey O, Hierlemann A (2020) Predicting Metabolism-Related Drug-Drug Interactions Using a Microphysiological Multitissue System. Adv Biosyst 4(11) doi:ARTN 2000079

10.1002/adbi.202000079

Lu SM, Cuzzucoli F, Jiang J, et al. (2018) Development of a biomimetic liver tumor-on-a-chip model based on decellularized liver matrix for toxicity testing. Lab Chip 18(22) doi:10.1039/c8lc00852c

Luckey SW, Taylor M, Sampey BP, Scheinman RI, Petersen DR (2002) 4-hydroxynonenal decreases interleukin-6 expression and protein production in primary rat Kupffer cells by inhibiting nuclear factor-kappa B activation. J Pharmacol Exp Ther 302(1):296-303 doi:UNSP 33522/990833

10.1124/jpet.102.033522

Maepa SW, Ndlovu H (2020) Advances in generating liver cells from pluripotent stem cells as a tool for modeling liver diseases. Stem Cells 38(5):606-612 doi:10.1002/stem.3154

Mannaerts I, Eysackers N, Anne van Os E, et al. (2020) The fibrotic response of primary liver spheroids recapitulates in vivo hepatic stellate cell activation. Biomaterials 261:120335 doi:10.1016/j.biomaterials.2020.120335

Messner CJ, Schmidt S, Ozkul D, et al. (2021) Identification of miR-199a-5p, miR-214-3p and miR-99b-5p as Fibrosis-Specific Extracellular Biomarkers and Promoters of HSC Activation. Int J Mol Sci 22(18) doi:ARTN 9799

10.3390/ijms22189799

Moradi E, Jalili-Firoozinezhad S, Solati-Hashjin M (2020) Microfluidic organ-on-a-chip models of human liver tissue. Acta Biomaterialia 116:67-83 doi:10.1016/j.actbio.2020.08.041

Olgasi C, Cucci A, Follenzi A (2020) iPSC-Derived Liver Organoids: A Journey from Drug Screening, to Disease Modeling, Arriving to Regenerative Medicine. Int J Mol Sci 21(17) doi:ARTN 6215

10.3390/ijms21176215

Ouchi R, Togo S, Kimura M, et al. (2019) Modeling Steatohepatitis in Humans with Pluripotent Stem Cell-Derived Organoids. Cell Metab 30(2):374-+ doi:10.1016/j.cmet.2019.05.007

Palakkan AA, Drummond R, Anderson RA, et al. (2015) Polarisation and functional characterisation of hepatocytes derived from human embryonic and mesenchymal stem cells. Biomed Rep 3(5):626-636 doi:10.3892/br.2015.480

Pelletier RD, Lai WG, Wong YN (2013) Application of a substrate cocktail approach in the assessment of cytochrome P450 induction using cultured human hepatocytes. J Biomol Screen 18(2):199-210 doi:10.1177/1087057112463732

Ribeiro AJS, Yang XN, Patel V, Madabushi R, Strauss DG (2019) Liver Microphysiological Systems for Predicting and Evaluating Drug Effects. Clin Pharmacol Ther 106(1):139-147 doi:10.1002/cpt.1458

Riede J, Wollmann BM, Molden E, Ingelman-Sundberg M (2021) Primary Human Hepatocyte Spheroids as an In Vitro Tool for Investigating Drug Compounds with Low Hepatic Clearance. Drug Metab Dispos 49(7):501-508 doi:10.1124/dmd.120.000340

Ryu NE, Lee SH, Park H (2019) Spheroid Culture System Methods and Applications for Mesenchymal Stem Cells. Cells 8(12) doi:10.3390/cells8121620

Skardal A, Murphy SV, Devarasetty M, et al. (2017) Multi-tissue interactions in an integrated three-tissue organ-on-a-chip platform. Sci Rep 7(1):8837 doi:10.1038/s41598-017-08879-x

Takebe T, Zhang RR, Koike H, et al. (2014) Generation of a vascularized and functional human liver from an iPSC-derived organ bud transplant. Nat Protoc 9(2):396-409 doi:10.1038/nprot.2014.020

Tanimizu N, Ichinohe N, Sasaki Y, et al. (2021) Generation of functional liver organoids on combining hepatocytes and cholangiocytes with hepatobiliary connections ex vivo. Nat Commun 12(1):3390 doi:10.1038/s41467-021-23575-1

Tasnim F, Xing JW, Huang XZ, et al. (2019) Generation of mature kupffer cells from human induced pluripotent stem cells. Biomaterials 192:377-391 doi:10.1016/j.biomaterials.2018.11.016

Thompson WL, Takebe T (2020) Generation of multi-cellular human liver organoids from pluripotent stem cells. Methods Cell Biol 159:47-68 doi:10.1016/bs.mcb.2020.03.009

Vilstrup H (1980) Synthesis of urea after stimulation with amino acids: relation to liver function. Gut 21(11):990-5 doi:10.1136/gut.21.11.990

Vinken M, Hengstler JG (2018) Characterization of hepatocyte-based in vitro systems for reliable toxicity testing. Arch Toxicol 92(10):2981-2986 doi:10.1007/s00204-018-2297-6

Williams DP (2018) Application of hepatocyte-like cells to enhance hepatic safety risk assessment in drug discovery. Philos Trans R Soc Lond B Biol Sci 373(1750) doi:10.1098/rstb.2017.0228

Wu FF, Wu D, Ren Y, et al. (2019) Generation of hepatobiliary organoids from human induced pluripotent stem cells. J Hepatol 70(6):1145-1158 doi:10.1016/j.jhep.2018.12.028

Xu QQ (2021) Human Three-Dimensional Hepatic Models: Cell Type Variety and Corresponding Applications. Front Bioeng Biotech 9 doi:ARTN 730008

10.3389/fbioe.2021.730008

Yin FC, Zhang X, Wang L, et al. (2021) HiPSC-derived multi-organoids-on-chip system for safety assessment of antidepressant drugs. Lab Chip 21(3):571-581 doi:10.1039/d0lc00921k

Zisser A, Ipsen DH, Tveden-Nyborg P (2021) Hepatic Stellate Cell Activation and Inactivation in NASH-Fibrosis-Roles as Putative Treatment Targets? Biomedicines 9(4) doi:10.3390/biomedicines9040365

Supplementary material 3

Kidney organoids, tubuloids, and OoC

Aspects making kidney MPS unique

Kidneys are vital organs that play a variety of different functions, the most critical being the generation of urine to eliminate extraneous or potentially toxic molecules from the body. The major tissue subunit of the kidneys is the nephron, a microfluidic structure that filters blood and processes the filtrate through tiny tubules to form the urine. For many years, modeling the structure and function of the nephron was beyond our reach in cell culture. Today, however, the field of kidney organoids and MPS is burgeoning, raising new possibilities for studying physiology and disease.

Status of kidney MPS development and examples of purposes of use

Nephrons form only during embryonic development. Breakthroughs in PSC differentiation first enabled the derivation of human kidney organoids that resemble primitive nephrons (Freedman *et al*, 2015; Morizane *et al*, 2015; Taguchi *et al*, 2014; Takasato *et al*, 2015). The hallmark of these structures is the presence of podocytes (filtering cells), proximal tubules, and distal tubules in contiguous segments along a proximal-to-distal axis that resembles the proximal end of the nephron. Collecting duct-like structures, which have features of the ducts that drain and concentrate the urine, can also be generated, using separate protocols for differentiation (Mae *et al*, 2018; Taguchi & Nishinakamura, 2017; Zeng *et al*, 2021). The ureteric-like structures can be co-cultured with proximal nephron-like kidney organoids to produce more sophisticated, connected architectures (Tanigawa *et al*, 2022).

In parallel with these approaches using PSCs, the use of human cell lines such as ciPTECs and RPTEC/TERT1 and advances in primary cell culture have also made great progress towards 3D devices, particularly for proximal tubular epithelial cells (PTEC). Several different forms of kidney-on-chip have been described that share the common feature of enabling microfluidic perfusion through a bioengineered tubular structure. In addition, culture conditions that enable the continued passaging and expansion of tubular cells in 3D matrix have been developed, the so-called tubuloid cultures. OoC have certain advantages over organoids and *vice versa* (table S2).

	**TABLE S2. Advantages of kidney MPS and organoid systems**
**PTEC OoC**	● Engineered microchips with primary adult human kidney cells in tubular geometry<br>● Perfusable - recapitulates fluid shear stress and mechanical strain observed in vivo<br>● Can model several kidney tubular functions (transport, metabolism, injury)<br>● Media to cell ratios (flow rates) approximate physiological values<br>● Under consideration by pharmaceutical industry and regulatory agencies to enable/guide clinical trials.
**iPS ORGANOID**	● Derived from human iPS cells with potential to form whole kidneys & full complement of cell types<br>● iPSC lines are permanent & gene editable to produce powerful models of disease or physiology reporters<br>● Can be generated in high-throughput formats for dose-dependent therapeutic discovery and testing<br>● Contiguous nephron segments enable simultaneous assessment of diverse cell types and their interactions<br>● Able to respond to stress by expressing and/or releasing injury markers in specific cell types

The aforementioned advances in human PSC differentiation as well as renal cell primary culture toproduce organoids, OoC, and tubuloids, enable the development of more accurate and relevant models of physiology, injury/repair, and even genetic diseases. Given the complexity of the kidneys, this is undoubtedly a strength, with examples of modeling phenotypic states such as acute kidney injury after cisplatin treatment, glomerulosclerosis, cystogenesis in polycystic kidney disease, and infection by SARS-CoV-2 coronavirus, all of which are challenging to reproduce in 2D culture models (Cruz *et al*, 2017; Freedman *et al.*, 2015; Garreta *et al*, 2022; Helms *et al*, 2021; Kim *et al*, 2017). Considering the aforementioned advantages, kidney organoids and MPS represent more reliable tools than animal models to mimic the complexity of the kidney and renal disease as it occurs in vivo. in general, animal models do not adequately recapitulate renal disease, differ in genome sequence from humans, and may lack appropriate endogenous therapeutic targets such as apolipoprotein L1 (Liu *et al*, 2020).

Aspects of kidney MPS quality control

Despite these advantages, the novelty, complexity, and limited availability of these human kidney models, their lack of standardization, and questions regarding their appropriateness and clinically-relevant interpretation, have all hindered their widespread adoption and application. With regard to standardization, it may be easier to define structural and functional hallmarks than to agree upon a common differentiation strategy or culture device. This is a logical strategy considering that the conditions that are required to model particular processes or phenotypes have not been completely understood. For instance, in kidney organoids, mutations in *PKD1* or *PKD2* are sufficient to produce a specific cystic phenotype reminiscent of polycystic kidney disease, but only using certain protocols that produce smaller organoids (Cruz *et al.*, 2017; Freedman *et al.*, 2015; Tran *et al*, 2022). Thus, it may be beneficial at this stage to cast a wider net, with certain protocols being better fit for certain purposes. Nevertheless, there are certainly structural and functional features that should be demonstrated for any given model, and it is important to agree upon such criteria.

Physiology and phenotype depend greatly on microenvironment and culture conditions, but organoid protocols may lack salient details or appropriate samples of reagents, which can vary by manufacturer or batch. It is critical to establish detailed protocols and vetted samples of key reagents for (a) organoid differentiation, including metanephric (Freedman *et al.*, 2015; Morizane *et al.*, 2015; Taguchi *et al.*, 2014; Takasato *et al.*, 2015), ureteric (Mae *et al.*, 2018; Taguchi & Nishinakamura, 2017; Zeng *et al.*, 2021), and stromal (Tanigawa *et al.*, 2022); (b) healthy physiology, including absorption of fluorescent dextran, glucose, and methotrexate into tubules, and swelling in response to forskolin (Freedman, 2022; Freedman *et al.*, 2015; Li *et al*, 2022; Low *et al*, 2019; Takasato *et al.*, 2015; Weber *et al*, 2016); (c) injury and acquired disease, including SARS-CoV-2 nephropathy (Helms *et al.*, 2021; Jansen *et al*, 2022; Monteil *et al*, 2020) and acute kidney injury (Freedman *et al.*, 2015; Imaoka *et al*, 2020; Morizane *et al.*, 2015; Takasato *et al.*, 2015); and (c) genetic diseases, encompassing phenotypic assays and microenvironment specific for iPSC lines described above.

One aspect of kidney function that requires special attention is the production of filtered urine. This is one of the major biological functions of the kidneys, and an important clinical metric, estimated based upon glomerular filtration rate. However, excretion and reabsorption are key function of the tubules. To date, kidney organoids *in vitro* do not exhibit true vascularization to produce filtering glomeruli in

a petri dish or chip. When organoids are implanted beneath the kidney capsule, however, they show a remarkable ability to generate glomerulus-like structures (Freedman & Dekel, 2023; Gupta *et al*, 2019; Nam *et al*, 2019; Sharmin *et al*, 2016; van den Berg *et al*, 2018). It is currently unclear whether these can produce urine, although a degree of size-selective filtration appears to be possible within the grafts. Further work is required to assess glomerular filtration rate more carefully in these structures, which could be an important parameter for comparison between organoid models and the clinic. Significant effort is required to further reconstitute this clinically relevant function in a microfluidic model *in vivo.*

As the field matures, it is anticipated that there will ultimately be a convergence of these various cell culture models. One can already see some of this taking place. Two recent papers describe the use of kidney organoids in microfluidic devices to model polycystic kidney disease phenotypes under flow (Hiratsuka et al, 2022; Li et al., 2022). Although these systems remain limited and lack true directional perfusion of fluid through kidney tubules, they nevertheless improve upon the physiological relevance of static organoid cultures, merging these with MPS. It is similarly possible to envision connections forming between organoids and tubuloids, or tubuloids and MPS. As major functional features are reconstituted, there will be a movement towards such models, ultimately converging on organotypic cultures that approximate the architecture and function of bona fide kidneys.

The following table S3 summarizes parameters to characterize different iPSC-derived mature kidney types in a MPS and could be used as QC.

Table S3. Recommendation of quality control for kidney Microphysiological Systems (MPS).

**The general features of the MPS/organoid**	**Key morphological, biochemical, and functional parameters (or endpoints) to characterize the model**	**Exemplar analytical methods**	**Acceptance criteria**	**References**
Nephron formation	Morphology	Microscopy (immunofluorescence) at low and high magnifications	Podocytes, proximal tubules, and distal tubules in contiguous segments	(Freedman *et al.*, 2015; Morizane *et al.*, 2015; Taguchi *et al.*, 2014; Takasato *et al.*, 2015)

Cargo accumulation & transport	Function	Microscopy (brightfield & fluorescence) at low and high magnifications	Forskolin-induced tubular swelling; dextran uptake inside tubules; glucose uptake	(Freedman, 2022; Freedman *et al.*, 2015; Li *et al.*, 2022; Low *et al.*, 2019; Takasato *et al.*, 2015; Weber *et al.*, 2016)
Glomerulus formation	Morphology	Microscopy (immunofluorescence & transmission electron) at low and high magnifications	Invasion of podocyte clusters by capillaries to form joint basement membranes	(Freedman & Dekel, 2023; Gupta *et al.*, 2019; Nam *et al.*, 2019; Sharmin *et al.*, 2016; van
			tubules treated with cisplatin	den Berg *et al.*, 2018) *al.*, 2020; Morizane *et*
Filtration	Function	Microscopy (fluorescence)	exclusion of red blood cells; size-selective permeability to	(Freedman *et al.*, 2015; Low *et al.*, 2019; *al.*, 2015; Takasato *et al.*, 2015)
Genetic disease	Morphology	CRISPR gene editing, microscopy, quantification	dextrans Emergence of cysts from tubules	Takasato *et al.*, 2015) (Cruz *et al.*, 2017;
Acute tubular necrosis	Function	Microscopy (immunofluorescence) & biochemistry (qPCR)	KIM-1 expression in proximal in *PKD1* or *PKD2* null organoids, but not in isogenic	(Freedman *et al.*, 2015; Imaoka *et al.*, (Freedman *et al.*, 2015; Tran *et al.*,
			controls	2022)
Infection	Morphology & Biochemistry	Microscopy (fluorescence), qPCR, plaque assays	Selective SARS-CoV-2 propagation in proximal tubular epithelial cells	(Helms *et al.*, 2021; Jansen *et al.*, 2022; Monteil *et al.*, 2020)

References

Cruz NM, Song X, Czerniecki SM, Gulieva RE, Churchill AJ, Kim YK, Winston K, Tran LM, Diaz MA, Fu H et al (2017) Organoid cystogenesis reveals a critical role of microenvironment in human polycystic kidney disease. Nat Mater 16: 1112-1119

Freedman BS (2022) Physiology assays in human kidney organoids. Am J Physiol Renal Physiol 322: F625-F638

Freedman BS, Brooks CR, Lam AQ, Fu H, Morizane R, Agrawal V, Saad AF, Li MK, Hughes MR, Werff RV et al (2015) Modelling kidney disease with CRISPR-mutant kidney organoids derived from human pluripotent epiblast spheroids. Nat Commun 6: 8715

Freedman BS, Dekel B (2023) Engraftment of Kidney Organoids In Vivo. Curr Transplant Rep 10: 29-39

Garreta E, Prado P, Stanifer ML, Monteil V, Marco A, Ullate-Agote A, Moya-Rull D, Vilas-Zornoza A, Tarantino C, Romero JP et al (2022) A diabetic milieu increases ACE2 expression and cellular susceptibility t o SARS-CoV-2 infections in human kidney organoids and patient cells. Cell Metab 34: 857-873 e859

Gupta AK, Coburn JM, Davis-Knowlton J, Kimmerling E, Kaplan DL, Oxburgh L (2019) Scaffolding kidney organoids on silk. J Tissue Eng Regen Med 13: 812-822

Helms L, Marchiano S, Stanaway IB, Hsiang TY, Juliar BA, Saini S, Zhao YT, Khanna A, Menon R, Alakwaa F et al (2021) Cross-validation of SARS-CoV-2 responses in kidney organoids and clinical populations. JCI Insight 6

Hiratsuka K, Miyoshi T, Kroll KT, Gupta NR, Valerius MT, Ferrante T, Yamashita M, Lewis JA, Morizane R (2022) Organoid-on-a-chip model of human ARPKD reveals mechanosensing pathomechanisms for drug discovery. Sci Adv 8: eabq0866

Imaoka T, Yang J, Wang L, McDonald MG, Afsharinejad Z, Bammler TK, Van Ness K, Yeung CK, Rettie AE, Himmelfarb J et al (2020) Microphysiological system modeling of ochratoxin A-associated nephrotoxicity. Toxicology 444: 152582

Jansen J, Reimer KC, Nagai JS, Varghese FS, Overheul GJ, de Beer M, Roverts R, Daviran D, Fermin LAS, Willemsen B et al (2022) SARS-CoV-2 infects the human kidney and drives fibrosis in kidney organoids. Cell Stem Cell 29: 217-231 e218

Kim YK, Refaeli I, Brooks CR, Jing P, Gulieva RE, Hughes MR, Cruz NM, Liu Y, Churchill AJ, Wang Y et al (2017) Gene-Edited Human Kidney Organoids Reveal Mechanisms of Disease in Podocyte Development. Stem Cells 35: 2366-2378

Li SR, Gulieva RE, Helms L, Cruz NM, Vincent T, Fu H, Himmelfarb J, Freedman BS (2022) Glucose absorption drives cystogenesis in a human organoid-on-chip model of polycystic kidney disease. Nat Commun 13: 7918

Liu E, Radmanesh B, Chung BH, Donnan MD, Yi D, Dadi A, Smith KD, Himmelfarb J, Li M, Freedman BS et al (2020) Profiling APOL1 Nephropathy Risk Variants in Genome-Edited Kidney Organoids with Single-Cell Transcriptomics. Kidney360 1: 203-215

Low JH, Li P, Chew EGY, Zhou B, Suzuki K, Zhang T, Lian MM, Liu M, Aizawa E, Rodriguez Esteban C et al (2019) Generation of Human PSC-Derived Kidney Organoids with Patterned Nephron Segments and a De Novo Vascular Network. Cell Stem Cell 25: 373-387 e379

Mae SI, Ryosaka M, Toyoda T, Matsuse K, Oshima Y, Tsujimoto H, Okumura S, Shibasaki A, Osafune K (2018) Generation of branching ureteric bud tissues from human pluripotent stem cells. Biochem Biophys Res Commun 495: 954-961

Monteil V, Kwon H, Prado P, Hagelkruys A, Wimmer RA, Stahl M, Leopoldi A, Garreta E, Hurtado Del Pozo C, Prosper F et al (2020) Inhibition of SARS-CoV-2 Infections in Engineered Human Tissues Using Clinical-Grade Soluble Human ACE2. Cell 181: 905-913 e907

Morizane R, Lam AQ, Freedman BS, Kishi S, Valerius MT, Bonventre JV (2015) Nephron organoids derived from human pluripotent stem cells model kidney development and injury. Nat Biotechnol 33: 1193-1200

Nam SA, Seo E, Kim JW, Kim HW, Kim HL, Kim K, Kim TM, Ju JH, Gomez IG, Uchimura K et al (2019) Graft immaturity and safety concerns in transplanted human kidney organoids. Exp Mol Med 51: 1-13

Sharmin S, Taguchi A, Kaku Y, Yoshimura Y, Ohmori T, Sakuma T, Mukoyama M, Yamamoto T, Kurihara H, Nishinakamura R (2016) Human Induced Pluripotent Stem Cell-Derived Podocytes Mature into Vascularized Glomeruli upon Experimental Transplantation. J Am Soc Nephrol 27: 1778-1791

Taguchi A, Kaku Y, Ohmori T, Sharmin S, Ogawa M, Sasaki H, Nishinakamura R (2014) Redefining the in vivo origin of metanephric nephron progenitors enables generation of complex kidney structures from pluripotent stem cells. Cell Stem Cell 14: 53-67

Taguchi A, Nishinakamura R (2017) Higher-Order Kidney Organogenesis from Pluripotent Stem Cells. Cell Stem Cell 21: 730-746 e736

Takasato M, Er PX, Chiu HS, Maier B, Baillie GJ, Ferguson C, Parton RG, Wolvetang EJ, Roost MS, Chuva de Sousa Lopes SM et al (2015) Kidney organoids from human iPS cells contain multiple lineages and model human nephrogenesis. Nature 526: 564-568

Tanigawa S, Tanaka E, Miike K, Ohmori T, Inoue D, Cai CL, Taguchi A, Kobayashi A, Nishinakamura R (2022) Generation of the organotypic kidney structure by integrating pluripotent stem cell-derived renal stroma. Nat Commun 13: 611

Tran T, Song CJ, Nguyen T, Cheng SY, McMahon JA, Yang R, Guo Q, Der B, Lindstrom NO, Lin DC et al (2022) A scalable organoid model of human autosomal dominant polycystic kidney disease for disease mechanism and drug discovery. Cell Stem Cell 29: 1083-1101 e1087

van den Berg CW, Ritsma L, Avramut MC, Wiersma LE, van den Berg BM, Leuning DG, Lievers E, Koning M, Vanslambrouck JM, Koster AJ et al (2018) Renal Subcapsular Transplantation of PSC- Derived Kidney Organoids Induces Neo-vasculogenesis and Significant Glomerular and Tubular Maturation In Vivo. Stem Cell Rep 10: 751-765

Weber EJ, Chapron A, Chapron BD, Voellinger JL, Lidberg KA, Yeung CK, Wang Z, Yamaura Y, Hailey DW, Neumann T et al (2016) Development of a microphysiological model of human kidney proximal tubule function. Kidney Int 90: 627-637

Zeng Z, Huang B, Parvez RK, Li Y, Chen J, Vonk AC, Thornton ME, Patel T, Rutledge EA, Kim AD et al (2021) Generation of patterned kidney organoids that recapitulate the adult kidney collecting duct system from expandable ureteric bud progenitors. Nat Commun 12: 3641

Supplementary material 4

Organ specific quality control for Gut MPS

Aspects making gut MPS unique

The gut, also known as the gastrointestinal tract (GI tract), is responsible for the digestion and absorption of food and the elimination of waste products. The GI tract collectively is composed of several organs: including the mouth, oesophagus, stomach, small intestine, large intestine, rectum, and anus. Each of these organs have a unique function in the digestive process but in this manuscript, we will be focusing on emulating the function of small and large intestine *in vitro*.

The small intestine is the longest section of the digestive tract, and is divided into three parts: the duodenum, jejunum, and ileum. The inner lining of the small intestine has several key features that allow for efficient nutrient absorption (Kim et al. 2020; Mahadevan and 2017):

- Villi and Microvilli: The inner surface of the small intestine is lined with finger-like projections called villi, which are covered with even smaller projections called microvilli. These structures increase the surface area of the intestine, allowing for more efficient absorption of nutrients.
- Intestinal Glands: The lining of the small intestine also contains small pits or glands, which produce enzymes that help to digest food.
- Mucus-secreting Cells: These cells produce mucus that helps to lubricate the intestine and protect the lining from harmful substances.
- Endocrine and Immune Cells: The small intestine contains specialized cells that produce hormones and immune cells that help to regulate digestion and protect against harmful pathogens.

The colon, or large intestine, is responsible for absorbing water and electrolytes from the undigested material that passes through it. Due to this functional difference, there are also some anatomical differences between small and large intestine. Unlike the small intestine, the colon has a relatively smooth surface, and it lacks villi and microvilli as the water absorption does not require the same amount of surface, which the villi and microvilli contribute. Instead, colon has the following key features and cell types(Clevers 2013):

- Crypts of Lieberkühn: These are small pits or glands in the lining of the colon that secrete mucus and other substances.
- Absorptive and Secretory Cells: The lining of the colon contains cells that absorb water and electrolytes, as well as cells that secrete mucus and other substances.
- Goblet Cells: These cells secrete mucus that helps to lubricate the colon and protect the lining from harmful substances.

- Immune Cells: The colon also contains immune cells that help to protect against harmful pathogens.

Status of gut MPS development and examples of purposes of use

As seen above, gut physiology is highly complex and simple well-plate based *in vitro* models have limited capacity to recapitulate this physiology. Therefore, gut MPS, also known as complex GI models, are *in vitro* models that simulate the structure as described above and the function of the human gut in a controlled environment that is physiologically relevant. These models have been used in drug discovery and development for many years in varying complexities, highlighting their importance in the preclinical drug development (Fedi et al. 2021). Here, we will describe two most common forms of gut MPS (gut organoids and gut-on-chip).

Gut organoids are 3D structures that are grown *in vitro* from adult tissue stem cells and can generate the range of the native cell types *in situ* and mimic the architecture and function of the human gut (Clevers 2013). Complexity of gut organoid models can be increased stepwise by adding components of the microenvironment such as microbiota (Bozzetti and Senger 2022; Min et al. 2020; Puschhof et al. 2021a; Puschhof et al. 2021b). These organoid-microbiota cultures have also shown great potential in terms of capturing disease pathology (Gunther et al. 2022; Poletti et al. 2021). It is also possible to use these models in a drug screening setting at the early discovery phase for target identification via genetic manipulation like CRISPR (Ringel et al. 2020) or to understand specific disease mechanisms (Gunther et al. 2022; Poletti et al. 2021). Besides these very attractive advantages of gut organoids, there are also some considerable drawbacks such as to the inability to capture the physiological gut architecture, considering e.g., epithelial polarity, but also the physiologically relevant interactions occurring between cells and microenvironment components (immune cells and microbiota) as listed in Table S4.

	**Table S4. Advantages and disadvantages of gut OoC and organoid systems**	
	**Advantages**	**Disadvantages**
**Gut Organ-on-Chip**	● <u>Mimics human physiology:</u><br>○ Correct physiology and polarisation of epithelial barrier of the gut can be achieved through right geometry of the microfluidic chips.	● <u>Limitations in complexity:</u> Although gut-on-chip technology can replicate some aspects of gut physiology, it cannot yet fully mimic the complexity of the human gut. This may limit the usefulness of the technology for studying certain diseases or physiological processes.

	○ Recapitulates relevant fluid shear stress and mechanical strain observed *in vivo*.<br>○ Translational value can be improved by adding immune cells (resident of circulating) and microbiota.<br>● Facilitates personalized medicine: The gut-on-chip technology can be used to create personalized models of the gut with specific phenotype/genotype, allowing scientists to evaluate individual, patients' specific response to treatments.<br>● Provides new insights into gut diseases: The gut-on-chip technology can help researchers to better understand the underlying mechanisms of gut diseases, such as inflammatory bowel disease (IBD) or irritable bowel syndrome (IBS), which can be difficult to study *in vivo*.<br>● Reduces animal testing: By using the gut-on-chip technology to test drugs, researchers can reduce their reliance on animal models, which is expensive, time-consuming, and ethically controversial.	● Difficulty in reproducing results: The gut-on-chip technology can be difficult to reproduce consistently, which can make it challenging to compare results across different studies or labs.<br>● High cost: The development and use of gut-on-chip technology can be resource intensive and expensive, which may limit its accessibility to researchers with limited funding or resources.<br>● Lack of standardization: There is currently a lack of standardized protocols for using gut-on-chip technology, which can make it difficult to compare results or replicate studies across different labs.<br>● Low-throughput: testing large number of compounds (for toxicity testing) or potential drugs or therapeutic agents may not be possible.
**Gut Organoids**	● Enables high-throughput drug screening: The gut organoids can be used to rapidly screen large numbers of potential drugs or therapeutic agents, allowing researchers to identify promising candidates more quickly and efficiently than with traditional animal or *in vitro* models.<br>● Reproducibility: Gut organoids can be easily generated in large numbers in controlled environments and can be used to study a wide range of biological processes. This allows for reproducible experiments and high-throughput screening of drugs.	● Heterogeneity: Gut organoids are not uniform and can vary in size, shape, and cellular composition. This heterogeneity can make it difficult to compare results across different experiments or laboratories.<br>● Limited complexity: While gut organoids are more complex than traditional cell culture systems, they still lack some of the complexity of the human gut. This may limit their usefulness for studying certain diseases or physiological processes.<br>● Limited differentiation potential: While gut organoids can differentiate into

	• <u>Mimic gut architecture:</u> Gut organoids are more complex than traditional 2D cell cultures and better mimic the architecture of the human gut. They differentiate *in situ* to multiple cell types, which allows for the study of interactions between different cell types. It is also possible to add microbiome and immune cells to increase complexity.<br><br>• <u>Disease modelling:</u> Gut organoids can be used to model diseases, such as IBD and colorectal cancer, by introducing specific genetic mutations or environmental factors.<br><br>• <u>Personalized medicine:</u> Gut organoids can be generated from patient-specific stem cells, allowing for personalized medicine approaches that take into account individual differences in drug responses.<br><br>• <u>Ethical considerations:</u> The use of gut organoids can reduce the need for animal models in research.	many different cell types, they do not fully recapitulate the diversity of cell types found in the gut epithelium and may not accurately represent the spatial distribution and differentiation status of cells *in vivo* (Hofer and Lutolf 2021) Click or tap here to enter text.<br><br>• <u>Lack of standardization:</u> There is currently a lack of standardized protocols for generating and characterizing gut organoids, which can make it difficult to compare results or replicate studies across different labs.<br><br>• <u>Short lifespan:</u> Gut organoids have a limited lifespan and can become less representative of the human gut over time. This may require frequent generation of new organoids or careful monitoring of organoid quality.<br><br>• <u>Cost:</u> The generation and maintenance of gut organoids can be expensive, which may limit their accessibility to researchers with limited funding or resources.<br><br>• <u>Limits in capturing the gut architecture:</u><br><br>o <u>Lack of access to the basolateral side:</u> In gut organoids, the basolateral side of the epithelial cells is typically inaccessible, making it difficult to study the interactions between the epithelium and the underlying stromal cells, immune cells, and extracellular matrix. This limits the ability to study the complex intercellular signalling that occurs in the gut.<br><br>o <u>Potential for altered polarity:</u> The polarity of gut organoids can be disrupted by a variety of factors, including culture conditions and genetic alterations. This can affect the function of the epithelium and

		limit the utility of the organoids for studying normal gut physiology and disease.<br>o Microbiome or immune cell addition is possible but lacks physiological relevance in terms of tissue architecture or physiological interaction

In terms of cell source, adult tissue-specific stem cells or iPSCs are two sources of cells that can be used to generate gut organoids. Both approaches have their own advantages and disadvantages. The choice between these two approaches depends on the specific research or therapeutic goals, available resources, and the desired characteristics of the gut organoids. Organoids from adult stem cells are easier to obtain as the tissue is readily harvested which increases the accessibility of this source. Originating from healthy, stable mature tissue, adult stem cell derived organoids are generally more genetically stable compared to iPSCs. They have undergone fewer genetic alterations and have a lower risk of genomic instability. Similarly, since they are sourced from adult tissue, the cells are already committed in the lineage and therefore they have a higher propensity to differentiate into the desired cell types. At the same time, this lineage commitment affects the proliferation capacity with limited passaging capability *in vitro* especially if obtained from older donors. On the other hand, iPSC-derived gut organoids can proliferate indefinitely in culture, allowing for the generation of large quantities of cells, but both the requirement of extensive reprogramming and their not fully differentiated nature combined with proliferative capacity can lead to genetic abnormalities more often than adult stem cell derived gut organoids (Lehmann et al. 2019).

On the other hand, more complex gut MPS, gut-on-chip technology, (Kim et al. 2012)has numerous advantages over traditional methods (including organoids) to study gut physiology . Gut-on-chip can better capture the right gut architecture and microenvironment, accounting for flow and shear stress as well as the stretch, right polarity of the endothelial cells, and enabling the possibility to add microbiota on the endothelial side and to introduce both resident and circulating immune cells in a physiologically faithful manner (Kim et al. 2012; Puschhof et al. 2021a). However, this increased complexity comes at the expense of the throughput, with gut-on-chip platforms having considerably lower throughput than organoids. Combined with the cost and the labour required to perform adequate number of experiments to answer the question at hand, the utility of the gut-on-chip at practical level is far from its potential.

From a complexity point of view, an ideal approach could be to use gut organoids on a gut-on-chip platform, accounting for donor specific microbiota, immune cells (such as PBMC derived) and donor specific endothelial cells to create patient-gut-on-chip for personalised medicine or a leap towards clinical trials-on-chip. However, before reaching that stage, there are still a series of technological that needs to be overcome (Table S4).

As mentioned before, *in vitro* gut models have been used in the drug development workflow for many decades where several steps in the process requires through checks such as drug absorption, metabolism, toxicity evaluation. Additionally, these models are frequently used in pharmacology and also as disease models to study various aspects of gut physiology, drug absorption, and the pathogenesis of gastrointestinal diseases.

Predicting drug absorption in the gut is very important as the primary site for drug absorption in the body necessitates the use of translational gut MPS models. These gut MPS can mimic the physiological conditions of the gut, including the presence of various tissue specific cell types, enzymes, and transporters, which play crucial roles in drug absorption. By using gut MPS, researchers can assess how drugs are absorbed across the gut barrier, predict their bioavailability, and optimize drug formulations to enhance drug absorption required to achieve the desired efficacy (Fedi et al. 2021; Youhanna and Lauschke 2021).

The gut is also a major site for drug metabolism after liver, where drugs can be broken down by enzymes produced by gut bacteria or enterocytes (cells lining the gut). Gut MPS assists understanding the absorption, distribution, metabolism, and excretion (ADME) of drugs in the gut and predict their metabolic fate in a human based platform, including the formation of active or toxic metabolites. This information is critical for optimizing drug candidates and avoiding potential adverse effects, especially where species differences of *in vivo* models can hamper the clinical translation (Davies et al. 2020).

In addition, MPS can be used to evaluate drug-drug interactions, which represents an important aspect in drug discovery. These interactions may occur when two or more drugs affect each other’s absorption, metabolism, or excretion in the gut. Drug-drug interactions can significantly impact the pharmacokinetics and efficacy of drugs, and gut MPS can provide valuable insights into these interactions in a human-based platform, helping researchers identify potential risks and optimize drug combinations (El Houari et al. 2022).

Toxicity evaluation is a critical component of the drug discovery process. During drug toxicity evaluation, several organs are typically checked for potential adverse effects. The organs checked may vary depending on the type of drug and the potential routes of administration, but some of the most commonly evaluated organs include liver, kidneys, lung, heart, central nervous systems and also GI tract. GI toxicity can cause symptoms such as nausea, vomiting, diarrhoea, or constipation, which can range from mild irritation to severe adverse effects. Most commonly used *in vitro* models available to evaluate GI toxicity are the Ussing chamber, organoid swelling, trans-well assays, explants and gut-on-chip (Youhanna and Lauschke 2021). Gut MPS can be used to assess these potential toxicity events, including evaluating their effects on gut barrier function, inflammation, and gut microbiota. This information can guide drug development and help identify drug candidates with better safety profiles (Peters et al. 2020).

In vitro gut MPS are also valuable tools for studying gastrointestinal diseases and their underlying mechanisms. Specific disease conditions can be replicated in the gut MPS, such IBD, colorectal cancer, or infectious diseases, by using cells or tissues derived from patients. These *in vitro* platforms mimic the disease environment in a human setting, and therefore allow investigating disease progression, immune responses, and the efficacy of potential treatments under settings that are more translational to the clinic (Kim et al. 2020; Morelli et al. 2023; Valiei et al. 2023; Xian et al. 2023).

Aspects of gut MPS quality control

By using highly complex gut-organoid-on-chip it is possible, for example, to evaluate drug metabolism or toxicity, although these phenomena may occur through interaction with other organs, which would require multi-organ-on-chips with a gut component. Multi-organ chips can mimic the complex physiological interactions between different organs in the body, enabling the evaluation of the linked reactions of drugs and other compounds that are metabolized and distributed throughout the body (Lee and Sung 2022; Picollet-D'hahan et al. 2021). The following Table S5 summarizes parameters to characterize different features of a gut MPS that can be used as QC.

**Table S5. Recommendation of quality control for gut Microphysiological Systems (MPS) – Organoids and Organ-on-Chip**				
**The general features of the MPS**	**Key morphological, biochemical, and functional parameters (for endpoints)**	**Exemplar analytical methods**	**Acceptance criteria**	**References**

	to characterize the model			
**Cell/tissue identity**	Tissue morphology	IHC/EM/H&E	• Presence of crypts and villi<br>• Organoid morphology (budding, cystic, compact etc.)	(Ashammakhi et al. 2020; Date and Sato 2015)
	Presence of key cell types through expression of relevant cellular markers	IF/IHC	• Tight junction proteins: ZO-1 and Claudin-4, F-actin, 38 occluding (internalised)<br>• Brush border enzymes: lactase, sucrase-isomaltase, and alkaline phosphatase<br>• Transmembrane mucins: MUC2 (most abundant, also goblet cell marker), MUC3A/B, MUC12, MUC13, MUC15, and MUC17,<br>• Cell polarisation: Villin, CEACAM1, Na-K-ATPase (basolateral side of enterocytes), SLC26A3 (apical ion channel)<br>• Gut cells:<br>○ Enteroendocrine cells: Chromogranin A<br>○ Enterocytes: Na-K-ATPase<br>○ Paneth cells: α-defensin	(Apostolou et al. 2021; Ashammakhi et al. 2020; Clevers 2013; Grondin et al. 2020; Maschmeyer et al. 2015; Nepal et al. 2019)

			○ Goblet cells: MUC2<br>○ Stem cell niche: Lgr5 and KI67<br>• Endothelial cells: vWF, CD31	
	Proliferation kinetics (for organoids)	LDH, MTT, CellTiterGlo, BrDU/EDU,<br>Alamar Blue,	• No change over time through multiple passages	(Xie and Wu 2016)
**Cell/Tissue function**	Tight barrier	TEER, IF	• Passive intestinal barrier permeability<br>○ Fluorescent dye leakage<br>• Active intestinal permeability:<br>○ Calcium absorption	(Bronner 2003)
	Drug metabolism	PCR, LC/MS (substrate metabolising)	• Drug metabolising enzymes: CYP34A (most abundant Phase 1 enzyme),<br>• Alpha-defensin (Drug metabolism, P-glycoprotein<br>• UGT1A1 (most abundant Phase 1 enzyme)	(Andersen and Sonne 2000; Ashammakhi et al. 2020; Fritz et al. 2019)
**Genomic characterisation**	Global and over time	RNA-seq (bulk or single cell)	• No genetic drift over passages<br>• Concordance with fresh primary tissue	
	Personalised	Whole genome sequencing (WGS)	• Individual genetic profiling (disease, donor specific)	

	Epigenetics	Histone methylation	• No genetic drift over passages (primary tissue concordance)<br>• Disease profiling	(Thalheim et al. 2021; Zhou et al. 2021)

References.

Andersen VC, Sonne J (2000) [Drug metabolism in the small intestine--the significance for biological availability]. Ugeskr Laeger 162(22):3215-9

Apostolou A, Panchakshari RA, Banerjee A, et al. (2021) A Novel Microphysiological Colon Platform to Decipher Mechanisms Driving Human Intestinal Permeability. Cell Mol Gastroenterol Hepatol 12(5):1719-1741 doi:10.1016/j.jcmgh.2021.07.004

Ashammakhi N, Nasiri R, Barros NR, et al. (2020) Gut-on-a-chip: Current progress and future opportunities. Biomaterials 255:120196 doi:10.1016/j.biomaterials.2020.120196

Bozzetti V, Senger S (2022) Organoid technologies for the study of intestinal microbiota-host interactions. Trends Mol Med 28(4):290-303 doi:10.1016/j.molmed.2022.02.001

Bronner F (2003) Mechanisms of intestinal calcium absorption. J Cell Biochem 88(2):387-393 doi:10.1002/jcb.10330

Clevers H (2013) The Intestinal Crypt, A Prototype Stem Cell Compartment. Cell 154(2):274-284 doi:10.1016/j.cell.2013.07.004

Date S, Sato T (2015) Mini-gut organoids: reconstitution of the stem cell niche. Annu Rev Cell Dev Biol 31:269-89 doi:10.1146/annurev-cellbio-100814-125218

Davies M, Peramuhendige P, King L, et al. (2020) Evaluation of In Vitro Models for Assessment of Human Intestinal Metabolism in Drug Discovery. Drug Metab Dispos 48(11):1169-1182 doi:10.1124/dmd.120.000111

El Houari A, Ecale F, Mercier A, et al. (2022) Development of an in vitro Model of Human Gut Microbiota for Screening the Reciprocal Interactions With Antibiotics, Drugs, and Xenobiotics. Front Microbiol 13:828359 doi:10.3389/fmicb.2022.828359

Fedi A, Vitale C, Ponschin G, Ayehunie S, Fato M, Scaglione S (2021) In vitro models replicating the human intestinal epithelium for absorption and metabolism studies: A systematic review. J Control Release 335:247-268 doi:10.1016/j.jconrel.2021.05.028

Fritz A, Busch D, Lapczuk J, Ostrowski M, Drozdzik M, Oswald S (2019) Expression of clinically relevant drug-metabolizing enzymes along the human intestine and their correlation to drug transporters and nuclear receptors: An intra-subject analysis. Basic Clin Pharmacol 124(3):245-255 doi:10.1111/bcpt.13137

Grondin JA, Kwon YH, Far PM, Haq S, Khan WI (2020) Mucins in Intestinal Mucosal Defense and Inflammation: Learning From Clinical and Experimental Studies. Front Immunol 11 doi:ARTN 2054

10.3389/fimmu.2020.02054

Gunther C, Winner B, Neurath MF, Stappenbeck TS (2022) Organoids in gastrointestinal diseases: from experimental models to clinical translation. Gut 71(9):1892-1908 doi:10.1136/gutjnl-2021-326560

Hofer M, Lutolf MP (2021) Engineering organoids. Nat Rev Mater 6(5):402-420 doi:10.1038/s41578-021-00279-y
Kim HJ, Huh D, Hamilton G, Ingber DE (2012) Human gut-on-a-chip inhabited by microbial flora that experiences intestinal peristalsis-like motions and flow. Lab Chip 12(12):2165-2174 doi:10.1039/c2lc40074j
Kim W, Kim GH, (2020) An intestinal model with a finger-like villus structure fabricated using a bioprinting process and collagen/SIS-based cell-laden bioink. vol 10, p 2495-2508
Lee HR, Sung JH (2022) Multiorgan-on-a-chip for realization of gut-skin axis. Biotechnol Bioeng 119(9):2590-2601 doi:10.1002/bit.28164
Lehmann R, Lee CM, Shugart EC, et al. (2019) Human organoids: a new dimension in cell biology. Mol Biol Cell 30(10):1129-1137 doi:10.1091/mbc.E19-03-0135
Mahadevan V, (2017) Anatomy of the small intestine. vol 35. Elsevier, p 407-412
Maschmeyer I, Lorenz AK, Schimek K, et al. (2015) A four-organ-chip for interconnected long-term co-culture of human intestine, liver, skin and kidney equivalents. Lab Chip 15(12):2688-99 doi:10.1039/c5lc00392j
Min S, Kim S, Cho SW (2020) Gastrointestinal tract modeling using organoids engineered with cellular and microbiota niches. Exp Mol Med 52(2):227-237 doi:10.1038/s12276-020-0386-0
Morelli M, Kurek D, Ng CP, Queiroz K (2023) Gut-on-a-Chip Models: Current and Future Perspectives for Host-Microbial Interactions Research. Biomedicines 11(2) doi:10.3390/biomedicines11020619
Nepal N, Arthur S, Sundaram U (2019) Unique Regulation of Na-K-ATPase during Growth and Maturation of Intestinal Epithelial Cells. Cells 8(6) doi:10.3390/cells8060593
Peters MF, Choy AL, Pin C, et al. (2020) Developing in vitro assays to transform gastrointestinal safety assessment: potential for microphysiological systems. Lab Chip 20(7):1177-1190 doi:10.1039/c9lc01107b
Picollet-D'hahan N, Zuchowska A, Lemeunier I, Le Gac S (2021) Multiorgan-on-a-Chip: A Systemic Approach To Model and Decipher Inter-Organ Communication. Trends Biotechnol 39(8):788-810 doi:10.1016/j.tibtech.2020.11.014
Poletti M, Arnauts K, Ferrante M, Korcsmaros T (2021) Organoid-based Models to Study the Role of Host-microbiota Interactions in IBD. J Crohns Colitis 15(7):1222-1235 doi:10.1093/ecco-jcc/jjaa257
Puschhof J, Pleguezuelos-Manzano C, Clevers H (2021a) Organoids and organs-on-chips: Insights into human gut-microbe interactions. Cell Host Microbe 29(6):867-878 doi:10.1016/j.chom.2021.04.002
Puschhof J, Pleguezuelos-Manzano C, Martinez-Silgado A, et al. (2021b) Intestinal organoid cocultures with microbes. Nat Protoc 16(10):4633-4649 doi:10.1038/s41596-021-00589-z
Ringel T, Frey N, Ringnalda F, et al. (2020) Genome-Scale CRISPR Screening in Human Intestinal Organoids Identifies Drivers of TGF-beta Resistance. Cell Stem Cell 26(3):431-440 e8 doi:10.1016/j.stem.2020.02.007
Thalheim T, Siebert S, Quaas M, et al. (2021) Epigenetic Drifts during Long-Term Intestinal Organoid Culture. Cells 10(7) doi:10.3390/cells10071718
Valiei A, Aminian-Dehkordi J, Mofrad MRK (2023) Gut-on-a-chip models for dissecting the gut microbiology and physiology. APL Bioeng 7(1):011502 doi:10.1063/5.0126541
Xian C, Zhang J, Zhao S, Li XG (2023) Gut-on-a-chip for disease models. J Tissue Eng 14:20417314221149882 doi:10.1177/20417314221149882
Xie BY, Wu AW (2016) Organoid Culture of Isolated Cells from Patient-derived Tissues with Colorectal Cancer. Chin Med J (Engl) 129(20):2469-2475 doi:10.4103/0366-6999.191782
Youhanna S, Lauschke VM (2021) The Past, Present and Future of Intestinal In Vitro Cell Systems for Drug Absorption Studies. J Pharm Sci 110(1):50-65 doi:10.1016/j.xphs.2020.07.001
Zhou Z, Van der Jeught K, Fang Y, et al. (2021) An organoid-based screen for epigenetic inhibitors that stimulate antigen presentation and potentiate T-cell-mediated cytotoxicity. Nat Biomed Eng 5(11):1320-1335 doi:10.1038/s41551-021-00805-x

Supplementary material 5

Organ-specific quality control: Lung

Aspects making lung MPS unique

Lung is the first line of defense to pathogens, particulates in the air and for inhaled medicines where these foreign objects encounter the respiratory barrier of the lung. The lung has a complex architecture and is made up of more than 50 cell types (Travaglini et al. 2020). The generation of lung organoid or MPS models are typically from a specific region of the lung, such as the alveoli or bronchi.

Status of lung MPS development and examples of purposes of use

The most common lung *in vitro* models before lung organoids and MPS were developed are the static air-liquid interphase (ALI) models that use a trans-well format, where primary lung epithelial cells are placed on the top (apical side) of the trans-well. An ALI culture is made up of ciliated cells, goblet cells and progenitor cells that create a polarized heterogenous pseudostratified epithelium which retains epithelial ion transport properties, secretory and mucociliary host defense mechanisms. Barrier integrity and tight junction development can be determined using the measurement of transepithelial electrical resistance (TEER) or the movement of fluorescein isothiocyanate (FITC)-Dextran across the barrier. The ALI system can be used in a 96-well format and does lend itself for testing aerosolized drugs and the modeling of certain disease states by utilizing cells from asthma and chronic obstructive pulmonary disease (COPD) patients. Typically, the ALI system only accounts for the epithelial component but recently studies have started to use lung fibroblasts. However, they still lack many key features that are important for lung function, such as endothelium, perfusion, breathing motion and the movement of immune cells under fluid flow. However, many of the new lung MPS models are an advancement of the ALI trans-well system (Bluhmki et al. 2020; Maurer et al. 2023; Michi and Proud 2021; Pezzulo et al. 2011).

There has been considerable success in developing lung organoids from various sub-anatomical parts of the lung using either adult-derived organoid or PSC techniques to create tracheospheres, bronchopsheres, bronchial organoids, alveoli organoids. The main drawback in using lung organoids is that an air-liquid-interface cannot be obtained as the organoids are embedded in a matrix and are inside out, with the lumen located on the inside of the organoid. There have been some recent examples where the lung organoids were dissociated and used in lung MPS devices to obtain the correct cell polarity (Hild 2016; Mulay et al. 2021; Rock et al. 2009; Sano et al. 2022; Tan et al. 2022)

Lung organoids have the ability to be grown and plated in sufficient numbers (e.g., 384-well format) to be used in medium-throughput screening assays. This is not the case at the moment with lung on a chip models. Among the most recent applications, airway organoids have been used to study viral

infections, e.g., by influenza virus (Hui et al. 2018), enterovirus (van der Sanden et al. 2018), RSV (Sachs et al. 2019) and SARS-COV-2 (Si et al. 2021).

Key cellular/biological components that should be considered when generating a lung MPS model include: 1) the use of either bronchioles or alveoli cells as part of the airway epithelium which creates an air interface, 2) matrix-embedded lung fibroblasts (Fibroblast layer that separates the epithelium from the endothelium), 3) the pulmonary endothelium (e.g., Lung microvascular endothelial cells) that could be either a vascular layer or vascularized network, and 4) the ability to incorporate immune cells (e.g., alveolar macrophages, T cells and neutrophils), particles, viruses or drug within the device (Ainslie et al. 2019; Benam et al. 2016; Nawroth et al. 2020). Such a device should allow the formation of a pseudo-stratified epithelium (thickness), correct cell polarity, mucus layer, beating ciliated cells, and goblet cells. There should be a layer of lung epithelial cells that directly interact with the air and form a tight barrier. Barrier integrity, cilia beat and mucus thickness should be assessed for the quality of the lung culture and compared to human lung slice cultures as a benchmarking model. There should be the capacity to allow the recruitment of immune cells to the epithelium through the vascular endothelium in response to a stimulus (e.g., either cytokine, injury or infection). There should be minimal adsorption of compounds to biopolymer (e.g., PDMS) or matrix components and the device should allow perfusion and mechanical forces to induce breathing. The device should have the ability to maintain a good physical barrier and cell architecture for more than 14 but ideally > 21 days (Ainslie et al. 2019; Benam et al. 2016; Nawroth et al. 2020).

Drug attrition due to pulmonary toxicity is a major cause in halting the development of inhaled therapeutics, so having a predictive lung model to better predict lung toxicity is important. However, to utilize a lung model it should be fit for purpose as there are a number of major mechanisms of toxicity that can be well-defined using reference compound sets of positive and negative controls. There are specific compounds that can be used to assess these mechanisms, which include BCNU or paraquat for cytotoxicity, 2) Ozone, NO_2, nitrofurantoin for reactive oxygen species, 3) trichloroethylene for metabolic activation, 4) IL-2, $CdCl_2$, LPS, paraquat for pulmonary edema, and 5) asbestos, silica for immune-mediated reactions.

When studies of drug metabolism are required in lung MPS models, the metabolic capacity for phase I metabolism (CYP enzymes) and phase II (GST, UGT, SULT, and NAT enzymes) needs to be proven. Characterizing the presence (or absence) of functional metabolism pathways within the lung MPS is

critical, considering that the potential for biological activation/deactivation of the inhaled drug could be investigated to the apical side through either liquid or aerosol. This has consequences on pharmacokinetic profile, along with possible changes in bioavailability, efficacy, and toxicity that could

be measured (Enlo-Scott et al. 2021). To perform drug absorption, distribution and elimination processes as well as drug-drug interaction in lung MPS, characterization of the expression, localization, and functionality of drug transporters covering both the ATP-binding cassette (ABC) superfamily and the solute-linked carrier (SLC) superfamily in the lung MPS should be conducted. The expression of drug transporters and metabolic enzymes should be equivalent to human lung tissue. The added benefit of lung MPS to static ALI or precision lung slice cultures is the clearance of the compound from the circulation due to the fluidic flow in the device on the vascular side of the device (Bosquillon 2010; Fowler et al. 2020). For efficacy/pharmacology testing there are opportunities to use lung MPS-based systems to evaluate drugs, disease mechanism or discover new targets for infectious diseases, and pulmonary disorders such as pulmonary fibrosis, asthma, IPF and pulmonary edema (Gard et al. 2021; Huh et al. 2010; Mejias et al. 2020; Nawroth et al. 2020; Schmidt et al. 2023; Si et al. 2021). Consideration should be taken on where the site of infection, disease or injury occur in the lung to better mimic the pathophysiology. There are two recent examples where different lung MPS were used to recapitulate SARS-COV-2 infection and test for novel compounds that would inhibit viral replication (Gard et al. 2021; Si et al. 2021).

There are opportunities to use lung MPS to mimic drug administration by delivering drug aerosol to the ALI epithelial layer of the MPS (Karra et al. 2022) or intravenously (IV) administering drug through the vascular side where bound or unbound drugs can be evaluated in both cases with the movement of fluid similar to the blood in the vascular side. Lung MPS devices that replicate and maintain an epithelial-vascular barrier are critical for the modeling of PK in a clinically meaningful way. Also having a fully vascularized endothelium channel enables the possibility to make human blood or PBMCs flowing through the device to facilitate pharmacokinetic/pharmacodynamic (PK/PD) modeling.

Aspects of lung MPS quality control

The following table S6 summarizes parameters to characterize different iPSC-derived mature lung cell types in a MPS.

Table S6. Recommendation of quality control for lung Microphysiological Systems (MPS).				
**The general features of the MPS**	**Key morphological, biochemical and functional parameters (or endpoints) characterize the model**	**Exemplar methods**	**Acceptance criteria**	**References**
**Cells**<br><br>**Upper airway**<br>**Lower airway**	Airway Epithelial cells<br>• Upper airway<br>• Lower airway<br>Endothelial cells<br>• Lung microvascular endothelial cells<br>Fibroblast cells<br>• Lung fibroblasts<br>Immune cells<br>• Alveolar macrophage, T cells, and neutrophils	Microscopy, Imaging, Transcriptomics	Presence of cell surface/intracellular markers for appropriate cell types	(Ainslie et al. 2019; Benam et al. 2016; Nawroth et al. 2020)
**Lung functionality**				
	• Air interface and Alveolar airway epithelium (appropriate ratio for segment)	Imaging and histopathology, Barrier integrity based on TEER or FITC-dextran, Imaging or H&E for Mucus layer, Live imaging for Cilia beating	Pseudo-stratified epithelium (thickness), mucus layer, beating ciliated cells, goblet cells. Layer of lung epithelial cells that directly interact with the air and forms a tight barrier.<br>Barrier integrity	(Ainslie et al. 2019; Benam et al. 2016; Nawroth et al. 2020)
	• Matrix-embedded fibroblasts	Imaging or histopathology	Fibroblast layer that separates the epithelium from the endothelium	(Ainslie et al. 2019; Benam et al. 2016; Nawroth et al. 2020)
	• Pulmonary endothelium	Imaging or histopathology	Either fully vascularized layer or vascularized network with low permeability	(Ainslie et al. 2019; Benam et al. 2016; Nawroth et al. 2020)
	• Media/blood (with leukocytes	Microscopy, Imaging	Ability to recruit immune cells to the epithelium through vascular endothelium	(Ainslie et al. 2019; Benam et al. 2016;

			in response to a stimuli	Nawroth et al. 2020)
	• Drug transporters	Imaging, qPCR or Western blot	Drug transporters should be equivalent to human lung tissue and have a similar cellular distribution and localization pattern	(Ainslie et al. 2019; Bosquillon 2010)
	• Metabolic enzymes (Phase I and II enzymes)	Imaging, qPCR or Western blot	Metabolic enzymes should be equivalent to human lung tissue and have a similar cellular distribution pattern	(Ainslie et al. 2019; Enlo-Scott et al. 2021)

References.

Ainslie GR, Davis M, Ewart L, et al. (2019) Microphysiological lung models to evaluate the safety of new pharmaceutical modalities: a biopharmaceutical perspective. Lab Chip 19(19):3152-3161 doi:10.1039/c9lc00492k

Benam KH, Villenave R, Lucchesi C, et al. (2016) Small airway-on-a-chip enables analysis of human lung inflammation and drug responses in vitro. Nat Methods 13(2):151-7 doi:10.1038/nmeth.3697

Bluhmki T, Bitzer S, Gindele JA, et al. (2020) Development of a miniaturized 96-Transwell air-liquid interface human small airway epithelial model. Sci Rep 10(1):13022 doi:10.1038/s41598-020-69948-2

Bosquillon C (2010) Drug transporters in the lung--do they play a role in the biopharmaceutics of inhaled drugs? J Pharm Sci 99(5):2240-55 doi:10.1002/jps.21995

Enlo-Scott Z, Backstrom E, Mudway I, Forbes B (2021) Drug metabolism in the lungs: opportunities for optimising inhaled medicines. Expert Opin Drug Metab Toxicol 17(5):611-625 doi:10.1080/17425255.2021.1908262

Fowler S, Chen WLK, Duignan DB, et al. (2020) Microphysiological systems for ADME-related applications: current status and recommendations for system development and characterization. Lab Chip 20(3):446-467 doi:10.1039/c9lc00857h

Gard AL, Luu RJ, Miller CR, et al. (2021) High-throughput human primary cell-based airway model for evaluating influenza, coronavirus, or other respiratory viruses in vitro. Sci Rep 11(1):14961 doi:10.1038/s41598-021-94095-7

Hild M, Jaffe, A.B., (2016) Production of 3-D Airway Organoids From Primary Human Airway Basal Cells and Their Use in High-Throughput Screening. Curr Protoc Stem Cell Biol IE.9.1-IE.9.15. doi:10.1002/cpsc.1.

Huh D, Matthews BD, Mammoto A, Montoya-Zavala M, Hsin HY, Ingber DE (2010) Reconstituting Organ-Level Lung Functions on a Chip. Science 328(5986):1662-1668 doi:10.1126/science.1188302

Hui KPY, Ching RHH, Chan SKH, et al. (2018) Tropism, replication competence, and innate immune responses of influenza virus: an analysis of human airway organoids and ex-vivo bronchus cultures. Lancet Respir Med 6(11):846-854 doi:10.1016/S2213-2600(18)30236-4

Karra N, Fernandes J, Swindle EJ, Morgan H (2022) Integrating an aerosolized drug delivery device with conventional static cultures and a dynamic airway barrier microphysiological system. Biomicrofluidics 16(5):054102 doi:10.1063/5.0100019

Maurer J, Walles T, Wiese-Rischke C (2023) Optimization of Primary Human Bronchial Epithelial 3D Cell Culture with Donor-Matched Fibroblasts and Comparison of Two Different Culture Media. Int J Mol Sci 24(4) doi:10.3390/ijms24044113
Mejias JC, Nelson MR, Liseth O, Roy K (2020) A 96-well format microvascularized human lung-on-a-chip platform for microphysiological modeling of fibrotic diseases. Lab Chip 20(19):3601-3611 doi:10.1039/d0lc00644k
Michi AN, Proud D (2021) A toolbox for studying respiratory viral infections using air-liquid interface cultures of human airway epithelial cells. Am J Physiol Lung Cell Mol Physiol 321(1):L263-L280 doi:10.1152/ajplung.00141.2021
Mulay A, Konda B, Garcia G, Jr., et al. (2021) SARS-CoV-2 infection of primary human lung epithelium for COVID-19 modeling and drug discovery. Cell Rep 35(5):109055 doi:10.1016/j.celrep.2021.109055
Nawroth JC, Lucchesi C, Cheng D, et al. (2020) A Microengineered Airway Lung Chip Models Key Features of Viral-induced Exacerbation of Asthma. Am J Respir Cell Mol Biol 63(5):591-600 doi:10.1165/rcmb.2020-0010MA
Pezzulo AA, Starner TD, Scheetz TE, et al. (2011) The air-liquid interface and use of primary cell cultures are important to recapitulate the transcriptional profile of in vivo airway epithelia. Am J Physiol Lung Cell Mol Physiol 300(1):L25-31 doi:10.1152/ajplung.00256.2010
Rock JR, Onaitis MW, Rawlins EL, et al. (2009) Basal cells as stem cells of the mouse trachea and human airway epithelium. Proc Natl Acad Sci U S A 106(31):12771-5 doi:10.1073/pnas.0906850106
Sachs N, Papaspyropoulos A, Zomer-van Ommen DD, et al. (2019) Long-term expanding human airway organoids for disease modeling. EMBO J 38(4) doi:10.15252/embj.2018100300
Sano E, Suzuki T, Hashimoto R, et al. (2022) Cell response analysis in SARS-CoV-2 infected bronchial organoids. Commun Biol 5(1):516 doi:10.1038/s42003-022-03499-2
Schmidt P, Narayan K, Li Y, et al. (2023) Antibody-mediated protection against symptomatic COVID-19 can be achieved at low serum neutralizing titers. Sci Transl Med 15(688):eadg2783 doi:10.1126/scitranslmed.adg2783
Si L, Bai H, Rodas M, et al. (2021) A human-airway-on-a-chip for the rapid identification of candidate antiviral therapeutics and prophylactics. Nat Biomed Eng 5(8):815-829 doi:10.1038/s41551-021-00718-9
Tan LS, Chen JT, Lim LY, Teo AKK (2022) Manufacturing clinical-grade human induced pluripotent stem cell-derived beta cells for diabetes treatment. Cell Prolif 55(8):e13232 doi:10.1111/cpr.13232
Travaglini KJ, Nabhan AN, Penland L, et al. (2020) A molecular cell atlas of the human lung from single-cell RNA sequencing. Nature 587(7835):619-625 doi:10.1038/s41586-020-2922-4
van der Sanden SMG, Sachs N, Koekkoek SM, et al. (2018) Enterovirus 71 infection of human airway organoids reveals VP1-145 as a viral infectivity determinant. Emerg Microbes Infect 7(1):84 doi:10.1038/s41426-018-0077-2

Supplementary material 6

Organ specific quality control: Brain

Aspects making brain MPS unique

The brain controls every process that regulates the body as well as cognition. It is structurally and functionally highly complex and is strongly susceptible to insults due to its low regeneration abilities after damage, with an even higher vulnerability of the developing brain[1].

The diagnoses of neurodevelopmental disabilities, including autism, attention-deficit hyperactivity disorder, and dyslexia, are increasing in frequency. On the other extremity of the lifespan, the aging of the population results in increasing onset of neuronal disorders and neurodegenerative diseases. In both cases, industrial chemicals are thought to play an important role (Cavaleri 2015; Grandjean and Landrigan 2014; Yan et al. 2016). Models are needed to study brain development and brain-related diseases, to assess the efficacy and safety of new compounds in early phases of drug development, and to evaluate the potential neurotoxicity of industrial chemicals, such as pesticides, solvents and nanomaterials, continuously released on the market. The brain is different to other organs in that it is protected by a blood-brain-barrier and immune-privileged with only microglia as resident immune cells.

Status of brain MPS development and examples of purposes of use

Numerous brain MPS showing different levels of organization have already been developed (for review, Chan et al. 2021). Cerebral organoids that mimic different brain regions within single organoids were produced by Lancaster et al. (2013). Alternatively, many protocols include the addition of specific cues that promote formation of a specific region of the brain, such as cortex (Birey et al. 2017; Magni et al. 2022; Paşca et al. 2015), hippocampus (Sakaguchi et al. 2015), cerebellum (Muguruma et al. 2010), thalamus (Xiang et al. 2019) and midbrain (Jo et al. 2016; Zagare et al. 2022). In a hybrid approach, multiple organoids each representing a different specific brain region are combined to generate “assembloids” (Chan et al. 2021; Xiang et al. 2019; Xiang et al. 2017), thereby achieving selective regional heterogeneity in a relatively controlled manner. For example, cortico-motor assembloids have been generated by assembling cerebral cortex/spinal cord organoids with human skeletal muscle (Andersen et al. 2020). This system demonstrated the potential of the self-assembly capacity of 3D cultures to form functional circuits. In this regard, mechanisms underlying the neuro-immune, neuro-endocrine, and gut-brain axis in humans can be investigated by utilizing specific organoid co-culture/assembling systems (Chukwurah et al. 2019). Eventually, these complex model

[1] https://www.cdc.gov/ncbddd/childdevelopment/early-brain-development.html#:~:text=Because%20children%27s%20brains%20are%20still,or%20toxins%2C%20such%20as%20lead.

systems representing different parts of the nervous system hold the potential to provide valuable insights into the mechanisms of normal and abnormal brain development and identify potential therapeutic approaches. Brain 3D cultures have already been abundantly used for disease modelling, including microcephaly, Zika virus infection, Alzheimer disease and other neurodegenerative disorders, as well as neurodevelopmental diseases, such as Timothy syndrome, Angelman syndrome and tuberous sclerosis (for review, Chen et al. 2022; Eichmüller and Knoblich 2022; Mayhew and Singhania 2022). Protocols to generate brain MPS containing various neuronal subtypes but without organization of brain structures, such as spheroids, have been successfully used for neurotoxicological investigations (Hogberg et al. 2013; Nunes et al. 2022; Pamies et al. 2018; Zhong et al. 2020) and for diseases modelling, including glioblastoma and demyelinating conditions (Abreu et al. 2018; Chesnut et al. 2021; Pamies et al. 2020). Most of these 3D cultures are composed of neurons, astrocytes and oligodendrocytes, but microglia have been reported to be innately present only by Ormel et al. (2018). However, microglial cells of various sources have been successfully added to spheroids (Abreu et al. 2018) and organoids (Sabate-Soler et al. 2022; Song et al. 2019).

Combining these leading-edge 3D organ-specific models with microfluidic technologies, referred to as brain OoCs, allows the modelling of the microenvironment by tightly controlling fluid flows. This ultimately improves culture conditions, by reducing shear stress, improving oxygen supply and by enhancing nutrient/waste exchanges (for review, Castiglione et al. 2022). For example, a multiplex automated microfluidic platform showed reduction in glycolytic and endoplasmic reticulum stress, compared to conventional organoid cultures (Seiler et al. 2022). However, another line of research is focusing on the development of vascularized brain organoids in order to avoid necrotic cores and to allow the culture of larger organoids. Various techniques have been reported (for review, LaMontagne et al. 2022), such as the addition of endothelial cells to cortical organoids, the fusion of vascular spheroids with cortical organoids or the co-differentiation of endothelial cells and neurons. Another avenue to facilitate vascularization of organoids uses microfluidic devices to generate vascular beds through an organoid containing matrix. Shear-stress is applied to two parallel endothelial cell (EC)-coated channels to induce EC migration into the central organoid-containing matrix until ECs from opposite channels meet to form a vessel (LaMontagne et al. 2022; Salmon et al. 2022). Another microfluidic method is to encapsulate organoids and a sacrificial scaffold in a hydrogel, remove the sacrificial scaffold, and perfuse media through empty space. These channels are then seeded with vascular cells (LaMontagne et al. 2022; Skylar-Scott et al. 2019). Although none of these methods fully capture the structure and function of an *in vivo* vascular unit, they are very promising in promoting organoid viability and function (Skylar-Scott et al. 2019). Microfluidic systems give a lot of flexibility in the choice of the experimental design. Several chambers, separated or connected, can be coupled on

the platform, allowing for example, the addition of blood-brain-barrier (Wevers et al. 2018). Furthermore, in even more complex designs, several devices can be coupled to enable the connection of distinct organs. For example, researchers have managed to establish a four-organ chip system from human iPSCs from a single donor (Ramme et al. 2019). The organ models were cultured in separate compartments of the bioreactor and connected by a microfluidic network, which mimics complex biological processes and can potentially allow for more accurate and clinically relevant testing of pharmacology and pharmacokinetics. These pave the way for achieving the full potential of stem cell derived models for developmental studies, disease modeling and drug screening research.

A promising new field of brain organoid research called Organoid Intelligence (OI) is emerging. OI aims to explore the potential of brain organoid to process complex input, study of the fundamental mechanisms of learning, and generate responses to control peripheral output devices (Morales Pantoja et al. 2023). Excitingly, Kagan et al. showed that they were able to train human iPSC-derived neurons to play games through electrophysiological stimulation and recording. This work demonstrated that neurons *in vitro* were able to learn in a systematic manner directed by input (Kagan et al. 2022). To achieve the potential of OI in neurological research, a combination or collaboration of multiple disciplines, including neurobiology, electrophysiology, machine learning and brain/machine interfaces as well as artificial intelligence, is essential. Although it is still in its early stages, we foresee that this emerging field will bring forth novel challenges and necessitate specific considerations in terms of QC.

Aspects of brain MPS quality control

The following table S7 summarizes parameters to characterize different iPSC-derived mature brain cell types in a MPS. Please take note that we have addressed numerous aspects of brain models in this table. However, as mentioned earlier, the QM plan must be tailored to the specific purpose, and some of the QC measures mentioned here may not be applicable to the model of interest.

Table S7. Recommendation of quality control for brain microphysiological systems (MPS).

**Characteristic**	**Key parameters (or endpoints) to characterize the model**	**Analytical methods**	**Acceptance criteria**	**References**
**Viability**		-Reduction of resazurin<br>- LDH activity	> 95 % of viable cells	
**Neurons**	Generic neuronal markers	qRT-PCR and immunostaining and/or western	Presence of positive cells equal to or above predefined thresholds	(Pamies et al. 2016)

		blots for Tubulin Beta 3 Class III (TUBB3), Neurofilaments (NFs), MAP2, NeuN	(depending on brain region of interest).<br>Number of positive cells/amount of protein increases with time in culture.<br>Presence of a network of fibers.	
	Neuronal subtypes (cholinergic, glutamatergic, GABAergic, dopaminergic, serotonergic)	Single-cell RNA-seq<br><br>qRT-PCR and/or immunostaining for cholinergic neurons, e.g.: Acetylcholinesterase (ACHE); glutamatergic neurons, e.g.: Vesicular Glutamate Transporter 1 and 2 (VGLUT1, VGLUT2); GABAergic neurons, e.g.: Glutamate Decarboxylase 1 (GAD1), $GABA_b$ receptor1/2; dopaminergic neurons, e.g.: Tyrosine Hydroxylase (TH), dopamine transporter; serotonergic neurons, e.g.: TH, serotonin transporter	Presence of positive cells equal to or above predefined thresholds.<br>Number of positive cells/amount of protein increases with time in culture.	(Pamies et al. 2016)
	Maturation state of neurons	qRT-PCR and/or Immunostaining: Immature neuronal markers, e.g.: Doublecortin, NeuroD1;<br>Mature neuronal markers, e.g.: MAP2, NeuN<br><br>GFP-labeled neurons to check somatodendritic organization	Presence of positive cells equal to or above predefined thresholds depending on the maturation/differentiation stage<br><br>Somatodendritic organization becomes more complex over time	

	Synapses	qRT-PCR and immunostaining and/or Western blots for Synaptophysin (SYP),Postsynaptic density protein 95 (PSD95) and Gephyrin.<br>Electron microscopy to check the morphology of the dendritic spines: mushroom, thin, stubby, filopodia	Presence of positive signals/proteins with overlapping pre- and post-synaptic puncta (in immunostaining).<br><br>Mature spines show mushroom morphology; Immature spines show thin/stubby/filopodia morphology	(Gentile et al. 2022; Pamies et al. 2016)
	Neuron function	Patch clamp recordings for ion channel function or intrinsic neuronal properties, such as resting membrane potential, membrane capacitance<br><br>Calcium imaging for spontaneous activities<br><br>Micro-electrode arrays (MEA) for neuronal network function	Presence of spontaneous electrical activity as well as synchronized (network) burst activity	(Pamies et al. 2016)
**Astrocytes**	Generic astrocytic markers	qRT-PCR and immunostaining and/or western blots for Glial Fibrillary Acidic Protein (GFAP), S100 Calcium Binding Protein B (S100B), Vimentin (VIM)	Presence of positive cells equal to or above predefined thresholds depending on the maturation/differentiation stage and the bran region of interest.<br>Number of positive cells / protein amount increases with time in culture.	(Pamies et al. 2016)
	Astrocyte function: Reactivity after exposure to LPS or a neurotoxin<br><br>Calcium signaling;	qRT-PCR and immunostaining and/or Western blots for GFAP, S100B, VIM	Reactivity: Generally increased number of intensively positive cells, but overall number could also decrease.	(Liddelow et al. 2017) (Hedegaard et al. 2020)

	spontaneous activity and evoked activity upon ATP, glutamate, potassium chloride.<br><br>Glutamate uptake;<br><br>Potassium channel function;<br><br>Form gap junction between cells	Specific markers for A1 and A2 astrocytes<br><br>Calcium imaging to check spontaneous and evoked activity<br><br>ELISA to check the level of glutamate in the culture medium over time<br><br>Patch clamp to check potassium ion function<br><br>Biocytin injection to the cell	Modification of cell bodies, shortening and enlargement of processes.<br><br>A1/2 phenotype: presence of the specific markers<br><br>Show spontaneous activity and also respond to ATP, glutamate, potassium chloride<br><br>Glutamate concentration in the culture medium reduce over time<br><br>Show potassium current<br><br>Biocytin may spread via gap junction in a population of cells	
**Oligodendrocytes**	General oligodendrocytic markers	qRT-PCR and immunostaining and/or Western for Oligodendrocyte Transcription Factor 1 and 2 (OLIG1, OLIG2), and immunostaining for O4	Presence of positive cells equal to or above predefined thresholds.<br><br>Number of positive cells / protein amount increases with time in culture	(Chesnut et al. 2021; Pamies et al. 2016)
	Myelin	Myelin Oligodendrocyte Glycoprotein (MOG), Myelin Basic Protein (MBP), Proteolipid Protein 1 (PLP1)<br><br>Electron microscopy (TEM)	Presence of positive cells and myelin sheath (visible in TEM).<br>Number of positive cells / protein amount increases with time in culture.	(Chesnut et al. 2021; Pamies et al. 2016)
**Microglia**	General microglial markers	qRT-PCR for Spi-1 Proto-oncogene (SPI1) also called Hematopoietic Transcription Factor PU.1 (PU.1), Colony Stimulating Factor 1 Receptor (CSF1R), Allograft Inflammatory Factor 1 (AIF1) also called	Development-dependent expression	(Ormel et al. 2018; Sabate-Soler et al. 2022)

		IBA-1, Integrin Subunit Alpha M (ITGAM), Triggering Receptor Expressed On Myeloid Cells 2 (TREM2), C-X3-C Motif Chemokine Receptor 1 (CX3CR1) and immunostaining for IBA-1 and PU.1		
	Microglia function: Reactivity after exposure to LPS or dexamethasone<br><br>Phagocytosis	Immunostaining for IBA-1<br><br>Phagocytosis assay	Shortening of cell processes<br><br>Increased phagocytosis	(Ormel et al. 2018; Sabate-Soler et al. 2022)
**Identification of brain regions**	Forebrain, hippocampus, cerebellum, thalamus, midbrain	Gene expression and protein level analysis of specific neuronal/glial markers depending on the brain region of interest.	For the characterization of the different brain regions, refer to the publications	(Birey et al. 2017; Jo et al. 2016; Magni et al. 2022; Muguruma et al. 2010; Paşca et al. 2015; Sakaguchi et al. 2015; Xiang et al. 2019; Zagare et al. 2022)

References.

Abreu CM, Gama L, Krasemann S, et al. (2018) Microglia Increase Inflammatory Responses in iPSC-Derived Human BrainSpheres. Front Microbiol 9:2766 doi:10.3389/fmicb.2018.02766

Andersen J, Revah O, Miura Y, et al. (2020) Generation of Functional Human 3D Cortico-Motor Assembloids. Cell 183(7):1913-1929 e26 doi:10.1016/j.cell.2020.11.017

Birey F, Andersen J, Makinson CD, et al. (2017) Assembly of functionally integrated human forebrain spheroids. Nature 545(7652):54-59 doi:10.1038/nature22330

Castiglione H, Vigneron PA, Baquerre C, Yates F, Rontard J, Honegger T (2022) Human Brain Organoids-on-Chip: Advances, Challenges, and Perspectives for Preclinical Applications. Pharmaceutics 14(11) doi:10.3390/pharmaceutics14112301

Cavaleri F (2015) Review of Amyotrophic Lateral Sclerosis, Parkinson's and Alzheimer's diseases helps further define pathology of the novel paradigm for Alzheimer's with heavy metals as primary disease cause. Med Hypotheses 85(6):779-90 doi:10.1016/j.mehy.2015.10.009

Chan WK, Fetit R, Griffiths R, Marshall H, Mason JO, Price DJ (2021) Using organoids to study human brain development and evolution. Dev Neurobiol 81(5):608-622 doi:10.1002/dneu.22819

Chen H, Jin X, Li T, Ye Z (2022) Brain organoids: Establishment and application. Front Cell Dev Biol 10:1029873 doi:10.3389/fcell.2022.1029873

Chesnut M, Paschoud H, Repond C, et al. (2021) Human IPSC-Derived Model to Study Myelin Disruption. Int J Mol Sci 22(17) doi:10.3390/ijms22179473

Chukwurah E, Osmundsen A, Davis SW, Lizarraga SB (2019) All Together Now: Modeling the Interaction of Neural With Non-neural Systems Using Organoid Models. Front Neurosci 13:582 doi:10.3389/fnins.2019.00582

Eichmüller OL, Knoblich JA (2022) Human cerebral organoids — a new tool for clinical neurology research. Nature Reviews Neurology 18(11):661-680 doi:10.1038/s41582-022-00723-9

Gentile JE, Carrizales MG, Koleske AJ (2022) Control of Synapse Structure and Function by Actin and Its Regulators. Cells 11(4) doi:10.3390/cells11040603

Grandjean P, Landrigan PJ (2014) Neurobehavioural effects of developmental toxicity. Lancet Neurol 13(3):330-8 doi:10.1016/S1474-4422(13)70278-3

Hedegaard A, Monzon-Sandoval J, Newey SE, Whiteley ES, Webber C, Akerman CJ (2020) Pro-maturational Effects of Human iPSC-Derived Cortical Astrocytes upon iPSC-Derived Cortical Neurons. Stem Cell Rep 15(1):38-51 doi:10.1016/j.stemcr.2020.05.003

Hogberg HT, Bressler J, Christian KM, et al. (2013) Toward a 3D model of human brain development for studying gene/environment interactions. Stem Cell Res Ther 4 Suppl 1(Suppl 1):S4 doi:10.1186/scrt365

Jo J, Xiao Y, Sun AX, et al. (2016) Midbrain-like Organoids from Human Pluripotent Stem Cells Contain Functional Dopaminergic and Neuromelanin-Producing Neurons. Cell Stem Cell 19(2):248-257 doi:10.1016/j.stem.2016.07.005

Kagan BJ, Kitchen AC, Tran NT, et al. (2022) In vitro neurons learn and exhibit sentience when embodied in a simulated game-world. Neuron 110(23):3952-3969 e8 doi:10.1016/j.neuron.2022.09.001

LaMontagne E, Muotri AR, Engler AJ (2022) Recent advancements and future requirements in vascularization of cortical organoids. Front Bioeng Biotechnol 10:1048731 doi:10.3389/fbioe.2022.1048731

Lancaster MA, Renner M, Martin CA, et al. (2013) Cerebral organoids model human brain development and microcephaly. Nature 501(7467):373-9 doi:10.1038/nature12517

Liddelow SA, Guttenplan KA, Clarke LE, et al. (2017) Neurotoxic reactive astrocytes are induced by activated microglia. Nature 541(7638):481-487 doi:10.1038/nature21029

Magni M, Bossi B, Conforti P, et al. (2022) Brain Regional Identity and Cell Type Specificity Landscape of Human Cortical Organoid Models. Int J Mol Sci 23(21) doi:10.3390/ijms232113159

Mayhew CN, Singhania R (2022) A review of protocols for brain organoids and applications for disease modeling. STAR Protoc 4(1):101860 doi:10.1016/j.xpro.2022.101860

Morales Pantoja IE, Smirnova L, Muotri AR, et al. (2023) First Organoid Intelligence (OI) workshop to form an OI community. Front Artif Intell 6:1116870 doi:10.3389/frai.2023.1116870

Muguruma K, Nishiyama A, Ono Y, et al. (2010) Ontogeny-recapitulating generation and tissue integration of ES cell-derived Purkinje cells. Nat Neurosci 13(10):1171-80 doi:10.1038/nn.2638

Nunes C, Singh P, Mazidi Z, et al. (2022) An in vitro strategy using multiple human induced pluripotent stem cell-derived models to assess the toxicity of chemicals: A case study on paraquat. Toxicol In Vitro 81:105333 doi:10.1016/j.tiv.2022.105333

Ormel PR, Vieira de Sa R, van Bodegraven EJ, et al. (2018) Microglia innately develop within cerebral organoids. Nat Commun 9(1):4167 doi:10.1038/s41467-018-06684-2

Pamies D, Barreras P, Block K, et al. (2016) A human brain microphysiological system derived from induced pluripotent stem cells to study neurological diseases and toxicity. ALTEX 34(3):362-376 doi:10.14573/altex.1609122

Pamies D, Block K, Lau P, et al. (2018) Rotenone exerts developmental neurotoxicity in a human brain spheroid model. Toxicol Appl Pharmacol doi:10.1016/j.taap.2018.02.003

Pamies D, Zurich MG, Hartung T (2020) Organotypic Models to Study Human Glioblastoma: Studying the Beast in Its Ecosystem. iScience 23(10):101633 doi:10.1016/j.isci.2020.101633
Paşca AM, Sloan SA, Clarke LE, et al. (2015) Functional cortical neurons and astrocytes from human pluripotent stem cells in 3D culture. Nature Methods 12(7):671-678 doi:10.1038/nmeth.3415
Ramme AP, Koenig L, Hasenberg T, et al. (2019) Autologous induced pluripotent stem cell-derived four-organ-chip. Future Sci OA 5(8):FSO413 doi:10.2144/fsoa-2019-0065
Sabate-Soler S, Nickels SL, Saraiva C, et al. (2022) Microglia integration into human midbrain organoids leads to increased neuronal maturation and functionality. Glia 70(7):1267-1288 doi:10.1002/glia.24167
Sakaguchi H, Kadoshima T, Soen M, et al. (2015) Generation of functional hippocampal neurons from self-organizing human embryonic stem cell-derived dorsomedial telencephalic tissue. Nat Commun 6:8896 doi:10.1038/ncomms9896
Salmon I, Grebenyuk S, Abdel Fattah AR, et al. (2022) Engineering neurovascular organoids with 3D printed microfluidic chips. Lab Chip 22(8):1615-1629 doi:10.1039/d1lc00535a
Seiler ST, Mantalas GL, Selberg J, et al. (2022) Modular automated microfluidic cell culture platform reduces glycolytic stress in cerebral cortex organoids. Sci Rep 12(1):20173 doi:10.1038/s41598-022-20096-9
Skylar-Scott MA, Uzel SGM, Nam LL, et al. (2019) Biomanufacturing of organ-specific tissues with high cellular density and embedded vascular channels. Sci Adv 5(9):eaaw2459 doi:10.1126/sciadv.aaw2459
Song L, Yuan X, Jones Z, et al. (2019) Functionalization of Brain Region-specific Spheroids with Isogenic Microglia-like Cells. Sci Rep 9(1):11055 doi:10.1038/s41598-019-47444-6
Wevers NR, Kasi DG, Gray T, et al. (2018) A perfused human blood-brain barrier on-a-chip for high-throughput assessment of barrier function and antibody transport. Fluids Barriers CNS 15(1):23 doi:10.1186/s12987-018-0108-3
Xiang Y, Tanaka Y, Cakir B, et al. (2019) hESC-Derived Thalamic Organoids Form Reciprocal Projections When Fused with Cortical Organoids. Cell Stem Cell 24(3):487-497.e7 doi:10.1016/j.stem.2018.12.015
Xiang Y, Tanaka Y, Patterson B, et al. (2017) Fusion of Regionally Specified hPSC-Derived Organoids Models Human Brain Development and Interneuron Migration. Cell Stem Cell 21(3):383-398 e7 doi:10.1016/j.stem.2017.07.007
Yan D, Zhang Y, Liu L, Yan H (2016) Pesticide exposure and risk of Alzheimer's disease: a systematic review and meta-analysis. Sci Rep 6:32222 doi:10.1038/srep32222
Zagare A, Barmpa K, Smajic S, et al. (2022) Midbrain organoids mimic early embryonic neurodevelopment and recapitulate LRRK2-p.Gly2019Ser-associated gene expression. The American Journal of Human Genetics 109(2):311-327 doi:https://doi.org/10.1016/j.ajhg.2021.12.009
Zhong X, Harris G, Smirnova L, et al. (2020) Antidepressant Paroxetine Exerts Developmental Neurotoxicity in an iPSC-Derived 3D Human Brain Model. Front Cell Neurosci 14:25 doi:10.3389/fncel.2020.00025

Supplementary material 7

Organ-specific quality control: Heart MPS

Aspects making heart MPS unique

The heart is one of our most vital organs and plays a crucial role in many physiological and pathophysiological processes. Its primary role is to pump blood through the circulatory system of your body at defined rhythm, speed and pressure. To achieve this, the human heart comprises four distinct chambers consisting of different types of highly specialized tissues. With respect to cardiac MPS, most systems focus on the myocardium, cardiac muscle tissue, which is sandwiched between endo- and epicardium the heart wall and whose contractive function is key for the pumping of blood. Besides the myocardium, heart valves are another component of the heart important for disease modeling and pharmacological testing. Although valvular heart diseases (VHDs) are a growing public health problem, only a few heart-valve-MPS have been introduced so far. One reason for this disbalance is that besides a plethora of diseases associated with the myocardium, failure of candidate drugs is most often associated with toxicity or dysfunction of myocardial tissue (Laverty et al. 2011). Hence, here, we will solely focus on MPSs of the cardiac muscle.

Status of heart MPS development and examples of purposes of use

To engineer heart tissue, initial approaches focused mainly on mimicking the anisotropic structure of the myocardium; anisotropy is a directional dependence, and in cardiac tissue, conduction velocity is anisotropic and its orientation is determined by myocyte direction. This has been attempted in bioengineering, for instance, via surface patterning of fibronectin lines forcing monolayers of cardiomyocytes (CMs) to self-align (Bursac et al. 2002; Grosberg et al. 2011; Wang et al. 2014) or by embedding in engineered ECMs (e.g., collagen, fibrin, Matrigel) as more physiological 3D tissues (Liau et al. 2011; Thavandiran et al. 2013; Zhao et al. 2019). Integrating these microtissues in tailored microfluidic platforms, heart-on-chip systems, added a further layer of complexity by providing vasculature-like perfusion and mimicking in vivo-transport processes (Marsano et al. 2016; Mathur et al. 2015; Schneider et al. 2022). Other approaches to generate myocardial MPS focus more on scalability and compatibility with higher-throughput experimentation while cutting back on mimicking native structural organization or certain functional aspects: cardiac spheroids or aggregates (Giacomelli et al. 2020) generated by forced aggregation of cell suspensions and cardiac organoids based on self-assembly of differentiating stem cells (Drakhlis et al. 2021; Ma et al. 2015; Richards et al. 2020; Silva et al. 2021).

In terms of cell sources, over the last decade, the field of myocardial MPS shifted almost entirely to predominately human iPSC-derived tissues (Giacomelli et al. 2020; Mathur et al. 2015; Nunes et al.

2013; Ronaldson-Bouchard et al. 2018; Schneider et al. 2022; Thavandiran et al. 2013; Wang et al. 2014), in contrast to earlier work using mostly neonatal rodent CMs (Bursac et al. 2002; Grosberg et al. 2011; Hansen et al. 2010; Liau et al. 2011; Marsano et al. 2016). Advances in iPSC differentiation protocols further enable the generation of models based on atrial or ventricular CMs to account for chamber-specific differences in electrophysiology and drug response (Zhao et al. 2019). Along a similar line, microtissues were initially often formed from cell mixtures directly resulting from the respective differentiation. Since this rather undefined mixture of CMs and further (stromal) cell types can vary strongly depending on protocol, iPSC line and individual differentiation processes and purification approaches to achieve pure CM populations have been increasingly employed. To properly form robust and connected tissues, however, stromal cell types, particularly fibroblasts, are required. Hence, purified CMs are typically mixed with, e.g., (cardiac) fibroblasts, endothelial cells or immune cells, resulting not only in more standardized microtissues but also paving the way for studies dissecting the respective contributions of the individual cell types to tissue function (Giacomelli et al. 2020). The switch to stem cell-derived cardiac tissue did not come without challenges, with the immature phenotype of derived CMs being one of the major limitations. While various approaches based, for instance, on electrical stimulation (Ronaldson-Bouchard et al. 2018), inclusion of supporting cell types (Giacomelli et al. 2020), or metabolic maturation (Huebsch et al. 2022) showed promising results, insufficient CM maturity remains a significant challenge.

Crucial for studies on maturation but also for the generation of controlled baseline conditions is the implementation of actuators enabling either electrical or mechanical stimulation of the engineered cardiac tissues (Stoppel et al. 2016). To allow for the most commonly employed electrical stimulation, a variety of approaches has been established ranging from direct patterning of electrodes, integration of conducting wires (Zhao et al. 2019), or pacing via media channels (Schneider et al. 2022). Fewer models implemented solutions for the active mechanical stimulation of the cardiac tissue constructs, e.g., via the upward bending of flexible membranes underneath the tissues (Marsano et al. 2016; Parsa et al. 2017).

In terms of tissue characterization, a wide range of both structural and functional readouts have been implemented for cardiac MPS. Key structural characteristics are, for instance, the anisotropic orientation on multiple levels (tissue, cell and nuclei), cell size and overall morphology as well as the tissue ultrastructure – aspects that can be characterized via immunofluorescence microscopy, immunohistochemistry or electron microscopy. Key functional characteristics are gene expression, biomechanical activity (e.g., contractile force generation and dynamics), electrophysiological activity (e.g., excitability, conduction velocity, action potential, Ca^{2+}-handling), as well as the electro-mechanical coupling. Less studied and considered are cardiac energy metabolism and secretion

patterns. To characterize biomechanical activity, cardiac MPS often stretch the engineered cardiac microtissues around flexible pillars or wires (Godier-Furnemont et al. 2015; Hansen et al. 2010; Zhao et al. 2019). Further approaches are the patterning of monolayers on “cantilever-like” structures (Wang et al. 2014) or the use of less quantitative video-microscopy-based motion tracking analysis (Schneider et al. 2022). To monitor electrophysiological activity, tissues are still often dissociated to conduct traditional patch-clamp or microelectrode-based electrophysiology while more advanced platforms directly integrate micro-electrodes (Vivas et al. 2022). Other studies rely on high-speed fluorescence video microscopy combined with voltage sensitive (Huebsch et al. 2022) or calcium indicator dyes (Ronaldson-Bouchard et al. 2018) or alternatively reporter cell lines (Huebsch et al. 2022).

Several of both structural and functional characteristics differ considerably between neonatal and mature cardiomyocytes (e.g., conduction velocity, action potential duration, sarcomere length, t-tubule formation, Ca^{2+}-handling, force-frequency relation) (Karbassi et al. 2020). Hence, they are frequently drawn on as hallmarks of maturation.

With respect to applications, most cardiac MPS are still utilized primarily for mechanistic research (e.g., biomedical, investigative toxicology, pharmacology). Driven by commercialization of models as well as public-private partnerships with pharmaceutical industry, however, cardiac MPS are also increasingly gaining traction for translational safety research. Besides generic healthy models, more and more studies also focus on disease models of genetic (Ma et al. 2015) or non-genetic (Wang et al. 2021; Yadid et al. 2020) cardiomyopathies as well as on patient-specific models for personalized or precision medicine applications.

Aspects of heart MPS quality control

The following table S8 summarizes parameters to characterize different iPSC-derived mature heart cell types in a MPS.

Table S8. Recommendation of quality control for heart microphysiological systems (MPS).

**The general features of the MPS**	**Key morphological, biochemical, and functional parameters (or endpoints) to characterize the model**	**Exemplar analytical methods**	**Acceptance criteria**	**References**

**Cardiomyocyte properties**	Cell size	IF microscopy	Physiological hypertrophy	(Karbassi et al. 2020; Vinken and Hengstler 2018)
	Cell morphology	IF microscopy	Cellular anisotropy Rod-shaped nuclei	(Karbassi et al. 2020; Vinken and Hengstler 2018)
	Mitochondria content	IF microscopy	Increased number and fatty acid oxidation	(Karbassi et al. 2020; Vinken and Hengstler 2018)
	Ion channel expression	Gene expression	Positive expression above a certain predefined threshold	(Karbassi et al. 2020; Vinken and Hengstler 2018)
	Ventricular vs atrial identity	Gene expression	Positive expression above a certain predefined threshold	(Vinken and Hengstler 2018)
**Non-CM population**	Presence and identity of (cardiac) fibroblasts	IF microscopy, Flow cytometry	(Patho-) physiological ratios	(Souders et al. 2009)
	Presence and identity of endothelial cells	IF microscopy, Flow cytometry IF microscopy, Flow cytometry	(Patho-) physiological ratios	
	Presence and identity of further cell types	IF microscopy, Flow cytometry IF microscopy, Flow cytometry	(Patho-) physiological ratios	
**Tissue structure**	alignment	IF microscopy	Anisotropic 3D structure	(Karbassi et al. 2020; Vinken and Hengstler 2018)
	cell density	IF microscopy	Physiological cell densities around $10^8/cm^3$	(Chiu and Radisic 2013)
	extracellular matrix	IF microscopy, Second-harmonic imaging microscopy, Raman microspectroscopy	Physiological presence of structural and non-structural proteins including strands of	(Rienks et al. 2014)

			collagen and elastin	
	ultrastructure	Electron microscopy	Aligned myofibrils Registration of sarcomeres	(Karbassi et al. 2020)
	Intercalated disc formation	Electron microscopy	Polarized gap and adhesive junctions	(Karbassi et al. 2020)
**Electrophysiology**	Excitability	Microscopy		
	Action/Field potential	Micro-electrode arrays	Increased action potential duration and amplitude	(Karbassi et al. 2020)
	Conduction velocity	Micro-electrode arrays, Ca^{2+} imaging	CVs in the range of 0.5 m/s	(Clayton et al. 2011)
**Mechanical activity**	Beat rate	Force sensors / optical motion tracking	Controllable by electrical pacing	
	Contraction	Force sensors	Anisotropic contraction w/ forces in the physiological range (approx. 20 mN/mm^2)	(Tzatzalos et al. 2016)
	Stimulable contraction forces	Force sensors	Response to inotropic agents	
	Force frequency relationship	Force sensors	Positive relationship as hallmark of maturation	(Endoh 2004)

References.

Bursac N, Parker KK, Iravanian S, Tung L (2002) Cardiomyocyte cultures with controlled macroscopic anisotropy: a model for functional electrophysiological studies of cardiac muscle. Circ Res 91(12):e45-54 doi:10.1161/01.res.0000047530.88338.eb

Chiu LLY, Radisic M (2013) Cardiac tissue engineering. Current Opinion in Chemical Engineering 2(1):41-52 doi:https://doi.org/10.1016/j.coche.2013.01.002

Clayton RH, Bernus O, Cherry EM, et al. (2011) Models of cardiac tissue electrophysiology: progress, challenges and open questions. Prog Biophys Mol Biol 104(1-3):22-48 doi:10.1016/j.pbiomolbio.2010.05.008

Drakhlis L, Biswanath S, Farr CM, et al. (2021) Human heart-forming organoids recapitulate early heart and foregut development. Nat Biotechnol 39(6):737-746 doi:10.1038/s41587-021-00815-9

Endoh M (2004) Force-frequency relationship in intact mammalian ventricular myocardium: physiological and pathophysiological relevance. Eur J Pharmacol 500(1-3):73-86 doi:10.1016/j.ejphar.2004.07.013

Giacomelli E, Meraviglia V, Campostrini G, et al. (2020) Human-iPSC-Derived Cardiac Stromal Cells Enhance Maturation in 3D Cardiac Microtissues and Reveal Non-cardiomyocyte Contributions to Heart Disease. Cell Stem Cell 26(6):862-879 e11 doi:10.1016/j.stem.2020.05.004

Godier-Furnemont AF, Tiburcy M, Wagner E, et al. (2015) Physiologic force-frequency response in engineered heart muscle by electromechanical stimulation. Biomaterials 60:82-91 doi:10.1016/j.biomaterials.2015.03.055

Grosberg A, Alford PW, McCain ML, Parker KK (2011) Ensembles of engineered cardiac tissues for physiological and pharmacological study: heart on a chip. Lab Chip 11(24):4165-73 doi:10.1039/c1lc20557a

Hansen A, Eder A, Bonstrup M, et al. (2010) Development of a drug screening platform based on engineered heart tissue. Circ Res 107(1):35-44 doi:10.1161/CIRCRESAHA.109.211458

Huebsch N, Charrez B, Neiman G, et al. (2022) Metabolically driven maturation of human-induced-pluripotent-stem-cell-derived cardiac microtissues on microfluidic chips. Nat Biomed Eng 6(4):372-388 doi:10.1038/s41551-022-00884-4

Karbassi E, Fenix A, Marchiano S, et al. (2020) Cardiomyocyte maturation: advances in knowledge and implications for regenerative medicine. Nat Rev Cardiol 17(6):341-359 doi:10.1038/s41569-019-0331-x

Laverty H, Benson C, Cartwright E, et al. (2011) How can we improve our understanding of cardiovascular safety liabilities to develop safer medicines? Br J Pharmacol 163(4):675-93 doi:10.1111/j.1476-5381.2011.01255.x

Liau B, Christoforou N, Leong KW, Bursac N (2011) Pluripotent stem cell-derived cardiac tissue patch with advanced structure and function. Biomaterials 32(35):9180-7 doi:10.1016/j.biomaterials.2011.08.050

Ma Z, Wang J, Loskill P, et al. (2015) Self-organizing human cardiac microchambers mediated by geometric confinement. Nat Commun 6:7413 doi:10.1038/ncomms8413

Marsano A, Conficconi C, Lemme M, et al. (2016) Beating heart on a chip: a novel microfluidic platform to generate functional 3D cardiac microtissues. Lab Chip 16(3):599-610 doi:10.1039/c5lc01356a

Mathur A, Loskill P, Shao K, et al. (2015) Human iPSC-based cardiac microphysiological system for drug screening applications. Sci Rep 5:8883 doi:10.1038/srep08883

Nunes SS, Miklas JW, Liu J, et al. (2013) Biowire: a platform for maturation of human pluripotent stem cell-derived cardiomyocytes. Nat Methods 10(8):781-7 doi:10.1038/nmeth.2524

Parsa H, Wang BZ, Vunjak-Novakovic G (2017) A microfluidic platform for the high-throughput study of pathological cardiac hypertrophy. Lab Chip 17(19):3264-3271 doi:10.1039/c7lc00415j

Richards DJ, Li Y, Kerr CM, et al. (2020) Human cardiac organoids for the modelling of myocardial infarction and drug cardiotoxicity. Nat Biomed Eng 4(4):446-462 doi:10.1038/s41551-020-0539-4

Rienks M, Papageorgiou AP, Frangogiannis NG, Heymans S (2014) Myocardial extracellular matrix: an ever-changing and diverse entity. Circ Res 114(5):872-88 doi:10.1161/CIRCRESAHA.114.302533

Ronaldson-Bouchard K, Ma SP, Yeager K, et al. (2018) Advanced maturation of human cardiac tissue grown from pluripotent stem cells. Nature 556(7700):239-243 doi:10.1038/s41586-018-0016-3

Schneider O, Moruzzi A, Fuchs S, et al. (2022) Fusing spheroids to aligned mu-tissues in a heart-on-chip featuring oxygen sensing and electrical pacing capabilities. Mater Today Bio 15:100280 doi:10.1016/j.mtbio.2022.100280

Silva AC, Matthys OB, Joy DA, et al. (2021) Co-emergence of cardiac and gut tissues promotes cardiomyocyte maturation within human iPSC-derived organoids. Cell Stem Cell 28(12):2137-2152 e6 doi:10.1016/j.stem.2021.11.007

Souders CA, Bowers SL, Baudino TA (2009) Cardiac fibroblast: the renaissance cell. Circ Res 105(12):1164-76 doi:10.1161/CIRCRESAHA.109.209809

Stoppel WL, Kaplan DL, Black LD, 3rd (2016) Electrical and mechanical stimulation of cardiac cells and tissue constructs. Adv Drug Deliv Rev 96:135-55 doi:10.1016/j.addr.2015.07.009

Thavandiran N, Dubois N, Mikryukov A, et al. (2013) Design and formulation of functional pluripotent stem cell-derived cardiac microtissues. Proc Natl Acad Sci U S A 110(49):E4698-707 doi:10.1073/pnas.1311120110

Tzatzalos E, Abilez OJ, Shukla P, Wu JC (2016) Engineered heart tissues and induced pluripotent stem cells: Macro- and microstructures for disease modeling, drug screening, and translational studies. Adv Drug Deliv Rev 96:234-244 doi:10.1016/j.addr.2015.09.010

Vinken M, Hengstler JG (2018) Characterization of hepatocyte-based in vitro systems for reliable toxicity testing. Arch Toxicol 92(10):2981-2986 doi:10.1007/s00204-018-2297-6

Vivas A, IJspeert C, Pan JY, et al. (2022) Generation and Culture of Cardiac Microtissues in a Microfluidic Chip with a Reversible Open Top Enables Electrical Pacing, Dynamic Drug Dosing and Endothelial Cell Co-Culture. Adv Mater Technol-Us 7(7) doi:ARTN 2101355

10.1002/admt.202101355

Wang EY, Kuzmanov U, Smith JB, et al. (2021) An organ-on-a-chip model for pre-clinical drug evaluation in progressive non-genetic cardiomyopathy. J Mol Cell Cardiol 160:97-110 doi:10.1016/j.yjmcc.2021.06.012

Wang G, McCain ML, Yang L, et al. (2014) Modeling the mitochondrial cardiomyopathy of Barth syndrome with induced pluripotent stem cell and heart-on-chip technologies. Nat Med 20(6):616-23 doi:10.1038/nm.3545

Yadid M, Lind JU, Ardona HAM, et al. (2020) Endothelial extracellular vesicles contain protective proteins and rescue ischemia-reperfusion injury in a human heart-on-chip. Sci Transl Med 12(565) doi:10.1126/scitranslmed.aax8005

Zhao Y, Rafatian N, Feric NT, et al. (2019) A Platform for Generation of Chamber-Specific Cardiac Tissues and Disease Modeling. Cell 176(4):913-927 e18 doi:10.1016/j.cell.2018.11.042

Supplementary material 8

Organ specific quality control: Pancreatic MPS

Aspects making pancreas MPS unique

Generation of iPSC-derived pancreatic islets or beta cells that can function similarly as *bona fide* human islets is of significant interest due to the huge prevalence of diabetes conditions (Lau et al. 2021; Tan et al. 2022). The ability to control blood glucose levels in a physiological manner via the appropriate and timely secretion of insulin is now well-known to avert devastating healthcare consequences due to diabetic complications (NIDDK 2022).

Status of pancreas MPS development and examples of purposes of use

In the past decade, significant progress has been made in generating functional insulin$^+$ pancreatic beta-like cells from human PSCs (Pagliuca et al. 2014; Rezania et al. 2014; Veres et al. 2019). In fact, recent clinical trials suggest that human PSC-derived pancreatic cells are now inching closer toward clinical utility (Ramzy et al. 2021; Shapiro et al. 2021). Human PSC-derived pancreatic islet or beta cell organoids have also been placed on microfluidic chips to simulate physiological environment at micro scale, for the purpose of studying organ cross-talk (Tao et al. 2022), disease modelling, and even drug screening (Yin et al. 2022). The use of islet/beta cell organoids on a chip has a significant advantage of requiring only small quantities of cells and yet at the same time allow for multiple biochemical analyses. Here, we only focus on human PSC-derived islet/beta cell organoids and not those from other cell sources. Kim et al. generated a nanotopographical system to improve differentiation of PSCs into pancreatic organoids, suggesting that gradient nanopatterned chips can generate optimally-sized beta cell organoids for cell therapy (Kim et al. 2019). Liu et al. developed a droplet microfluidic system to encapsulate iPSC-derived pancreatic endocrine cells into hybrid hydrogel capsules, generating islet-like organoids that self-organized (Liu et al. 2020). Essaouiba et al. compared 2D monolayers vs 3D organoids and reported that 3D organoids in microfluidic environment were able to express high levels of beta cell markers, exhibit glucose-responsiveness and can be successfully maintained in perfusion (Essaouiba et al. 2021). Tao et al. developed a multilayer microfluidic system to differentiate iPSCs into islet organoids under perfused conditions, allowing real-time imaging and monitoring of islet organoid growth (Tao et al. 2022; Tao et al. 2019). Tao et al. then developed a microfluidic multi-organoid system to study pancreatic islet-liver cross-talk, both under physiological and high-glucose conditions (Tao et al. 2022). Overall, a small number of human iPSC-derived islet/beta cell organoids-on-chip model have been developed. Future efforts would require a demonstration of new biological insights derived from these OoC models to uncover new aspects of beta cell biology both in physiological and

pathophysiological conditions. It also remains to be seen whether these platforms will result in new diabetes drug discovery.

Aspects of pancreas MPS quality control

In determining the characteristics required to qualify mature human beta cells, it would be a combination of beta cell identity markers as well as actual functional attributes (Barsby and Otonkoski 2022; Kaestner et al. 2021). For beta cell identity (Table S9), the cells should express beta cell-enriched transcription factors such as PDX1, NKX6.1, MAFA, proteins involved in beta cell hormonal function such as INS, KATP and calcium channels, hormone processing protein PC1/3, and vesicle trafficking/exocytosis genes such as STXBP1 and STX1A (Cataldo et al. 2022). Ideally, there should be a high percentage of mono-hormonal INS^+ beta cells. For functionality, the iPSC-derived beta-like cells should respond rapidly to glucose concentrations and secrete appropriate and sufficient amounts of insulin that can bring glucose levels back to physiological levels. The following table S9 summarizes parameters to characterize different iPSC-derived mature islet cell types in a MPS.

Table S9. Recommendation of quality control for Islet microphysiological systems (MPS).

**The general features of the MPS**	**Key morphological, biochemical, and functional parameters (for endpoints) to characterize the model**	**Exemplar analytical methods**	**Acceptance criteria**	**References**
**Pancreatic beta cell identity**	Expression of insulin hormone (INS)	Fluorescence imaging, Flow cytometry analyses	Presence of > 40 % INS^+ cells	(Barsby and Otonkoski 2022; Kaestner et al. 2021)
	Expression of transcription factors	Fluorescence imaging, Flow cytometry analyses	Beta cell-enriched transcription factors (PDX1, NKX6.1, MAFA). > 60 % positivity	(Barsby and Otonkoski 2022; Kaestner et al. 2021)

	Expression of potassium and calcium channels involved in beta cell insulin secretion function	Fluorescence imaging	Proteins involved in beta cell hormonal function (INS, KCNJ11, ABCC8, $Ca_v1.3$, $Ca_v2.1$, $Ca_v3.2$)	(Yang and Berggren 2006)
	Expression of insulin hormone processing enzymes	Fluorescence imaging	Proteins involved in INS hormone processing (PC1/3)	(Meier et al. 2022)
	Expression of insulin vesicle trafficking and exocytosis proteins	Fluorescence imaging	Proteins responsible for insulin vesicle trafficking and exocytosis (STX1, SNAP25, VAMP2, synaptotagmin, Munc18)	(Hou et al. 2009) Cataldo et al., 2022
**Pancreatic beta cell function**	Senses high glucose and secretes insulin	Human insulin ELISA, human C-peptide ELISA	Ability to sense glucose and release appropriate amounts of insulin in response to high glucose in a timely manner	(Henquin 2011)
	Secretes insulin in a biphasic manner	Human insulin ELISA, human C-peptide ELISA	Exhibits first phase and second phase insulin secretion	(Marchetti et al. 2017)

	Secretes insulin in response to non-glucose secretagogues	Human insulin ELISA, human C-peptide ELISA	Responsive to non-glucose secretagogues, including amino acids (e.g. leucine, glutamate, arginine) and free fatty acids	(Marchetti et al. 2017)

References.

Barsby T, Otonkoski T (2022) Maturation of beta cells: lessons from in vivo and in vitro models. Diabetologia 65(6):917-930 doi:10.1007/s00125-022-05672-y

Cataldo LR, Singh T, Achanta K, et al. (2022) MAFA and MAFB regulate exocytosis-related genes in human beta-cells. Acta Physiol (Oxf) 234(2):e13761 doi:10.1111/apha.13761

Essaouiba A, Jellali R, Shinohara M, et al. (2021) Analysis of the behavior of 2D monolayers and 3D spheroid human pancreatic beta cells derived from induced pluripotent stem cells in a microfluidic environment. J Biotechnol 330:45-56 doi:10.1016/j.jbiotec.2021.02.009

Henquin JC (2011) The dual control of insulin secretion by glucose involves triggering and amplifying pathways in beta-cells. Diabetes Res Clin Pract 93 Suppl 1:S27-31 doi:10.1016/S0168-8227(11)70010-9

Hou JC, Min L, Pessin JE (2009) Insulin granule biogenesis, trafficking and exocytosis. Vitam Horm 80:473-506 doi:10.1016/S0083-6729(08)00616-X

Kaestner KH, Campbell-Thompson M, Dor Y, et al. (2021) What is a beta cell? - Chapter I in the Human Islet Research Network (HIRN) review series. Mol Metab 53:101323 doi:10.1016/j.molmet.2021.101323

Kim JH, Park BG, Kim SK, et al. (2019) Nanotopographical regulation of pancreatic islet-like cluster formation from human pluripotent stem cells using a gradient-pattern chip. Acta Biomater 95:337-347 doi:10.1016/j.actbio.2018.12.011

Lau HH, Gan SU, Lickert H, Shapiro AMJ, Lee KO, Teo AKK (2021) Charting the next century of insulin replacement with cell and gene therapies. Med (N Y) 2(10):1138-1162 doi:10.1016/j.medj.2021.09.001

Liu H, Wang Y, Wang H, et al. (2020) A Droplet Microfluidic System to Fabricate Hybrid Capsules Enabling Stem Cell Organoid Engineering. Adv Sci (Weinh) 7(11):1903739 doi:10.1002/advs.201903739

Marchetti P, Bugliani M, De Tata V, Suleiman M, Marselli L (2017) Pancreatic Beta Cell Identity in Humans and the Role of Type 2 Diabetes. Front Cell Dev Biol 5 doi:ARTN 55

10.3389/fcell.2017.00055

Meier DT, Rachid L, Wiedemann SJ, et al. (2022) Prohormone convertase 1/3 deficiency causes obesity due to impaired proinsulin processing. Nat Commun 13(1):4761 doi:10.1038/s41467-022-32509-4

NIDDK (2022) Blood Glucose Control Studies for Type 1 Diabetes: DCCT & EDIC. NIDDK report

Pagliuca FW, Millman JR, Gurtler M, et al. (2014) Generation of functional human pancreatic beta cells in vitro. Cell 159(2):428-39 doi:10.1016/j.cell.2014.09.040
Ramzy A, Thompson DM, Ward-Hartstonge KA, et al. (2021) Implanted pluripotent stem-cell-derived pancreatic endoderm cells secrete glucose-responsive C-peptide in patients with type 1 diabetes. Cell Stem Cell 28(12):2047-2061 e5 doi:10.1016/j.stem.2021.10.003
Rezania A, Bruin JE, Arora P, et al. (2014) Reversal of diabetes with insulin-producing cells derived in vitro from human pluripotent stem cells. Nat Biotechnol 32(11):1121-33 doi:10.1038/nbt.3033
Shapiro AMJ, Thompson D, Donner TW, et al. (2021) Insulin expression and C-peptide in type 1 diabetes subjects implanted with stem cell-derived pancreatic endoderm cells in an encapsulation device. Cell Rep Med 2(12) doi:ARTN 100466

10.1016/j.xcrm.2021.100466
Tan LS, Chen JT, Lim LY, Teo AKK (2022) Manufacturing clinical-grade human induced pluripotent stem cell-derived beta cells for diabetes treatment. Cell Prolif 55(8):e13232 doi:10.1111/cpr.13232
Tao TT, Deng PW, Wang YQ, et al. (2022) Microengineered Multi-Organoid System from hiPSCs to Recapitulate Human Liver-Islet Axis in Normal and Type 2 Diabetes. Adv Sci 9(5) doi:ARTN 2103495

10.1002/advs.202103495
Tao TT, Wang YQ, Chen WW, et al. (2019) Engineering human islet organoids from iPSCs using an organ-on-chip platform. Lab Chip 19(6):948-958 doi:10.1039/c8lc01298a
Veres A, Faust AL, Bushnell HL, et al. (2019) Charting cellular identity during human in vitro beta-cell differentiation. Nature 569(7756):368-373 doi:10.1038/s41586-019-1168-5
Yang SN, Berggren PO (2006) The role of voltage-gated calcium channels in pancreatic beta-cell physiology and pathophysiology. Endocr Rev 27(6):621-76 doi:10.1210/er.2005-0888
Yin J, Meng H, Lin J, Ji W, Xu T, Liu H (2022) Pancreatic islet organoids-on-a-chip: how far have we gone? J Nanobiotechnology 20(1):308 doi:10.1186/s12951-022-01518-2

Supplementary material 9

Other organ specific quality control

In this manuscript, we have addressed some of the most crucial organ-specific microphysiological systems. Regrettably, due to the document's length constraints, we cannot comprehensively cover all the existing or in-development microphysiological systems. Nevertheless, we acknowledge that many other organs are equally significant as those discussed here. Therefore, we do not want to miss the chance to briefly touch upon key considerations in some additional MPS, such as the skin, retina, thyroid, ovaries, fetal structures, bone marrow, and lymph nodes.

Skin MPS

Similar to other organs, the skin has a collection of cells that are arranged accordingly to the structural layer each cell belong to. Commonly known skin cells include keratinocytes (epidermis), fibroblasts (both layers of the dermis), reticular fibroblasts (lower dermis) and preadipocytes/adipocytes (hypodermis). But many other cells (i.e., skin cells and others), make up the complex layering of the skin and thus enable its functionality. Some of those include melanocytes (pigmentation), Langerhans cells (immune function), Merkel cells (sensation), hair follicle (hair growth) and sebaceous glands (oil storage and release). Although there are many more cells that make up the skin, the major component of the skin is the extracellular matrix comprise primarily of collagen. The complexity of the skin structure warrants the use of systems such as biopsis and other in vivo-like sources of skin. But, due to limitations in accessibility, reproducibility, and ethical approval procedures, biopsis are not the best way to study this model. Thus, an MPS that can provide physiologically relevant physical structure, biochemical and mechanical cues while at the same perfusion of the cells to remove any accumulated waste, will greatly facilitate the study of this organ. Siriam et al. and Mori et al. provided examples of MPS for skin-on-a-chip (SoC) systems with results that showed improved skin cell growth with integrated vascular connections (Mori et al. 2017; Sriram et al. 2018). Others have shown how mechanical cues can improve the epidermal layer thickness in vitro and how the orientation in the skin “construct” can change depending on how the applied mechanical cue is directed (Tokuyama et al. 2015; Tremblay et al. 2014). All in all, the features of this type of systems, alongside with the appropriate key parameters and methods to measure those parameters should be part of the recommendations for SoC quality control. For example, parameters that should be considered, from the ones commonly used in the skin model are tight junction disruption (Ramadan and Ting 2016; Srinivasan et al. 2015) and biochemical markers of metabolic activity (e.g., pH, oxygen, glucose, and lactate) (Kieninger et al. 2018). Methods to measure those specific parameters include, for example

for tight junction disruptions, trans epithelial/endothelial electrical resistance (TEER) is largely used. Whereas to measure biochemical markers a combination of optical, physical, and biochemical sensors is used (Zhang et al. 2017).

Retina MPS

Retinogenesis involves creating the seven types of retinal cells. During vertebrate development, the forebrain splits into two areas: telencephalon and diencephalon. In the diencephalon, the eye field becomes two optic vesicles, which eventually form the optic cups. These optic cups produce the retinal pigment epithelium (RPE) and the neural retina (NR)(Muller and O'Rahilly 1985). Several organoids models have been generated to model human retina and have been summarized elsewhere (O'Hara-Wright and Gonzalez-Cordero 2020). The complex 3-dimensional (3D) architecture of the retina makes it difficult to produce a retina-on-a-chip (RoC). However, we can find several examples of RoC models ((Mut et al. 2022; Thakur et al. 2018{DePamphilis, 2022 #425)}. Even retinal organoids still cannot completely represent the basic functionality of the retina the constant advances in the field make that this could be overcome with the time.

Thyroid MPS

It has been demonstrated that Thyroid organoids can provide functional structures that produce thyroid hormones like triiodothyronine (T3), (Antonica et al. 2012; Kurmann et al. 2015; Ma et al. 2015a; Ma et al. 2015b; Ma et al. 2015c) and can also regenerate tissue when transplanted into thyroid-ablated mice (Romitti et al. 2022). Thyroid organoids cultured can be maintained healthy and improve the production of hormones, thus producing more physiologically relevant results when media flow is well-controlled. In some cases, the organoids functionality has been assessed by ultimately determining the release of hormones such as T3. Several assays can be done to determine the secretion of hormones. For example, determining the capability of the organoids to synthesize thyroglobulin (via immunofluorescence analysis of enriched follicles), and later the iodine uptake using an iodide organification assay. The release of hormones (i.e., T3) is determined by measuring free T3 in culture supernatants under the absence and presence of the Thyroid Stimulating hormone (TSH). Other methods to determine functionality includes RT-PCR analysis to evaluate the expression of key thyroid genes and the effect of flow in the production of hormones by immunostaining against the hormone thyroxine (T4).

Fetal Structures MPS

Studying the fetal structures such as the feto-maternal (FM) interface has a few complications which include, among others, the lack of human subjects and the lack of good in vitro models. Therefore, the development of MPS with fetal structures can provide better interactions between cells of the fetal membrane (Richardson et al. 2020a; Richardson et al. 2020b; Yin et al. 2019; Zhu et al. 2018). Cells in the placenta includes decidua cells, immune cells, trophoblasts, mesenchymal, fibroblasts and amnion epithelial cells. However, for the most part, the cells used in the few examples of FM-MPS are combinations of decidual, immune cells (macrophages) and amnion epithelial cells, and the trophoblasts (BeWo) and endothelial cells. Analytical methods to assess the functionality of the platforms are methods such as membrane permeability assays (fluorescent dye perfusion through the membrane), RNA isolation and quantitative real-time PCR, immunofluorescent staining and imaging, live/dead cells assay, cellular reactive-oxygen species (ROS) detection, adhesion of macrophages, and senescence-associated b-galactosidase biomarker assay. The use of MPS for this organ model is still at its infancy and further research is needed to provide a more complete and effective platform to study pregnancy and drug responses that could translate from pre-clinical testing into clinical trials.

Lymph Node MPS

The lymph node is another complex organ with a large number of cells that comprise the different structures within the organ. The cells in this complex organ include B cells, T cells dendritic cells, plasma cells, fibroblastic reticular cells, folicullar dendritic cells and lymphatic endothelial cells (Ozulumba et al. 2023). Because of its complexity an Organ-on-a-Chip model of the lymph node has not yet been fully realized, with the closest being an example that shows a device that can replicate the spatial microenvironment of a human lymph node (Shanti et al. 2020). It is expected that the same way cells from the lymph nodes, i.e., ex vivo and in vitro, are evaluated, cells within MPS structures could be assessed. That is using techniques such as microscopy, flow cytometry, cell sorting, immunoassays, and nucleic acid analysis (Clark 1962; Crissman and Steinkamp 1973; Saiki et al. 1989) among others.

Ovary MPS

This organ model has seen only a handful of examples of MPS (Frances-Herrero et al. 2022). One of the first research studies with oocytes focused on providing a platform for *in vitro* fertilization (IVF) by designing a microfluidic device that would allow for the exchange of nutrients without the removal of the oocyte from the integrated well (Han et al. 2010). In this case, the method to monitor the functionality of the approach was by optimal imaging and tracking the development of the zygote from

one cell to multiple ones up to the formation of a blastocyst. Methods to assess the successful culture and efficacy of ovarian cells have commonly relied on approaches such as determining the follicle growth, hormonal secretion (i.e., 17b-oestradiol (E2) and progesterone (P4), cytokine expression (e.g., interleukin 8 (IL8), and vascular endothelial growth factor A (VEGF-A))(Xiao et al. 2017). In addition, several organoid systems to study the human female reproductive tract and pregnancy have been developed, and summarized elsewhere (Alzamil et al. 2021).

Bone marrow MPS

Bone marrow (BM) models have been commonly applied to study the biology of bone marrow under physiological and pathological conditions, as well as for drug assays for efficacy and toxicity (Thon et al. 2014; Torisawa et al. 2014; Zhang et al. 2014). BM MPS have been demonstrated with a number of cells found in the bone marrow such as stem and progenitors, osteoblasts, hematopoietic, endothelial, erythroid, myeloid and megakaryocyte cells. The materials commonly used to adhere and grow those, and other bone marrow cells include type I Collagen, alginate and fibronectin. Other materials such as fibrinogen, Matrigel, HA gelatin and decellularized bone have also been used successfully.

References.

Alzamil L, Nikolakopoulou K, Turco MY (2021) Organoid systems to study the human female reproductive tract and pregnancy. Cell Death Differ 28(1):35-51 doi:10.1038/s41418-020-0565-5

Antonica F, Kasprzyk DF, Opitz R, et al. (2012) Generation of functional thyroid from embryonic stem cells. Nature 491(7422):66-71 doi:10.1038/nature11525

Clark SL, Jr. (1962) The reticulum of lymph nodes in mice studied with the electron microscope. Am J Anat 110:217-57 doi:10.1002/aja.1001100303

Crissman HA, Steinkamp JA (1973) Rapid, Simultaneous Measurement of DNA, Protein, and Cell Volume in Single Cells from Large Mammalian-Cell Populations. J Cell Biol 59(3):766-771 doi:DOI 10.1083/jcb.59.3.766

Frances-Herrero E, Lopez R, Hellstrom M, et al. (2022) Bioengineering trends in female reproduction: a systematic review. Hum Reprod Update 28(6):798-837 doi:10.1093/humupd/dmac025

Han C, Zhang Q, Ma R, et al. (2010) Integration of single oocyte trapping, in vitro fertilization and embryo culture in a microwell-structured microfluidic device. Lab Chip 10(21):2848-54 doi:10.1039/c005296e

Kieninger J, Weltin A, Flamm H, Urban GA (2018) Microsensor systems for cell metabolism - from 2D culture to organ-on-chip. Lab Chip 18(9):1274-1291 doi:10.1039/c7lc00942a

Kurmann AA, Serra M, Hawkins F, et al. (2015) Regeneration of Thyroid Function by Transplantation of Differentiated Pluripotent Stem Cells. Cell Stem Cell 17(5):527-542 doi:10.1016/j.stem.2015.09.004

Ma RS, Latif R, Davies TF (2015a) Human Embryonic Stem Cells Form Functional Thyroid Follicles. Thyroid 25(4):455-461 doi:10.1089/thy.2014.0537

Ma RS, Morshed SA, Latif R, Davies TF (2015b) Thyroid cell differentiation from murine induced pluripotent stem cells. Front Endocrinol 6 doi:ARTN 56

10.3389/fendo.2015.00056
Ma Z, Wang J, Loskill P, et al. (2015c) Self-organizing human cardiac microchambers mediated by geometric confinement. Nat Commun 6:7413 doi:10.1038/ncomms8413
Mori N, Morimoto Y, Takeuchi S (2017) Skin integrated with perfusable vascular channels on a chip. Biomaterials 116:48-56 doi:10.1016/j.biomaterials.2016.11.031
Muller F, O'Rahilly R (1985) The first appearance of the neural tube and optic primordium in the human embryo at stage 10. Anat Embryol (Berl) 172(2):157-69 doi:10.1007/BF00319598
Mut SR, Mishra S, Vazquez M (2022) A Microfluidic Eye Facsimile System to Examine the Migration of Stem-like Cells. Micromachines (Basel) 13(3) doi:10.3390/mi13030406
O'Hara-Wright M, Gonzalez-Cordero A (2020) Retinal organoids: a window into human retinal development. Development 147(24) doi:10.1242/dev.189746
Ozulumba T, Montalbine AN, Ortiz-Cardenas JE, Pompano RR (2023) New tools for immunologists: models of lymph node function from cells to tissues. Front Immunol 14 doi:ARTN 1183286

10.3389/fimmu.2023.1183286
Ramadan Q, Ting FCW (2016) In vitro micro-physiological immune-competent model of the human skin. Lab Chip 16(10):1899-1908 doi:10.1039/c6lc00229c
Richardson L, Gnecco J, Ding T, et al. (2020a) Fetal Membrane Organ-On-Chip: An Innovative Approach to Study Cellular Interactions. Reprod Sci 27(8):1562-1569 doi:10.1007/s43032-020-00184-9
Richardson L, Kim S, Menon R, Han A (2020b) Organ-On-Chip Technology: The Future of Feto-Maternal Interface Research? Front Physiol 11:715 doi:10.3389/fphys.2020.00715
Romitti M, Tourneur A, da Fonseca BD, et al. (2022) Transplantable human thyroid organoids generated from embryonic stem cells to rescue hypothyroidism. Nature Communications 13(1) doi:ARTN 7057

10.1038/s41467-022-34776-7
Saiki RK, Walsh PS, Levenson CH, Erlich HA (1989) Genetic-Analysis of Amplified DNA with Immobilized Sequence-Specific Oligonucleotide Probes. P Natl Acad Sci USA 86(16):6230-6234 doi:DOI 10.1073/pnas.86.16.6230
Shanti A, Samara B, Abdullah A, et al. (2020) Multi-Compartment 3D-Cultured Organ-on-a-Chip: Towards a Biomimetic Lymph Node for Drug Development. Pharmaceutics 12(5) doi:ARTN 464

10.3390/pharmaceutics12050464
Srinivasan B, Kolli AR, Esch MB, Abaci HE, Shuler ML, Hickman JJ (2015) TEER Measurement Techniques for In Vitro Barrier Model Systems. Jala-J Lab Autom 20(2):107-126 doi:10.1177/2211068214561025
Sriram G, Alberti M, Dancik Y, et al. (2018) Full-thickness human skin-on-chip with enhanced epidermal morphogenesis and barrier function. Mater Today 21(4):326-340 doi:10.1016/j.mattod.2017.11.002
Thakur A, Mishra S, Pena J, et al. (2018) Collective adhesion and displacement of retinal progenitor cells upon extracellular matrix substrates of transplantable biomaterials. J Tissue Eng 9:2041731417751286 doi:10.1177/2041731417751286
Thon JN, Mazutis L, Wu S, et al. (2014) Platelet bioreactor-on-a-chip. Blood 124(12):1857-67 doi:10.1182/blood-2014-05-574913
Tokuyama E, Nagai Y, Takahashi K, Kimata Y, Naruse K (2015) Mechanical Stretch on Human Skin Equivalents Increases the Epidermal Thickness and Develops the Basement Membrane. Plos One 10(11) doi:ARTN e0141989

10.1371/journal.pone.0141989
Torisawa YS, Spina CS, Mammoto T, et al. (2014) Bone marrow-on-a-chip replicates hematopoietic niche physiology in vitro. Nat Methods 11(6):663-9 doi:10.1038/nmeth.2938

Tremblay D, Chagnon-Lessard S, Mirzaei M, Pelling AE, Godin M (2014) A microscale anisotropic biaxial cell stretching device for applications in mechanobiology. Biotechnol Lett 36(3):657-665 doi:10.1007/s10529-013-1381-5

Xiao S, Coppeta JR, Rogers HB, et al. (2017) A microfluidic culture model of the human reproductive tract and 28-day menstrual cycle. Nat Commun 8:14584 doi:10.1038/ncomms14584

Yin FC, Zhu YJ, Zhang M, Yu H, Chen WW, Qin JH (2019) A 3D human placenta-on-a-chip model to probe nanoparticle exposure at the placental barrier. Toxicology in Vitro 54:105-113 doi:10.1016/j.tiv.2018.08.014

Zhang W, Lee WY, Siegel DS, Tolias P, Zilberberg J (2014) Patient-specific 3D microfluidic tissue model for multiple myeloma. Tissue Eng Part C Methods 20(8):663-70 doi:10.1089/ten.TEC.2013.0490

Zhang YS, Aleman J, Shin SR, et al. (2017) Multisensor-integrated organs-on-chips platform for automated and continual in situ monitoring of organoid behaviors. P Natl Acad Sci USA 114(12):E2293-E2302 doi:10.1073/pnas.1612906114

Zhu YY, Yin FC, Wang H, Wang L, Yuan JL, Qin JH (2018) Placental Barrier-on-a-Chip: Modeling Placental Inflammatory Responses to Bacterial Infection. Acs Biomater Sci Eng 4(9):3356-3363 doi:10.1021/acsbiomaterials.8b00653

Supplementary material 10

Considerations for Multi-MPS

The need to decipher in vivo-relevant multi-organ interactions led to the establishment/generation of Multi-MPS. These systems aroused to mimic the interactions between different organs at a systemic level as alternatives to animal testing (Schneider et al. 2021; Watson et al. 2017). Apart from physiological interactions, a multi-organ setting can recapitulate absorption, distribution, metabolism and excretion (ADME) properties, pharmaco- and toxicokinetics, as well as pharmaco- and toxicodynamics of a compound and its metabolite (Wang et al. 2018). Added complexity, by e.g., the integration of lung, skin and intestinal models, allows identifying administration route dependent metabolic and PK/PD characteristics (Kuhnl et al. 2021). From a drug discovery point of view, multi-MPS can be utilized for predictive dose range finding studies taking both route of administration into account, on- and off-target effects, inter-organ metabolism of drugs (Ma et al. 2021; Singh et al. 2022), systemic drug-drug interactions and to show absence of toxicity (Cong et al. 2020; Schneider et al. 2021). Studying the safety, efficacy (Hubner et al. 2018) and ADME properties of drugs interacting with multiple organs has been shown by multiple groups (Cecen et al. 2021; Chen et al. 2017; Prot et al. 2014; Skardal et al. 2017; Tsamandouras et al. 2017). Proof-on-concept studies performed with FDA-recalled drugs on multi-MPS systems were able to recapitulate drug induced organ specific toxicity (Maschmeyer et al. 2015a; Maschmeyer et al. 2015b; Skardal et al. 2017). At the same time from a toxicology point of view, multi-MPS can serve as an ab initio tool at the higher tier for estimation of point of departure, uncertainty, systemic toxicity, and metabolism dependent specific target organ toxicity (STOT) (Berggren et al. 2017; Nitsche et al. 2022).

Quality criteria for a fit-for-purpose multi-MPS assay should take into account several aspects starting with organ and medium scaling challenges. Absolut organ scale in the chip is as important as organ-to-organ ratios in a multiple organ system. Organ model scale in an OoC system at an extreme minimum can impact organ functionality and structure (Picollet-D'hahan et al. 2021) while larger scales increase cost and hinders feasibility. The organoids as the functional minimal unit, are the representative subunits of each organ which should be defined at the assay establishment phase and taken into account during scaling of each organ even as single-MPS (Marx et al. 2012). When it comes to multi-MPS, relative organ size is essential to be able to recapitulate the in vivo-like organ crosstalk, ADME, toxicity response and PBPK/PD modelling (Dehne and Marx 2020; Picollet-D'hahan et al. 2021). Several approaches such as residence time, proportional, allometric, and functional are utilized to scale the organs (Abaci and Shuler 2015; Park et al. 2020; van Midwoud et al. 2010). Each method has its advantages and weaknesses where one approach for all assays may not be feasible and need to be

defined on a case-by-case approach. On the other hand, the volume of medium as a blood surrogate plays a critical role in mass calculations of the compounds where liquid-to-cell ratio ideally be designed closer to in vivo values. A high liquid-to-cell ratio can hinder paracrine signalling by diluting the cytokines produced by the organ models and the metabolites (Sung 2021).

Mathematical models are an integral part of the preclinical studies to translate the in vitro OoC data into in vivo relevant information. In vitro in vivo extrapolation (IVIVE) requires scaling factors to correlate OoC PK models to in vivo organ and systemic relevant data (Yang et al. 2022). A predictive model should take into account the medium content and volume, organ layout, flow rates and characteristics, liquid-to-cell ratio, nature of the model (e.g. barrier, spheroid) transport capacity and non-specific binding characteristics (Abaci and Shuler 2015; Sung 2021).

Reproducible production of co-culture medium with defined factors is well understood to be able to provide reliable cell culture results (Vis et al. 2020). Barrier models in co-culture allow further systemic readouts given the fact that apical side of the model can be cultured with its own medium in contrast to co-culture medium utilized at the basolateral side or in different media gradients such as higher or lower glucose on different side of the barrier. Tracing the active or passive transported molecules of interest through the system via colorimetric, fluorescent or radioactive methods relevant to the functional read out of the model would allow us to monitor the co-culture functionally and viability (Maschmeyer et al. 2015b).

The cell source for the generation of organ models is of high importance when performing multi-MPS cultures taking functionality and genetic background into account as discussed above. For the fit-for-purpose assay, the cell source shall serve the required functionality. On the other hand, once the integration of an immune component becomes relevant for long term multi-MPS cultures, having isogenic models readily available is only feasible by using iPSC-derived organ models (Ramme et al. 2019). Here, protocols for the generation of various immune cell components have been published (Flippe et al. 2020; Iriguchi et al. 2021; Nianias and Themeli 2019). Furthermore, the supplementation of iPSC-derived organ models with primary leukocytes derived from iPSC-donors is possible in principle.

From the organ model perspective, multi-MPS has an additional challenge to determine organ specific readouts during the assay period. Here online monitoring of the systemic circulation play a critical role. For example, LDH can be measured as an online monitoring marker for toxicity in the multi organ conditioned media. However, increased LDH would not specify if a particular organ or several organs are targeted without end-point assays like ATP, MTT and/or TUNEL assays (Materne et al. 2015).

Identifying organ specific factors such as organ specific miRNAs (Keller et al. 2022) released in the media would be the main step for reproducible systemic online monitoring in addition to non-specifically monitored factors. Optical analysis that can be performed during the whole assay time give additional information on organ model behaviour and substance effects.

Furthermore, systemic disease models such as inflammatory diseases, cancer metastasis (e.g. epithelial-mesenchymal transition, migration), immune therapies, metabolic syndrome, diseases that involve the endocrine system e.g. hyperthyroidism/hypothyroidism (Marx et al. 2021; Picollet-D'hahan et al. 2021), diabetes (Bauer et al. 2018; Marx et al. 2020) and cytic fibrosis (Ingber 2022) require organ-organ interaction. Even though the field is moving fast forward in disease modelling, the complexity and diversity of the disease etiologies makes it complex to characterize and set the standards for the assays. A fit-for-purpose assay with the healthy organs of interest in homeostasis to set the basis for organ related read-outs followed by diseased state related readouts should be established (Marx et al. 2021).

In the past decade, multiple groups started to tackle the challenges associated with multi-MPS co-cultures aiming for reliable, reproducible and in vivo-relevant readouts. The final goal of having a systemically interacting human organismoid on-chip integrating all major organ systems remains to be an ambitious challenge, whereat the scientific community no longer doubts the general feasibility (Dehne and Marx 2020; Marx et al. 2021).

**Multi-MPS Quality Parameters**	**Criteria**
Scaling	at absolute organ level<br>organ-to-organ ratio<br>liquid-to-cell ratio
Mathematical model parameterization	medium content and volume, organ layout, flow rates and characteristics, scaling, model nature, transport, non-specific binding
Co-culture media	defined factors
Cell sources	functionally competent<br>isogenic models especially for immune competent systems
Online monitoring	general markers<br>organ specific markers
Disease models	readout establishment in comparison to healthy model

References.

Abaci HE, Shuler ML (2015) Human-on-a-chip design strategies and principles for physiologically based pharmacokinetics/pharmacodynamics modeling. Integr Biol (Camb) 7(4):383-91 doi:10.1039/c4ib00292j

Bauer S, Huldt CW, Kanebratt KP, et al. (2018) Functional coupling of human pancreatic islets and liver spheroids on-a-chip: Towards a novel human ex vivo type 2 diabetes model (vol 7, 2017). Sci Rep-Uk 8 doi:ARTN 1672

10.1038/s41598-018-20340-1

Berggren E, White A, Ouedraogo G, et al. (2017) Ab initio chemical safety assessment: A workflow based on exposure considerations and non-animal methods. Comput Toxicol 4:31-44 doi:10.1016/j.comtox.2017.10.001

Cecen B, Karavasili C, Nazir M, et al. (2021) Multi-Organs-on-Chips for Testing Small-Molecule Drugs: Challenges and Perspectives. Pharmaceutics 13(10) doi:10.3390/pharmaceutics13101657

Chen WLK, Edington C, Suter E, et al. (2017) Integrated gut/liver microphysiological systems elucidates inflammatory inter-tissue crosstalk. Biotechnol Bioeng 114(11):2648-2659 doi:10.1002/bit.26370

Cong Y, Han X, Wang Y, et al. (2020) Drug Toxicity Evaluation Based on Organ-on-a-chip Technology: A Review. Micromachines (Basel) 11(4) doi:10.3390/mi11040381

Dehne EM, Marx U (2020) The universal physiological template-a system to advance medicines. Curr Opin Toxicol 23-24:1-5 doi:10.1016/j.cotox.2020.02.002

Flippe L, Gaignerie A, Serazin C, et al. (2020) Rapid and Reproducible Differentiation of Hematopoietic and T Cell Progenitors From Pluripotent Stem Cells. Front Cell Dev Biol 8:577464 doi:10.3389/fcell.2020.577464

Hubner J, Raschke M, Rutschle I, et al. (2018) Simultaneous evaluation of anti-EGFR-induced tumour and adverse skin effects in a microfluidic human 3D co-culture model. Sci Rep 8(1):15010 doi:10.1038/s41598-018-33462-3

Ingber DE (2022) Human organs-on-chips for disease modelling, drug development and personalized medicine. Nat Rev Genet 23(8):467-491 doi:10.1038/s41576-022-00466-9

Iriguchi S, Yasui Y, Kawai Y, et al. (2021) A clinically applicable and scalable method to regenerate T-cells from iPSCs for off-the-shelf T-cell immunotherapy. Nat Commun 12(1):430 doi:10.1038/s41467-020-20658-3

Keller A, Groger L, Tschernig T, et al. (2022) miRNATissueAtlas2: an update to the human miRNA tissue atlas. Nucleic Acids Res 50(D1):D211-D221 doi:10.1093/nar/gkab808

Kuhnl J, Tao TP, Brandmair K, et al. (2021) Characterization of application scenario-dependent pharmacokinetics and pharmacodynamic properties of permethrin and hyperforin in a dynamic skin and liver multi-organ-chip model. Toxicology 448:152637 doi:10.1016/j.tox.2020.152637

Ma C, Peng Y, Li H, Chen W (2021) Organ-on-a-Chip: A New Paradigm for Drug Development. Trends Pharmacol Sci 42(2):119-133 doi:10.1016/j.tips.2020.11.009

Marx U, Accastelli E, David R, et al. (2021) An Individual Patient's "Body" on Chips-How Organismoid Theory Can Translate Into Your Personal Precision Therapy Approach. Front Med (Lausanne) 8:728866 doi:10.3389/fmed.2021.728866

Marx U, Akabane T, Andersson TB, et al. (2020) Biology-inspired microphysiological systems to advance patient benefit and animal welfare in drug development. ALTEX 37(3):365-394 doi:10.14573/altex.2001241

Marx U, Walles H, Hoffmann S, et al. (2012) 'Human-on-a-chip' developments: a translational cutting-edge alternative to systemic safety assessment and efficiency evaluation of substances in laboratory animals and man? Altern Lab Anim 40(5):235-57 doi:10.1177/026119291204000504

Maschmeyer I, Hasenberg T, Jaenicke A, et al. (2015a) Chip-based human liver-intestine and liver-skin co-cultures--A first step toward systemic repeated dose substance testing in vitro. Eur J Pharm Biopharm 95(Pt A):77-87 doi:10.1016/j.ejpb.2015.03.002
Maschmeyer I, Lorenz AK, Schimek K, et al. (2015b) A four-organ-chip for interconnected long-term co-culture of human intestine, liver, skin and kidney equivalents. Lab Chip 15(12):2688-99 doi:10.1039/c5lc00392j
Materne EM, Maschmeyer I, Lorenz AK, et al. (2015) The multi-organ chip--a microfluidic platform for long-term multi-tissue coculture. J Vis Exp(98):e52526 doi:10.3791/52526
Nianias A, Themeli M (2019) Induced Pluripotent Stem Cell (iPSC)-Derived Lymphocytes for Adoptive Cell Immunotherapy: Recent Advances and Challenges. Curr Hematol Malig Rep 14(4):261-268 doi:10.1007/s11899-019-00528-6
Nitsche KS, Muller I, Malcomber S, Carmichael PL, Bouwmeester H (2022) Implementing organ-on-chip in a next-generation risk assessment of chemicals: a review. Arch Toxicol 96(3):711-741 doi:10.1007/s00204-022-03234-0
Park D, Lee J, Chung JJ, Jung Y, Kim SH (2020) Integrating Organs-on-Chips: Multiplexing, Scaling, Vascularization, and Innervation. Trends Biotechnol 38(1):99-112 doi:10.1016/j.tibtech.2019.06.006
Picollet-D'hahan N, Zuchowska A, Lemeunier I, Le Gac S (2021) Multiorgan-on-a-Chip: A Systemic Approach To Model and Decipher Inter-Organ Communication. Trends Biotechnol 39(8):788-810 doi:10.1016/j.tibtech.2020.11.014
Prot JM, Maciel L, Bricks T, et al. (2014) First pass intestinal and liver metabolism of paracetamol in a microfluidic platform coupled with a mathematical modeling as a means of evaluating ADME processes in humans. Biotechnol Bioeng 111(10):2027-40 doi:10.1002/bit.25232
Ramme AP, Koenig L, Hasenberg T, et al. (2019) Autologous induced pluripotent stem cell-derived four-organ-chip. Future Sci OA 5(8):FSO413 doi:10.2144/fsoa-2019-0065
Schneider MR, Oelgeschlaeger M, Burgdorf T, et al. (2021) Applicability of organ-on-chip systems in toxicology and pharmacology. Crit Rev Toxicol 51(6):540-554 doi:10.1080/10408444.2021.1953439
Singh D, Mathur A, Arora S, Roy S, Mahindroo N (2022) Journey of organ on a chip technology and its role in future healthcare scenario. Appl Surf Sci Adv 9 doi:ARTN 100246

10.1016/j.apsadv.2022.100246
Skardal A, Murphy SV, Devarasetty M, et al. (2017) Multi-tissue interactions in an integrated three-tissue organ-on-a-chip platform. Sci Rep 7(1):8837 doi:10.1038/s41598-017-08879-x
Sung JH (2021) Multi-organ-on-a-chip for pharmacokinetics and toxicokinetic study of drugs. Expert Opin Drug Metab Toxicol 17(8):969-986 doi:10.1080/17425255.2021.1908996
Tsamandouras N, Chen WLK, Edington CD, Stokes CL, Griffith LG, Cirit M (2017) Integrated Gut and Liver Microphysiological Systems for Quantitative In Vitro Pharmacokinetic Studies. AAPS J 19(5):1499-1512 doi:10.1208/s12248-017-0122-4
van Midwoud PM, Merema MT, Verpoorte E, Groothuis GM (2010) A microfluidic approach for in vitro assessment of interorgan interactions in drug metabolism using intestinal and liver slices. Lab Chip 10(20):2778-86 doi:10.1039/c0lc00043d
Vis MAM, Ito K, Hofmann S (2020) Impact of Culture Medium on Cellular Interactions in in vitro Co-culture Systems. Front Bioeng Biotechnol 8:911 doi:10.3389/fbioe.2020.00911
Wang YI, Carmona C, Hickman JJ, Shuler ML (2018) Multiorgan Microphysiological Systems for Drug Development: Strategies, Advances, and Challenges. Adv Healthc Mater 7(2) doi:10.1002/adhm.201701000
Watson DE, Hunziker R, Wikswo JP (2017) Fitting tissue chips and microphysiological systems into the grand scheme of medicine, biology, pharmacology, and toxicology. Exp Biol Med (Maywood) 242(16):1559-1572 doi:10.1177/1535370217732765
Yang Y, Chen Y, Wang L, et al. (2022) PBPK Modeling on Organs-on-Chips: An Overview of Recent Advancements. Front Bioeng Biotechnol 10:900481 doi:10.3389/fbioe.2022.900481
